# Cognitive-Based Interventions for Improving Psychological Health and Well-Being for Parents of Children with Developmental Disabilities: A Systematic Review and Meta-analysis

**DOI:** 10.1007/s10803-023-06063-x

**Published:** 2023-09-05

**Authors:** Sini Li, Yijing Yong, Yamin Li, Jianhe Li, Jiao Xie

**Affiliations:** 1 The Nethersole School of Nursing, Faculty of Medicine, The Chinese University of Hong Kong, Hong Kong, China; 2 Clinical Nursing Teaching and Research Section, The Second Xiangya Hospital, Central South University, 139 Renmin Middle Road, Changsha, 410011 China; 3 Cognition and Human Behaviour Key Laboratory of Hunan Province, Hunan Normal University, Changsha, China; 4 Department of Pharmacy, The Second Xiangya Hospital, Central South University, 139 Renmin Middle Road, Changsha, 410011 China; 5 Department of Otolaryngology-Head and Neck Surgery, The Second Xiangya Hospital, Central South University, 139 Renmin Middle Road, Changsha, 410011 Hunan China

**Keywords:** Developmental disabilities, Parents, Cognitive behavioural therapy, Mindfulness, Meta-analysis

## Abstract

This review aims to systematically summarize existing evidence to determine the effectiveness of cognitive-based interventions (CBIs) on psychological health and well-being among parents of children with developmental disabilities (DD). Six databases were searched to identify eligible randomized controlled trials (RCTs) from their inception to April 2023. The revised Cochrane Risk of Bias tool for RCTs was applied to assess the risk of bias and the certainty of evidence was evaluated using the Grading of Recommendation, Assessment, Development and Evaluation. Meta-analyses were conducted using a random-effects model. Twenty-five RCTs involving 1915 participants were identified. The results indicated that CBIs reduced parental stress levels (Hedges’ g = − 0.69), depressive symptoms (g = − 0.95), anxiety levels (g = − 0.78), and parental distress (g = − 0.29), and improved parental well-being (g = 0.62) and parent‒child relationships (g = 0.43) postintervention compared with the active/inactive control groups. Subgroup analysis of the effectiveness of interventions using mindfulness-based interventions and cognitive behavioural therapy showed positive effects. The favourable intervention duration and participant targets were also identified in this review. Furthermore, the effects of CBIs were impacted by the different types of DD among the children. This review highlighted the positive effects of CBIs on parental stress levels, depressive symptoms, anxiety levels, parental distress levels, parental well-being levels, and parent‒child relationships. Future well-designed RCTs are needed to further investigate the effects of MBIs and CBT interventions on children with DD and their parents, as well as the factors and mechanisms of action affecting the efficacy of these interventions.

**Supplementary Information:**

The online version contains supplementary material available at 10.1007/s10803-023-06063-x.

## Introduction

Developmental disorders (DD) are a set of lifelong disorders (e.g., attention-deficit/hyperactivity disorder [ADHD], autism spectrum disorder [ASD], cerebral palsy [CP], fragile X syndrome, developmental delay, intellectual disabilities [ID], learning disorders, language disorders, and Tourette syndrome) that originate in childhood and cause severe impairment in daily function in different domains (e.g., physical, learning, language, emotional or behavioural function) (Holm, 1989; Prevention, 2022). Previous nationwide surveys in the United States discovered that the prevalence of DD has risen from 12.84 to 17.76% in the last 20 years (Boyle et al., 2011; Zablotsky et al., 2019). Given this growth, substantial care needs, support, and accessibility of resources and interventions are typically required for children with DD and their families, enhancing long-term outcomes for those children and families (Baio et al., 2018; Vohra et al., 2014).

Parenting a child with DD is exhausting, and it imposes many challenges on parents because they need to invest considerable time, energy, and money to support and nurture their child’s healthy development (Resch et al., 2010). Due to the specialized care needs of these children, parents have observed amplified levels of emotional distress (Robinson et al., 2018), also known as stress (Osmančević Katkić et al., 2017), anxiety (Bujnowska et al., 2019), and depression (Scherer et al., 2019), as well as impairments in other health outcomes, such as physical function (Cantwell et al., 2014), social function (Ali et al., 2012), and general well-being (Baker et al., 2005) or quality of life (Arora et al., 2020). Concerningly, these psychological issues can have a detrimental impact on parental well-being and parenting practice (Neece et al., 2012), resulting in poor child, parent, and family outcomes in families with children with DD due to bidirectional relationships between parent emotion and child outcomes (Woodman et al., 2015).

To support the parents of children with DD and improve their psychological well-being, a variety of cognitive-based interventions (CBIs), including cognitive-based therapy (CBT), dialectical behavioural therapy (DBT), mindfulness-based intervention (MBIs), acceptance and commitment therapy (ACT), and compassion-focused therapy (CFT), have been recently developed. Based on the concept that thoughts, feelings, and behaviours are interrelated, CBT employs cognitive methods to change maladaptive beliefs and cognitive distortions as well as modify problematic behavioural patterns that maintain stress/distress (Beck & Haigh, 2014). Moreover, MBIs, which include mindfulness-based stress reduction (MBSR) and mindfulness-based cognitive therapy (MBCT), can be defined as the practice of mindfulness that improves participants’ attention/awareness of the present moment in a nonjudgmental manner, allowing them to better cope with life stressors and enhance their general well-being (Kabat-Zinn, 2003). The first application of these ideas to psychotherapy was developed by Dr. Jon Kabat-Zinn in the 1970s by first applying MBSR in the Western world (Kabat-Zinn & Hanh, 2009). The principle of MBSR has been further adapted in MBCT, which combines the mindfulness practices of MBSR with the concepts of CBT to treat the process of negative thinking and feeling rather than the content of psychological symptoms (Chadwick et al., 2016), or ACT, which aims to improve affective symptoms by promoting psychological flexibility, identifying personal values, and managing commitments to make adjustments (Hayes & Strosahl, 2005; Hayes et al., 1999). In addition, as part of third-wave CBT, DBT combines various strategies (e.g., mindfulness, emotion regulation, awareness, and acceptance) that enable clients to utilize new skills/strategies to build lives that they feel worthwhile. Furthermore, as newly developed CBI, CFT incorporates mindfulness to assist in enabling mental and emotional recovery by encouraging participants to be compassionate towards themselves and others (Gilbert, 2009; Khoshvaght et al., 2021).

These CBIs have reported promising results in lessening stress, depressive symptoms, and anxiety across parents of children with special needs (Bourke-Taylor et al., 2021; Parmar et al., 2021), further confirming their ability to help enhance the psychological well-being of parents of children with DD. Previous systematic reviews and meta-analyses reported significant improvements in the psychosocial outcomes of caregivers of children with ASD with CBIs (Yu et al., 2019). This finding was further confirmed by Bourke-Taylor et al. (2021), who also revealed the positive effects of CBT on parental stress and mental health for parents of children with DD. Several systematic reviews demonstrated that MBIs and/or ACT were superior to the control group regarding postintervention mental health and subjective well-being results for parents of children with different types of DD (Chua & Shorey, 2022; Hartley et al., 2019; Juvin et al., 2022; Lee et al., 2022; Yu et al., 2019).

While the reviews mentioned above offer preliminary evidence on the effectiveness of CBIs for parents of children with DD, these studies contain significant methodological limitations. First, the included studies in these reviews used a wide range of study designs, including single-group, pre- and post-test studies, randomized controlled trials, and nonrandomized controlled trials (Hartley et al., 2019; Juvin et al., 2022; Lee et al., 2022; Yu et al., 2019). Therefore, those syntheses might provide low-quality evidence with a significant risk of bias and unreliable outcomes. In addition, the majority of previous reviews included a limited number of studies and small sample sizes (lower than 1,000 participants), making it difficult to draw solid conclusions (Cachia et al., 2016; Hartley et al., 2019; Juvin et al., 2022; Lee et al., 2022; Osborn et al., 2021). Furthermore, the study samples were very heterogeneous, including children and adults with DD (Hartley et al., 2019; Juvin et al., 2022), and did not analyse outcomes separately for parents of children with different types of DD (Bourke-Taylor et al., 2021; Chua & Shorey, 2022; Osborn et al., 2021).

Considering the heavy caregiver burden of those parents, the limitations of the systematic and meta-analysis demonstrated above, and the potential benefits of CBIs for parents of children with DD to improve their mental health, it is paramount to synthesize the existing evidence on the use of CBIs to enhance parents’ psychological well-being in while caring for children with DD to drive future evidence-based research and practice. Moreover, it is still inconclusive about the best effective approach and optimal characteristics of CBIs for improving those parents’ health outcomes. Therefore, this review aimed to comprehensively summarize the effects of CBI with respect to psychological problems and well-being among parents of children with DD, when compared with active/inactive controls, and investigate the optimal features of the effective interventions found.

## Methods

This review was registered with PROSERO registration number CRD42022382502 and conducted based on the Preferred Reporting Items for Systematic Reviews and Meta-Analysis statement (PRISMA) (Moher et al., 2009). Two reviewers (SNL and YJY) independently conducted certain phases of systematic review, including study selection, data extraction, and quality appraisals. When confronted with a conflict, two reviewers either discussed or consulted with the third reviewer (YML) to achieve an agreement.

### Research Strategy

Six English databases, including PubMed, Embase (Ovid), PsycINFO, CINAHL, Cochrane Central Register, and ProQuest, were systematically searched from inception to December 2022. The PICOS (population, intervention, comparisons, outcomes, study) framework listed in Appendix S1 was used to select MeSH terms and keywords, including types of participants (e.g., caregiver*, parent*, mother*, father*, maternal*, famil*), types of diagnoses (e.g., developmental disabilities, autism spectrum disorder, attention deficit disorder with hyperactivity, fragile x syndrome, Down syndrome), types of interventions (e.g., cognitive behavio?ral therap*, acceptance and commitment therapy, mindful, mindfulness-based, dialectical behavioural therapy, metacognitive), and types of psychosocial outcomes (e.g., stress, anxiety, depressi*, parenting distress). The search strategy was designed for PubMed and then adapted for other databases. The research strategies and results for all the databases are listed in Supplementary Tables 2–7. Moreover, the reference lists of pertinent publications and internet search engines, such as Google Scholar, were manually searched for additional relevant articles.

### Inclusion and Exclusion Criteria

Studies were included when they met the following criteria:

1. Populations: The participants were parents (aged > 18) of children (aged ≤ 18) diagnosed with DD (according to the World Health Organization (Almeida et al., 2020), the diagnoses for DD include a diverse group of conditions characterized by impairments in physical ability, learning, language and/or behaviour, such as autism spectrum disorder [ASD], attention-deficit/hyperactivity disorder [ADHD], fragile X syndrome, Down syndrome, intellectual disabilities).
2. Interventions: The studies explicitly and independently referred to CBIs, including CBT, MBIs, ACT, DBT, or self-compassion therapy, in their description of the primary part of the intervention.
3. Comparison: The CBIs were compared to both active (e.g., attention care) and inactive (e.g., treat as usual [TAU], standard care, waitlist, no treatment, placebo) control groups.
4. Outcomes: The parental psychological outcomes (including emotional distress, stress, depression, and anxiety) were measured by valid instruments at least at the time of postintervention.
5. Study design: Only randomized controlled trials (RCTs) published in peer-reviewed journals and (6) only studies in English were eligible.

Studies were excluded when (1) the primary outcomes of interventions were focused on children with DD (e.g., child-focused or parent-mediated interventions) rather than their parents; (2) multicomponent interventions (such as combined CBIs with behavioural training) were considered the major component in the intervention group; (3) the group sample size for those receiving treatment was lower than five; and (4) full-text or final results were unavailable (e.g., conference abstracts and protocols).

### Study Selection

To check for duplicate publications, all retrieved records were loaded into Covidence software. The abstracts and titles were separately reviewed by two reviewers (SNL and YJY) in accordance with the eligibility criteria. Eligible articles were eventually identified after examining the full texts of possibly relevant research.

### Data Extraction

A self-developed data extraction form was applied based on the Cochrane data collection form for RCT review (Sambunjak D et al., 2017). The form was piloted on five randomly selected eligible articles and revised accordingly. Two reviewers extracted all necessary data independently, and the following information was collected: (1) the basic information of the studies (including the last names of the authors, the year of publication, and the locations, study designs, and sample sizes); (2) participant characteristics (including the age range and the sex proportion for the children with DD and their parents); (3) characteristics of the interventions (including the types of cognitive-based interventions and the comparators, intervention contexts, research formats, dosages, durations, and lengths of follow-ups); and (4) outcome measures (including measurement tools, assessment time-point(s), and outcomes [effect sizes]). Two reviewers (SNL and YJY) extracted data individually, and any differences of opinion were resolved by discussion or contact with the third reviewer (JHL).

The first author contacted the corresponding authors to provide additional information if the information provided was not sufficiently detailed (such as using figures to report the outcomes and not revealing the specific data in the article). When authors did not reply after being contacted twice through email a month apart, the articles were removed from consideration.

### Quality Appraisal

The revised Cochrane Risk of Bias tool (RoB2) for RCTs (Sterne et al., 2019) was applied to assess the quality of each included trial. Any disagreements between critical appraisers were resolved by discussion and consultation with the third reviewer. Five categories of bias are evaluated by the RoB: selection (random sequence generation and allocation concealment), performance (blinding of participants and personnel), attrition (incomplete outcome data), detection (blinding of outcome assessment), and reporting bias (selective reporting). Bias was rated as “low,” “unclear,” or “high” for each domain for each study. The proportion of research that met each quality rating was then calculated.

### Certainty of Evidence

A narrative assessment of the degree of certainty of the evidence was presented using the Grading of Recommendation, Assessment, Development, and Evaluation (GRADE) system (Balshem et al., 2011). Based on the five GRADE domains—methodological limitations of the studies or bias risk, indirectness, imprecision, consistency, and publication bias—two independent appraisers assessed the degree of certainty of the evidence and categorized it into four categories: high, moderate, low, and very low.

### Data Synthesis

R software was applied to assess the statistical meta-analysis by computing the Hedges’ g standard mean differences (SMDs) with 95% confidence intervals (CIs) for each study. Given the expected variability between studies and outcomes, the random-effects model was chosen to pool the SMDs across the studies. The effect sizes (ESs) were primarily assessed postintervention; specifically, the time-point was less than four weeks after the intervention. Based on Cohen’s categories, ESs were classified as small (g = 0.2), medium (g = 0.5), or large (g ≥ 0.8) (Lipsey & Wilson, 2001). Moreover, only outcomes from at least two or more studies were pooled in the model for the meta-analysis as well as for the subgroup analyses. The meta-analysis included three major subgroup analyses to investigate whether efficacy varied among intervention approaches, durations (1–8 weeks and over eight weeks), targeted participants (only parent-involved versus parent‒child dyads), and the types of DD among the children. A minimum of two trials per subgroup were required for the subgroup analyses (Deeks & Altman, 2022). Furthermore, several change scores, such as those associated with the parent‒child relationship, parental well-being, mindful parenting, mindful awareness, and psychological flexibility, were reversed for clarity, so that positive ESs were always associated with positive clinical results.

Heterogeneity was assessed using I^2^ and Cochrane’s Q. I^2^ values of 25, 50, and 75% were considered low, moderate, and high heterogeneity, respectively. Cochran’s Q is the standard test statistic that reveals systematic between-study differences. Sensitivity analyses were undertaken to explore whether the results were drastically affected by excluding trials with highly disparate ESs. If meta-analyses contained more than 10 studies, the assessments of publication bias were performed by visually evaluating Egger’s statistical test and funnel plots. An asymmetry map highlights likely missing research as a result of publication bias. In the case of Egger’s test, a P value of < 0.05 was used to establish statistical evidence of asymmetry (Egger et al., 1997). Outliers in which the 95% CI was outside the 95% CI of the overall mean ESs on both sides were identified through visual inspection of the forest plots. Furthermore, outliers were kept if deleting them did not significantly impact the results. A narrative synthesis was also conducted for those trials and outcomes that were excluded from the meta-analyses to give a comprehensive picture of the data and to make comparisons between different CBIs.

## Results

### Study Selection

**Fig. 1:**
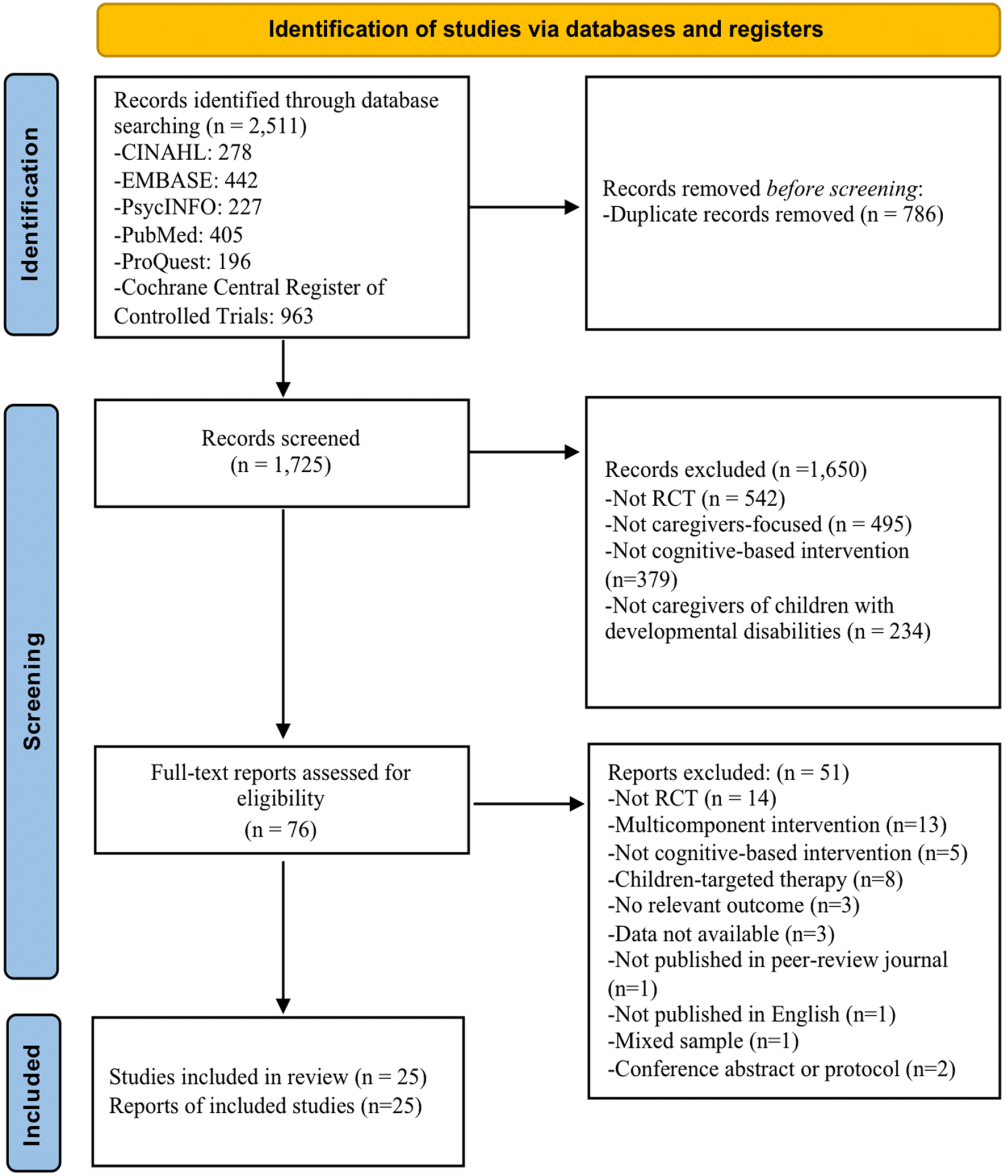
PRISMA Flow Diagram of Study Selection

A total of 2511 records were identified. After excluding 786 duplicates, the titles and abstracts of 1725 articles remained for the initial screening, and 76 eligible articles were retained for further full-text screening. No extra articles were identified from the search engines or reference lists. Finally, a total of 25 RCTs were selected for inclusion in this review. Figure 1 depicts the PRISMA flow chart of the procedure and the results of the article search and selection. Supplementary Table 8 lists all excluded articles following full-text screening.

### Study Characteristics

Supplementary Table 10 summarizes the main characteristics of the 25 included RCTs. Studies were published between 2006 and 2023, and the majority of studies were developed in the United States (38.10%, n = 8) (Chronis et al., 2006; Dykens et al., 2014; Feinberg et al., 2014; Ferraioli & Harris, 2013; Hahs et al., 2019; Kuhlthau et al., 2020; Neece, 2014; Schwartzman et al., 2022). Other research locations included China (n = 4; (Ho et al., 2021; Liu et al., 2021; Lo et al., 2017a, 2017b; Lo et al., 2017a, 2017b)), Australia (n = 2; Whittingham et al., 2022; Wong et al., 2010)), Iran (n = 4; Behbahani et al., 2018; Khoshvaght et al., 2021; Shareh & Yazdanian, 2023; Sharif et al., 2015)), Spain (n = 2; Lobato et al., 2023; Valero et al., 2022), Italy (Marino et al., 2021), the Netherlands (Siebelink et al., 2018), Nigeria (Onyishi et al., 2023), Turkey (Çiçek Gümüş & Öncel, 2022), and India (Pandya, 2021). All of the studies were two-arm RCTs with a parallel control design, and the most of studies were pilot RCTs (36%, n = 9) (Ferraioli & Harris, 2013; Hahs et al., 2019; Ho et al., 2021; Kuhlthau et al., 2020; Lobato et al., 2023; Marino et al., 2021; Neece, 2014; Schwartzman et al., 2022; Valero et al., 2022).

*Characteristics of Participants* The 25 RCTs included 1,915 participants (n_treatment_ = 970, n_control_ = 945) with sample sizes ranging from 14 (Lobato et al., 2023) to 243 (Dykens et al., 2014). The mean age of the parents ranged from 33.5 (standard deviation [SD]: 7.00) (Feinberg et al., 2014) to 47 (range: 37–60) (Wong et al., 2010). The majority of the participants (85.15%) were female, and eight studies included mothers only. The mean age of children with DD varied from 2.83 (SD: 11.00) (Feinberg et al., 2014) to 13.00 (range: 10–16) (Ho et al., 2021), with boys accounting for the majority (70.72%). Four types of DD were identified among the children in the included studies: ASD (n = 10, 40%), ADHD (n = 8, 32%), CP (n = 2, 8%), ID (n = 1, 4%), and blended DD (n = 4, 16%).

*Characteristics of Interventions* The following five main approaches were classified: (1) CBT (n = 6 Chronis et al., 2006; Feinberg et al., 2014; Onyishi et al., 2023; Schwartzman et al., 2022; Sharif et al., 2015; Wong et al., 2010)); (2) MBIs (n = 12; (Behbahani et al., 2018; Dykens et al., 2014; Ferraioli & Harris, 2013; Ho et al., 2021; Kuhlthau et al., 2020; Liu et al., 2021; Lo et al., 2017a, 2017b; Lo et al., 2017a, 2017b; C. L. Neece, 2014; Pandya, 2021; Siebelink et al., 2018; Valero et al., 2022)); (3) ACT (n = 5; Çiçek Gümüş & Öncel, 2022; Hahs et al., 2019; Lobato et al., 2023; Marino et al., 2021; Whittingham et al., 2022)); (4) DBT (n = 1; (Shareh & Yazdanian, 2023); and (5) CFT (n = 1; (Khoshvaght et al., 2021)).

**Fig. 2:**
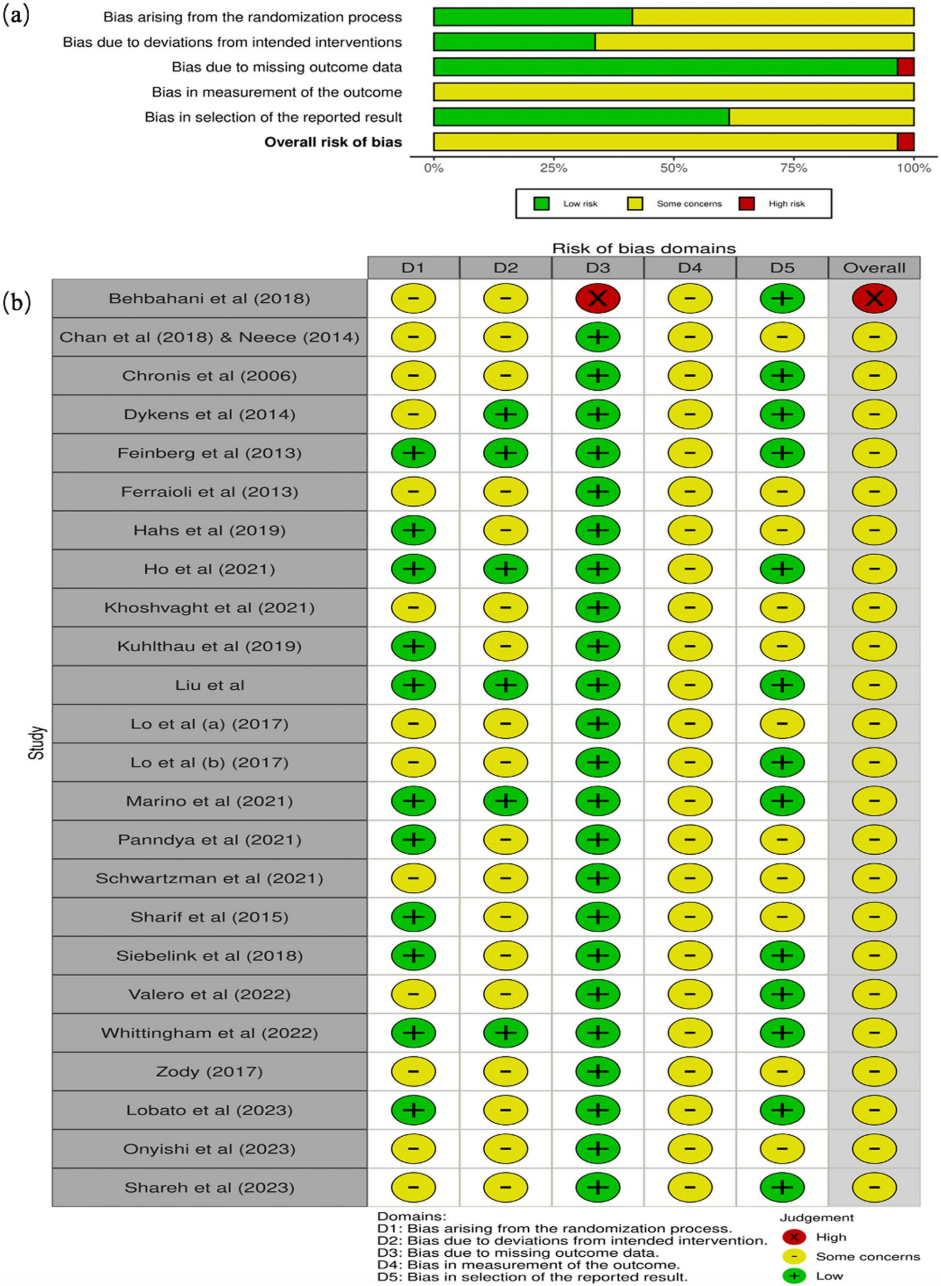
**a** Quality assessment graph about each risk of bias item presented as percentages across all included studies; **b** Summary of risk of bias assessment of the included randomized controlled trails by Cochrane Collaboration risk of bias tool

Across the included studies, the duration of CBIs ranged from 2 days (Hahs et al., 2019) to 50 weeks (Pandya, 2021) (mean = 70.08 days, SD = 64.98, median = 56). The average number of CBI sessions ranged from 2 to (Hahs et al., 2019) to 24 sessions (mean = 8.4, SD = 4.05, median = 8), except for a study with posted messages (Pandya, 2021). Seven studies adopted the format of a parent‒child dyad (Behbahani et al., 2018; Çiçek Gümüş & Öncel, 2022; Ho et al., 2021; Lo et al., 2017a, 2017b; Neece, 2014; Siebelink et al., 2018; Valero et al., 2022), and the remaining studies all only involved parents as active participants. Two modes of intervention delivery were identified: face-to-face (n = 21) and online (n = 3), and one study did not report the delivery mode. Moreover, four studies delivered the intervention to individuals, while others delivered the interventions to groups of participants (n = 21; 84%). The majority of the investigations disclosed their settings (n = 18), which included in the community (n = 3), hospitals/clinics (n = 6), service/rehabilitation centres (n = 4), schools (n = 2), and internet platforms (n = 3) (Fig. 2).

*Characteristics of Control Groups* The majority of studies (n = 21) used inactive controls, such as waitlists (n = 13), no treatments (n = 4), and treatments as usual (TAU; n = 4), while the others used active controls, such as child-targeted parenting skills training (n = 2) (Ferraioli & Harris, 2013; Marino et al., 2021), a 1-day mindfulness workshop (Lo et al., 2017a, 2017b), and positive psychotherapy (Dykens et al., 2014).

*Outcome Measures* Table 1 presents a list of all measurement instruments, all of which were valid and reliable questionnaires. Studies used various time-points to obtain their results. One study additionally performed an evaluation at the midpoint of the intervention (Dykens et al., 2014). Over half of the studies (n = 15) used multiple time-points to assess longer-term effects, including short- (less than 3 months) (Behbahani et al., 2018; Khoshvaght et al., 2021; Schwartzman et al., 2022; Sharif et al., 2015), medium- (3 to 5 months) (Chronis et al., 2006; Çiçek Gümüş & Öncel, 2022; Lobato et al., 2023; Onyishi et al., 2023) and long-term effects (6 months or more) (Dykens et al., 2014; Ferraioli & Harris, 2013; Neece, 2014; Siebelink et al., 2018; Valero et al., 2022; Whittingham et al., 2022; Wong et al., 2010).

**Table 1:** Measurement instruments used in the cognitive-based interventions

Outcomes	Measurements	References
Stress (n_number of RCTs_ = 20)	Parenting Stress Index (PSI; Abidin, (1983)) (n = 3)	Dykens et al., (2014); Ho et al., (2021); Wong et al., (2010)
	Parenting Stress Index: Short Form (PSI-SF; Abidin, (1990)) (n = 10)	Behbahani et al., (2018); Feinberg et al., (2014); Ferraioli and Harris, (2013); Liu et al., (2021); Lo et al., (2017a, 2017b); Marino et al., (2021); Neece, (2014); Pandya, (2021); Valero et al., (2022)
	Perceived Stress Scale (PSS; Cohen et al., (1983)) (n = 2)	Chronis et al., (2006); Lobato et al., (2023)
	Depression Anxiety Stress Scale—21-Stress Subscale (DASS-21; Lovibond & Lovibond, (1995)) (n = 5)	Çiçek Gümüş & Öncel, (2022); Onyishi et al., (2023); Schwartzman et al., (2022); Sharif et al., (2015); Whittingham et al., (2022)
Anxiety (n = 10)	Beck Anxiety Inventory (BAI; Aaron T Beck & Steer, (1990)) (n = 3)	Chronis et al., (2006); Dykens et al., (2014); Khoshvaght et al., (2021)
	Depression Anxiety Stress Scale—21-Anxiety Subscale Lovibond & Lovibond, (1995) (n = 5)	Çiçek Gümüş & Öncel, (2022); Onyishi et al., (2023); Schwartzman et al., (2022); Sharif et al., (2015); Whittingham et al., (2022)
	Hamilton Anxiety Scale (HAMA; Hamilton, (1959)) (n = 1)	Liu et al., (2021)
	The Patient Health Questionnaire‑4 (PHQ-4; Kroenke et al., (2009)) (n = 1)	Kuhlthau et al., (2020)
Depression (n = 15)	Center for Epidemiological Studies Depression Scale (CES-D; Radloff, (1977)) (n = 2)	Lo et al., (2017a, b); Neece, (2014)
	Beck Depression Inventory (BDI; Beck et al., (1961)) (n = 2)	(Chronis et al., 2006; Khoshvaght et al., 2021)
	Beck Depression Inventory-II (BDI-II; Beck et al., (1996)) (n = 3)	(Dykens et al., 2014; Hahs et al., 2019; Shareh & Yazdanian, 2023)
	Depression Anxiety Stress Scale—21-Depression Subscale (Lovibond & Lovibond, 1995) (n = 5)	Çiçek Gümüş & Öncel, (2022); Onyishi et al., (2023); Schwartzman et al., (2022); Sharif et al., (2015); Whittingham et al., (2022)
	Quick Inventory of Depressive Symptomatology (QIDS; Rush et al., (2003)) (n = 1)	Feinberg et al., (2014)
	Hamilton Depression Scale (HAMA; Hamilton, (1960) (n = 1)	Liu et al., (2021)
	The Patient Health Questionnaire‑4 Kroenke et al., (2009) (n = 1)	Kuhlthau et al., (2020)
Distress (n = 14)	Visual Analogue Scale (VAS)–Distress Lesage et al., (2012) (n = 1)	Kuhlthau et al., (2020)
	Parenting Stress Index—Parental Distress Subscale Abidin, (1990) (n = 1)	Ho et al., (2021)
	Parenting Stress Index: Short Form—Parental Distress Subscale Abidin, (1990) (n = 9)	Behbahani et al., (2018); Dykens et al., (2014); Liu et al., (2021); Lo et al., (2017a, b); Marino et al., (2021); Pandya, (2021); Schwartzman et al., (2022); Valero et al., (2022)
	Depression Anxiety Stress Scale -21 Lovibond & Lovibond, (1995) (n = 3)	Çiçek Gümüş & Öncel, (2022); Shareh & Yazdanian, (2023); Siebelink et al., (2018)
Parental well-being (n = 9)	Ryff Scales of Psychological Well-Being—Short Form (Ryff & Keyes, (1995)) (n = 1)	Dykens et al., (2014)
	General Health Questionnaire (GHQ; Goldberg & Williams, (1988)) (n = 1)	Sharif et al., (2015)
	General Health Questionnaire—12 (GHQ-12; Goldberg & Williams, (1988)); (n = 2)	Lobato et al., (2023; Wong et al., (2010)
	General Health Questionnaire—28 (GHQ-28; Goldberg, (1978)) (n = 1)	Ferraioli & Harris, (2013)
	World Health Organization‑5 Well‑being Index (WHO-5; Bech et al., (2003)) (n = 3)	Ho et al., (2021); Lo et al., (2017a, b); Siebelink et al., (2018)
	Personal Wellbeing Index (PWI; Lau et al., (2005)) (n = 1)	Whittingham et al., (2022)
Quality of relationship (parent–child) (n = 10)	Parenting Stress Index—Parent–child Dysfunctional Interaction Subscale Abidin, (1990) (n = 1)	Ho et al., (2021)
	Parenting Stress Index: Short Form—Parent–child Dysfunctional Interaction Subscale Abidin, (1990) (n = 9)	Behbahani et al., (2018); Chronis et al., (2006); Liu et al., (2021); Lo et al., (2017a, (b); Marino et al., (2021); Pandya, (2021); Schwartzman et al., (2022); Valero et al., (2022)
Psychological flexibility (n = 6)	Acceptance and Action Questionnaire—Second version (AAQ-II; Bond & Bunce, (2003)) (n = 5)	Çiçek Gümüş & Öncel, (2022); Hahs et al., (2019); Marino et al., (2021); Schwartzman et al., (2022); Whittingham et al., (2022)
	Parental Acceptance Questionnaire (6-PAQ; Greene et al., (2015)) (n = 1)	Lobato et al., (2023)
Mindful parenting (n = 4)	The Interpersonal Mindfulness in Parenting (IMP; Duncan et al., (2009))	Lo et al., (2017a, b); Siebelink et al., (2018); Whittingham et al., (2022)
Mindful awareness (n = 4)	Mindful Attention Awareness Scale (MAAS; Brown et al., (2003)) (n = 4)	Hahs et al., (2019); Liu et al., (2021); Marino et al., (2021); Schwartzman et al., (2022)

### Meta-analyses of CBI Outcomes

The meta-analyses comprised 21 studies in total. Two MBI studies developed by Dykens et al. (2014) and Neece (2014) were excluded from the meta-analyses because the mean and/or SD values could not be obtained from the report. Another MBI study, Pandya (2021), which revealed extraordinarily large ESs (Hedges’ g range: 4.93–11.2) for all the long-term results (50 weeks), was considered an outlier for several outcomes (e.g., parental stress, distress, and parent‒child relationship). Similar to Pandya (2021), the outcomes reported by Çiçek Gümüş and Öncel (2022) were also identified as an outlier, with large ESs ranging from 2.706 to 6.084. The results of the meta-analyses are listed in Table 2.

**Table 2:** Summary of findings

Outcomes	Meta-analyses			Narrative syntheses			Certainty of the evidence
	No. of participants	SMD (95%CI)	References	No. of participants	Hedges’ g	References	
Parental stress	1015	− 0.69 (− 1.05, − 0.33)	Behbahani et al., (2018); Chronis et al., (2006); Feinberg et al., (2014); Ferraioli and Harris, (2013); Ho et al., (2021); Liu et al., (2021); Lo et al., (2017a, 2017b); Marino et al., (2021); Onyishi et al., (2023); Schwartzman et al., (2022); Sharif et al., (2015); Valero et al., (2022); Whittingham et al., (2022); Wong et al., (2010)	137 60 46	− 8.64** − 5.81** − 0.70*	Pandya, (2021) Çiçek Gümüş and Öncel, (2022) Neece, (2014)	Moderate
Depressive symptoms	925	− 0.95 (− 1.47, − 0.43)	Chronis et al., (2006); Feinberg et al., (2014); Hahs et al., (2019); Khoshvaght et al., (2021); Kuhlthau et al., (2020); Liu et al., (2021); Lo et al., (2017a, 2017b); Onyishi et al., (2023); Schwartzman et al., (2022); Shareh and Yazdanian, (2023); Sharif et al., (2015); Whittingham et al., (2022)	243 46 60	− 1.05** − 0.87* − 5.04**	Dykens et al., (2014) Neece, (2014) Çiçek Gümüş and Öncel, (2022)	High
Anxiety	484	− 0.78 (− 1.39, − 0.18)	Chronis et al., (2006); Khoshvaght et al., (2021); Kuhlthau et al., (2020); Liu et al., (2021); Onyishi et al., (2023); Schwartzman et al., (2022); Sharif et al., (2015); Whittingham et al., (2022)	243 60	− 0.90** − 2.71**	Dykens et al., (2014) Çiçek Gümüş and Öncel, (2022)	Moderate
Parental distress	838	− 0.29 (− 0.42, − 0.15)	Behbahani et al., (2018); Ho et al., (2021); Kuhlthau et al., (2020); Liu et al., (2021); Lo et al., (2017a, 2017b); Marino et al., (2021); Schwartzman et al., (2022); Shareh and Yazdanian, (2023); Siebelink et al., (2018); Valero et al., (2022)	137 46 60	− 6.7** − 0.70* − 5.50**	Pandya, (2021) Neece, (2014) Çiçek Gümüş and Öncel, (2022)	High
Parental well-being	426	0.62 (0.20, 1.03)	(Ferraioli and Harris, (2013); Ho et al., (2021); Lo et al., (2017a, 2017b); Lobato et al., (2023); Sharif et al., (2015); Siebelink et al., (2018); Whittingham et al., (2022); Wong et al., (2010)	–	–	–	Moderate
Parent–child relationship	615	0.43 (0.22, 0.64)	Behbahani et al., (2018); Chronis et al., (2006); Ho et al., (2021); Liu et al., (2021); Lo et al., (2017a, 2017b); Lo et al., (2017a, 2017b); Marino et al., (2021); Schwartzman et al., (2022); Valero et al., (2022)	137	5.49**	Pandya, (2021)	High
Mindful parenting	434	0.15 (− 0.05, 0.35)	Lo et al., (2017a, 2017b); Siebelink et al., (2018); Whittingham et al., (2022)	–	–	–	Moderate
Mindful awareness	185	1.99 (− 0.64, 4.62)	Hahs et al., (2019); Liu et al., (2021); Marino et al., (2021); Schwartzman et al., (2022)	–	–	–	Very low
Psychological flexibility	138	1.47 (− 0.42, 3.36)	Hahs et al., (2019); Lobato et al., (2023); Marino et al., (2021); Schwartzman et al., (2022); Whittingham et al., (2022)	60	6.08**	Çiçek Gümüş and Öncel, (2022)	Very low

*p < 0.05; **p < 0.001

**Fig. 3:**
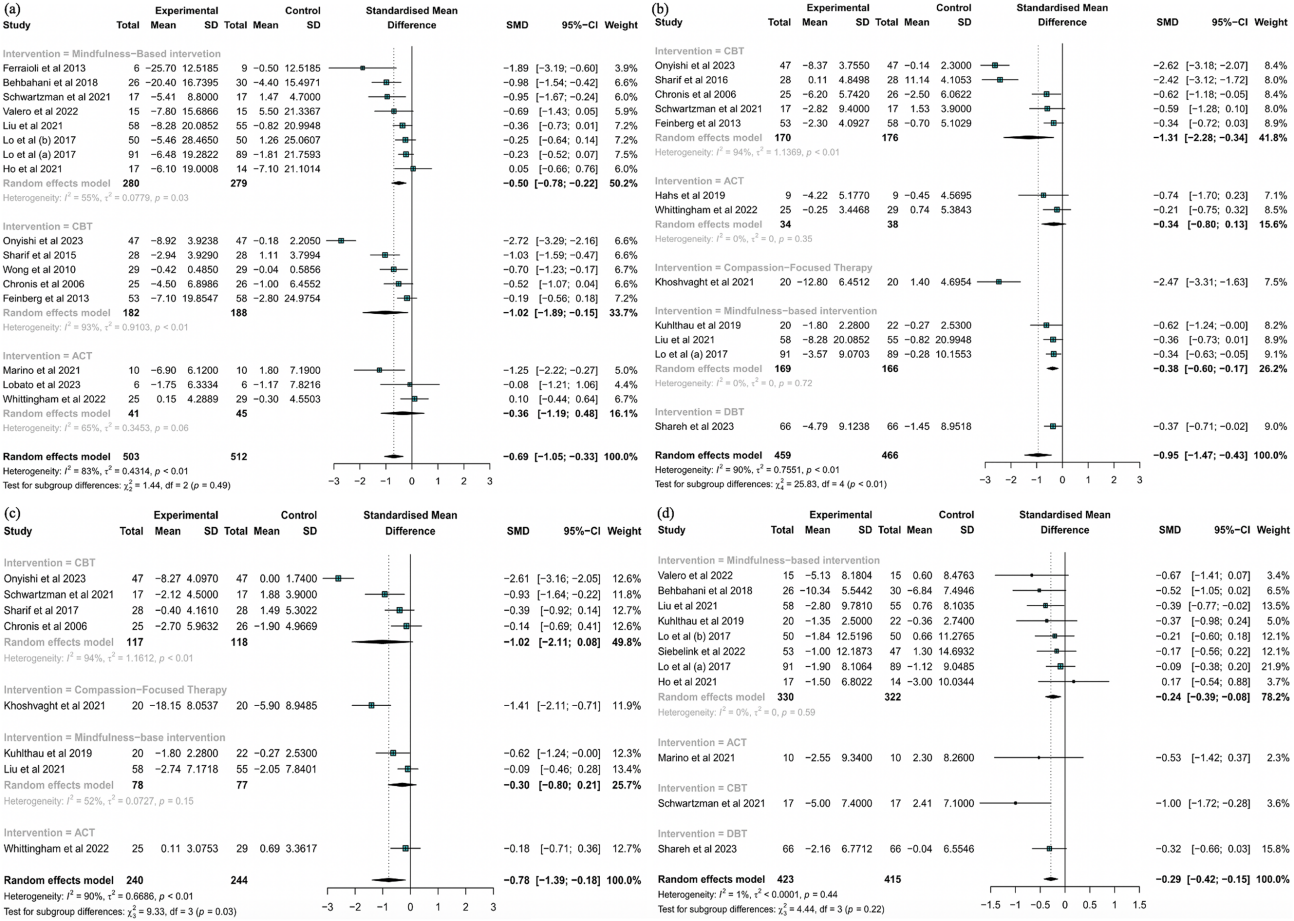
Forest plots effect of CBIs on **a** parental stress; **b** depressive symptoms of parents; **c** anxiety of parents; **d** parental distress

*Parental Stress* Sixteen trials with 1015 participants were included to assess the effectiveness of CBIs on parental stress (Behbahani et al., 2018; Chronis et al., 2006; Feinberg et al., 2014; Ferraioli & Harris, 2013; Ho et al., 2021; Liu et al., 2021; Lo et al., 2017a, 2017b; Lo et al., 2017a, 2017b; Marino et al., 2021; Onyishi et al., 2023; Schwartzman et al., 2022; Sharif et al., 2015; Valero et al., 2022; Whittingham et al., 2022; Wong et al., 2010). The pooled results showed that CBIs had a significant effect on lessening parental stress when compared with the control group (g = − 0.69, 95% CI [− 1.05, -0.33], P < 0.01, I^2^ = 83%; Fig. 3a). Additionally, the robustness of the finding was demonstrated by the fact that the pooled results remained unchanged after performing a sensitivity analysis (Supplementary Fig. 1). No publication bias (P = 0.1255) was identified by using Egger’s test and trim-and-fill funnel plot (Supplementary Fig. 2).

*Depressive Symptoms* A total of 12 RCTs including 925 subjects were included to analyse the effects of CBIs on depressive symptoms (Chronis et al., 2006; Feinberg et al., 2014; Hahs et al., 2019; Khoshvaght et al., 2021; Kuhlthau et al., 2020; Liu et al., 2021; Lo et al., 2017a, 2017b; Onyishi et al., 2023; Schwartzman et al., 2022; Shareh & Yazdanian, 2023; Sharif et al., 2015; Whittingham et al., 2022). The results indicated a large effect of CBIs on alleviating depressive symptoms compared to the control group (g = − 0.95, 95% CI [− 1.47, − 0.43], P < 0.01, I^2^ = 90%; Fig. 3b). The robustness of finding was confirmed by sensitivity analysis and trim-and-fill funnel plot, despite Egger’s test revealed potential publication bias (p = 0.0467) (Supplementary Figs. 3, 4).

*Anxiety* Anxiety was assessed by eight trials with 484 participants (Chronis et al., 2006; Khoshvaght et al., 2021; Kuhlthau et al., 2020; Liu et al., 2021; Onyishi et al., 2023; Schwartzman et al., 2022; Sharif et al., 2015; Whittingham et al., 2022). Compared with the control group, the CBIs identified a significant reduction in anxiety (g = − 0.78, 95% CI [− 1.39, − 0.18], P < 0.01, I^2^ = 90%; Fig. 3c). The sensitivity analysis confirmed the robustness of the results (Supplementary Fig. 5).

*Parental Distress* The effectiveness of CBIs on parental distress was evaluated in 11 trials with 838 participants (Behbahani et al., 2018; Ho et al., 2021; Kuhlthau et al., 2020; Liu et al., 2021; Lo et al., 2017a, 2017b; Lo et al., 2017a, 2017b; Marino et al., 2021; Schwartzman et al., 2022; Shareh & Yazdanian, 2023; Siebelink et al., 2018; Valero et al., 2022). The results revealed that, in comparison with the control group, parental distress was significantly reduced (g = − 0.29, 95% CI [− 0.42, − 0.15], P = 0.44, I^2^ = 1%; Fig. 3e). Furthermore, following a sensitivity analysis in which studies were removed one by one, the pooled results remained constant, confirming the robustness of the finding (Supplementary Fig. 6). The Egger’s test (P = 0.1042) and funnel plot also revealed no evidence of potential publication bias (Supplementary Fig. 7).

**Fig. 4:**
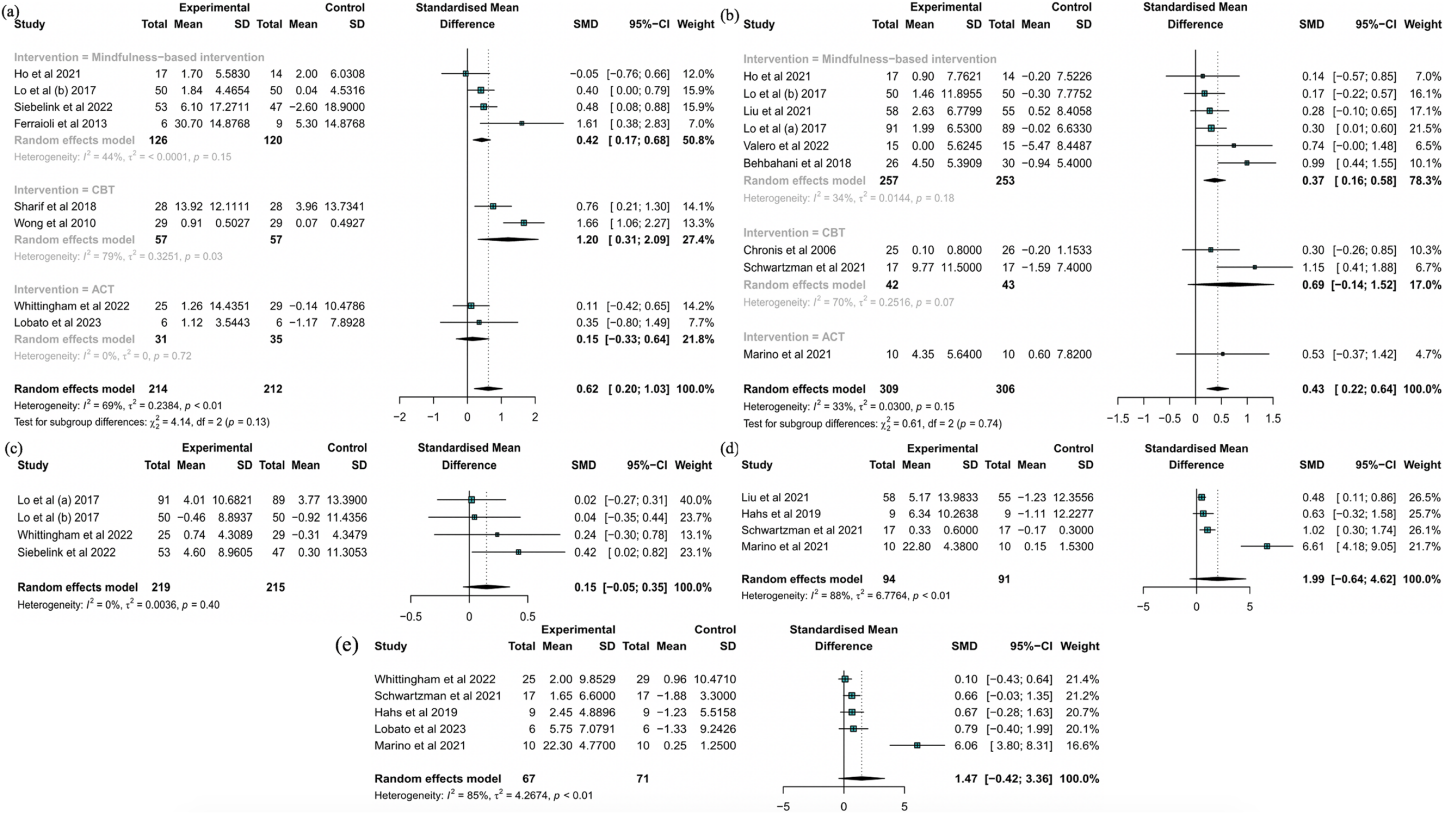
Forest plots effect of CBIs on **a** subjective well-being; **b** parent–child relationship; **c** mindful parenting; **d** mindful awareness; **e** psychological flexibility

*Parental Well-being* Eight RCTs involving 426 subjects were used to examine the effects of CBIs on parental well-being (Ferraioli & Harris, 2013; Ho et al., 2021; Lo et al., 2017a, 2017b; Lobato et al., 2023; Sharif et al., 2015; Siebelink et al., 2018; Whittingham et al., 2022; Wong et al., 2010). The pooled results showed that CBIs had a medium effect on enhancing parental well-being (g = 0.62, 95% CI [0.20, 1.03], P < 0.01, I^2^ = 69%; Fig. 4a). The sensitivity analysis confirmed the robustness of the result (Supplementary Fig. 8).

*Parent‒Child Relationship* The parent‒child relationship was examined by nine studies with 615 participants (Behbahani et al., 2018; Chronis et al., 2006; Ho et al., 2021; Liu et al., 2021; Lo et al., 2017a, 2017b; Lo et al., 2017a, 2017b; Marino et al., 2021; Schwartzman et al., 2022; Valero et al., 2022). CBIs had a small effect on the parent‒child relationship (g = 0.43, 95% CI [0.22, 0.64], P = 0.15, I^2^ = 33%; Fig. 4b). Furthermore, the result was not altered after performing a sensitivity analysis (Supplementary Fig. 9).

*Mindful Parenting* Four trials involving 434 participants reported the effects of CBIs on mindful parenting (Lo et al., 2017a, 2017b; Lo et al., 2017a, 2017b; Siebelink et al., 2018; Whittingham et al., 2022). The findings demonstrated that CBIs did not substantially promote mindful parenting (g = 0.15, 95% CI [− 0.05, 0.35], P = 0.40, I^2^ = 0%; Fig. 4c). The sensitivity analysis confirmed that these findings were reliable (Supplementary Fig. 10).

*Mindful Awareness* Four trials with 185 subjects were used to investigate the influence of CBIs on mindful awareness (Hahs et al., 2019; Liu et al., 2021; Marino et al., 2021; Schwartzman et al., 2022). No significant improvements were revealed in the pooled results (g = 1.99, 95% CI [− 0.64, 4.62], P < 0.01, I^2^ = 88%; Fig. 4d); however, the results of the sensitivity analysis revealed the potential benefit of CBIs after removing an outlier (g = 0.61, 95% CI [0.27, 0.96], P < 0.01, I^2^ = 0%; Supplementary Fig. 11) (Marino et al., 2021).

*Psychological Flexibility* A total of five studies including 138 participants were used to examine the effect of CBIs on psychological flexibility (Hahs et al., 2019; Lobato et al., 2023; Marino et al., 2021; Schwartzman et al., 2022; Whittingham et al., 2022). The results of the meta-analysis revealed that the CBIs that were implemented in those studies did not significantly enhance psychological flexibility (g = 1.47, 95% CI [− 0.42, 3.36], P < 0.01, I^2^ = 85%; Fig. 4e). However, the favourable effect of CBIs was discovered after excluding one outlier (g = 0.43, 95% CI [0.03, 0.83], P = 0.14, I^2^ = 0%; Supplementary Fig. 12) (Marino et al., 2021).

### Comparison of Treatment Effects Between Different Intervention Approaches

This subgroup analysis did not include mindful awareness, or psychological flexibility due to the paucity of relevant research. The subgroup analysis indicated that MBIs had a significant effect on parental stress levels (k_number of outcomes_ = 8, g = − 0.50, 95% CI [− 0.78, − 0.22], P = 0.03, I^2^ = 55%), depressive symptoms (k = 3, g = − 0.38, 95% CI [− 0.60, − 0.17], P = 0.72, I^2^ = 0%), parental distress levels (k = 8, g = − 0.24, 95% CI [− 0.39, − 0.08], P = 0.59, I^2^ = 0%), parental well-being levels (k = 4, g = 0.42, 95% CI [0.17, 0.68], P = 0.15, I^2^ = 44%), and parent‒child relationships (k = 6, g = 0.37, 95% CI [0.16, 0.58], P = 0.18, I^2^ = 34%). And CBT could also effectively decrease these parents’ parental stress levels (k = 5, g = − 1.02, 95% CI [− 1.89, − 0.15], P < 0.01, I^2^ = 93%) and depressive symptoms (k = 5, g = − 1.31, 95% CI [− 2.28, − 0.34], P < 0.01, I^2^ = 94%), while improving parental well-being (k = 2, g = 1.20, 95% CI [0.31, 2.09], P = 0.03, I^2^ = 79%). The results of subgroup analysis for different interventions are displayed in Figs.3 and 4.

### Comparison of Treatment Effects Between Parent-Only and Parent‒Child Dyad CBIs

A subgroup analysis revealed that parent‒child dyad CBIs were more favourable than parent-only CBIs in improving parent‒child relationships (g = 0.66, 95% CI [0.15, 1.16] vs. 0.33, 95% CI [0.15, 0.51]; Supplementary Fig. 15), while parent-only CBIs were more beneficial than parent‒child dyad CBIs in decreasing parental stress levels (g = − 0.73, 95% CI [− 1.16, − 0.30] vs. − 0.57 [− 1.18, 0.04]; Supplementary Fig. 13), distress levels (g = − 0.29, 95% CI [− 0.51, − 0.10] vs. − 0.28, 95% CI [− 0.55, − 0.00]; Supplementary Fig. 14), and enhancing parental well-being (g = 0.76, 95% CI [0.24, 1.29] vs. 0.30, 95% CI [− 0.19, 1.03]; Supplementary Fig. 16).

### Comparison of Treatment Effects Between Intervention Durations

According to the subgroup analyses, the ideal intervention duration was 1 to 8 weeks for decreases in parental stress levels (k = 7, g = − 0.60, 95% CI [− 0.95, − 0.25], P = 0.03, I^2^ = 58%; Supplementary Fig. 17), depressive symptoms (k = 5, g = − 0.91, 95% CI [− 1.63, − 0.18], P < 0.01, I^2^ = 82%; Supplementary Fig. 18), anxiety levels (k = 3, g = − 0.97, 95% CI [− 1.43, − 0.51], P = 0.25, I^2^ = 27%; Supplementary Fig. 19), and parental distress levels (k = 7, g = − 0.30, 95% CI [− 0.49, − 0.11], P = 0.25, I^2^ = 23%; Supplementary Fig. 20), as well as for improvements in parental well-being levels (k = 4, g = 0.49, 95% CI [0.22, 0.75], P = 0.33, I^2^ = 13%; Supplementary Fig. 21), parent‒child relationships (k = 5, g = 0.59, 95% CI [0.21, 0.97], P = 0.03, I^2^ = 62%; Supplementary Fig. 22), mindful awareness (k = 2, g = 0.88, 95% CI [0.31, 1.45], P = 0.52, I^2^ = 0%; Supplementary Fig. 23), and parental psychological flexibility (k = 3, g = 0.69, 95% CI [0.18, 1.19], P = 0.98, I^2^ = 0%; Supplementary Fig. 24). However, significant effects on parental stress levels (k = 8, g = − 0.43, 95% CI [− 0.72, − 0.15], P = 0.03, I^2^ = 54%; Supplementary Fig. 17) and parent‒child relationships (k = 4, g = 0.28, 95% CI [0.01, 0.55], P = 0.93, I^2^ = 0%; Supplementary Fig. 22) were also observed when the intervention duration was over eight weeks.

### Comparison of Treatment Effects Between Two Types of DD in Children

No subgroup analyses were conducted for children with CP, ID, or blended types of DD due to the limited number of relevant studies. The subgroup analysis found that CBIs had significantly positive effects on parental stress levels (k = 7, g = − 0.60, 95% CI [− 0.84, − 0.36], P = 0.18, I^2^ = 32%; Supplementary Fig. 25), parental distress levels (k = 5, g = − 0.33, 95% CI [− 0.52, − 0.13], P = 0.66, I^2^ = 0%; Supplementary Fig. 28), parental well-being levels (k = 4, g = 0.79, 95% CI [0.25, 1.33], P < 0.01, I^2^ = 77%; Supplementary Fig. 29), and parent‒child relationships (k = 5, g = 0.43, 95% CI [0.14, 0.73], P = 0.14, I^2^ = 43%; Supplementary Fig. 30) for parents of children with ADHD. In addition, CBIs were shown to effectively reduce parental stress levels (k = 7, g = − 0.99, 95% CI [− 1.79, − 0.20], P < 0.01, I^2^ = 91%; Supplementary Fig. 25), depressive symptoms (k = 5, g = − 0.99, 95% CI [− 1.83, − 0.15], P < 0.01, I^2^ = 91%; Supplementary Fig. 26), and anxiety levels (k = 3, g = − 1.40, 95% CI [− 2.62, − 0.17], P < 0.01, I^2^ = 92%; Supplementary Fig. 27) for parents of children with ASD.

### Narrative Syntheses

Four studies (Dykens et al., 2014; Pandya, 2021) (Çiçek Gümüş & Öncel, 2022; Neece, 2014) conducted narrative analyses. When compared to the positive psychotherapy group, Dykens et al. (2014) observed substantial postintervention reductions in depression (g = 1.05) and anxiety (g = 0.90) symptoms for parents of children with DD using an in-person MBI. Moreover, Pandya (2021) reported that utilizing online MBI versus a waitlist control group resulted in a considerable reduction in parental stress (g = − 8.64) distress (g = − 6.70) levels, and a postintervention improvement in parent‒child relationships (g = 5.63). Furthermore, Neece (2014) identified that mindfulness-based stress reduction could significantly alleviate parental distress (Cohens’ d = 0.70) and depression (d = 0.87) for parents of children with DD postintervention compared to the wait-list control group. In addition, Çiçek Gümüş and Öncel (2022) found significant reductions in parental stress (g = − 5.81), depressive symptoms (g = − 5.04), anxiety (g = − 2.71), parental distress (g = − 5.50), and psychological flexibility (g = − 6.08) for parents of children with ASD and mental disorder(s) postintervention when comparing ACT to the TAU control group.

### Risk of Bias

In general, one study was determined to have a high risk of bias (Behbahani et al., 2018), while the other studies were judged to have some concerning aspects regarding the risk of bias (Figure 2). Due to a lack of information on allocation concealment, 13 studies (52%) were found to have some concerning aspects regarding a risk of bias resulting from the randomization technique, whereas the other studies showed low risk. A total of 18 studies (72%) were revealed to have some concerning aspects regarding deviations from the intended intervention, while the remaining seven studies were deemed to be low risk. Except for one study, which was deemed high risk due to no information concerning insufficient/missing data, other studies were deemed low risk (96%). All studies were noted to have some concerning aspects regarding outcome measurements because they all used self-reported instruments. In the selection of the published results, 13 studies (52%) were rated as low risk, while the remaining studies were evaluated as having some concerning aspects due to inadequate information regarding prespecified analytical plans/protocols.

### Quality of the Evidence

The aggregate GRADE evaluation of the outcomes revealed that the certainty of evidence results ranged from “high” to “very low” (Table 1). The effects of CBIs on parental distress, depression levels, and parent‒child relationships were determined to have a high certainty of evidence. The evidence regarding effects on parental stress and anxiety levels, parental well-being, and mindful parenting was judged as having moderate certainty of evidence because of significant heterogeneity or inconsistency. The evidence regarding effects on mindful awareness and psychological flexibility had a low level of certainty, mainly because of the risk of bias, inconsistency (high heterogeneity), and/or imprecision (sample size lower than 400) of the relevant studies.

## Discussion

This review and meta-analysis summarized and synthesized 25 RCTs to examine the effectiveness of CBIs in reducing distress levels and improving mental health and wellbeing among parents of children with DD. The results of the meta-analysis highlight that CBIs may significantly alleviate the levels of parental stress, depressive symptoms, anxiety, and parental distress and improve parental well-being and parent‒child relationships for parents of children with DD. Moreover, MBIs and CBT interventions showed positive effects and were recommended as potential optimal approaches in the subgroup analyses. The findings of our review echo those of previous systematic reviews and meta-analyses that CBIs had positive effects for reducing parental stress and psychological symptoms postintervention in parents of children with DD. Osborn et al., (2021) explored the impact of mindfulness therapies on psychological distress in parents of children with DD and reported small to large ESs (g range: 0.39 to 1.94). Lee et al. (2022) examined the effect of mindfulness parent training on parenting stress levels and found small to large ESs (g range: 0.06 to 0.84) for parents of children with ADHD. Hartley et al. (2019) conducted a meta-analysis on MBIs for parents of children with ASD and revealed postintervention improvement in subjective well-being. Bourke-Taylor et al. (2021) performed a meta-analysis on group interventions for mothers of children with disabilities and found that CBT demonstrated significantly large postintervention ESs for parenting stress levels (g = 0.86) and mental health (g = 1.14). As MBIs and CBT interventions have been identified as optimal approaches, future well-designed RCTs are needed to confirm their effects on parents of children with DD and, equally importantly, the factors and mechanisms of action impacting their efficacy.

However, this review did not identify significant improvement in the outcomes of mindfulness parenting, mindfulness awareness, and psychological flexibility postintervention for parents of children with DD, which is in line with Chua and Shorey’s (2022) meta-analysis, which examined the effectiveness of MBIs and ACT interventions among parents of children with DD. The findings of the current study suggest that cautious interpretation is needed due to the limited number of studies and the small sample sizes included in the meta-analysis, as well as the heterogeneity of the intervention content. Another reason for the resulting ineffectiveness is that those studies did not set mindfulness parenting, mindfulness awareness, and/or psychological flexibility as primary outcomes. The intervention may only indirectly impact those outcomes, implying that some outcomes need to be sufficiently addressed with current CBIs. In contrast, Rayan and Ahmad (2018) found that five out of six trials reported postintervention improvements in mindful parenting among parents of children with disabilities, although only a narrative synthesis was performed in that study and only one RCT was included, implying that direct comparisons could not be conducted. Similarly, studies included in our study indicated that CBIs could facilitate mindful awareness and psychological flexibility outcomes, but the opposite pooled result was obtained after performing meta-analyses. This may be because we applied the random-effects model to account for the significant heterogeneity since one study (Marino et al., 2021) displayed an extremely large ES for these two outcomes. The positive effect appeared for mindful awareness (g = 0.70, 95% CI [0.39, 1.01], P < 0.01, I^2^ = 88%) and psychological flexibility (g = 0.56, 95% CI [0.19, 0.92], P < 0.01, I^2^ = 85%) after we switched to the fixed-effects model. Therefore, additional studies are required to further explore the potential benefits of CBIs on these inconsistent outcomes.

Moreover, Chua and Shorey’s (2022) meta-analysis also demonstrated that MBIs and ACTs were effective in reducing parental stress, anxiety, and depression, which supported the findings of our study discussed above; however, our pooled results showed that no significant reductions were observed for parental stress or depressive symptoms when using ACT for parents of children with DD. This may be due to the paucity of research investigating the effectiveness of ACT for parents of children with DD, which allowed for only 4 eligible studies to be included in our meta-analysis. All included studies concluded that ACT could be applied to lessen depressive symptoms, anxiety, and parental distress and to promote parental well-being, parent‒child relationships, mindful parenting, and mindful awareness, even though the conclusions for parental stress and psychological flexibility were inconsistent. The reason that these two outcomes did not change in the RCT conducted by Whittingham et al. (2022) may be because some of the parents of children with CP had to cope with not only their children’s motor problems but also other problems caused by comorbidities, such as epilepsy (14.93%), ASD (8.96%), and ADHD (4.48%), resulting in a higher level of stress and making it more difficult to actively develop personal growth (e.g., psychological flexibility) (Li et al., 2016). Furthermore, as Chua and Shorey (2022) did not further conduct a subgroup analysis to explore the effectiveness of MBIs and ACT separately, a direct comparison could not be drawn. In addition, we did not perform a subgroup analysis for DBT and CFT because only one relevant study was included in our review, respectively. However, Shareh et al. (2023) revealed that DBT significantly reduced distress and depression in mothers of children with ID postintervention compared with the wait-list control. And Khoshvaght et al. (2021) demonstrated that CFT effectively alleviated anxiety and depression in mothers of children with CP postintervention compared with no treatment. As a result, future well-designed RCTs are recommended to examine and confirm the effectiveness of ACT, DBT, and CFT on the outcomes mentioned above for parents of children with DD as well as to investigate the broad potential to confer meaningful benefits to these parents beyond those outcomes.

Apart from the impact of the intervention approach mentioned above, other moderators, including intervention duration, targeted participants, and the types of DD among the children, also revealed a significant impact on the outcomes. Consistent with previous studies showing that eight weeks of MBIs (Gotink et al., 2016) and no more than five ACT sessions (Li et al., 2021) can significantly change various health indicators postintervention, our review found that 1 to 8 weeks was regarded as the optimal duration of CBIs for parents of children with DD to reduce parental anxiety and improve parental well-being, parent‒child relationships, mindful awareness, psychology flexibility. Furthermore, for participant targets, including parent‒child dyads and parent-only participants, parent-only participants showed more positive effects for the CBI outcomes (e.g., parental stress, distress, and well-being) than parent‒child dyads. However, parent–child dyads showed greater ES than parent-only participants in parent–child relationships. This may be because parent‒child dyads could improve parent‒child interactions, thus further alleviating relational frustration, parental stress, and other mental health problems (Dennis et al., 2018). Moreover, subgroup analyses also demonstrated that CBIs were associated with significant improvements for parents of children with two different types of DD (ADHD and/or ASD) in parental stress and other outcomes, which is in line with previous studies (Hartley et al., 2019; Lee et al., 2022; Yu et al., 2019). However, considering the unique needs and experiences among parents of children with different disease types, future studies and research should be syndrome- and disease-specific. Furthermore, these subgroup analyses were complicated by the fact that format was confounded with different intervention approaches. Therefore, explore other variable that potentially affect the outcomes is another recommendation for future research to consider.

There are several limitations to consider. First, the gender imbalance among the participants, which may limit findings to mother-specific experiences, should be taken into account. The proportion of women in the sample largely reflects that mothers have historically been the primary caregivers of children with DD. However, given that there is evidence to suggest that parenting experiences, mental health, and stress outcomes may differ for fathers of children with DD (Seymour et al., 2018), future studies should consider these gender differences.

Second, because many potential moderators were not disclosed and/or reported inconsistently across studies, such as sociodemographic factors (e.g., child and parent ages, gender, race/ethnicity, family income, child symptom severity and functioning), intervention characteristics (e.g., intervention context, dosage, number of sessions, and delivery location), and measurement tools, this review was unable to evaluate them all. These moderators/variations may bring further significant heterogeneity into the meta-analyses. Therefore, more consistent reporting of these variables is required for future studies to meaningfully examine systematic moderators.

Third, a total of eight studies (38.10%) were pilot studies with a small sample size (of which the number of participants in six studies was ≤ 20 per group), which may not provide enough statistical power to identify intervention effects and thus may hinder the internal and external validity of study outcomes. Moreover, no study was assessed as having a low risk of bias, and the majority of included studies had some methodological limitations, such as a lack of information about allocation concealment, intention-to-treat analyses, and prespecified protocols, as well as unblinding the intervention to participants and intervenors and the use of self-report questionnaires. In addition, the effects of CBIs on some of the apparently unaffected outcomes, such as mindful parenting, mindful awareness, and psychological flexibility, still need to be determined due to the paucity of relevant studies. Therefore, future research with rigorously designed RCTs is recommended with different intervention approaches and with the objective of extending the list of outcomes to include currently unclear outcomes to support parents of children with DD.

Finally, this study did not conduct subgroup analysis for comparison between parents of children with medical comorbidity (such as ASD-ID, ADHD-ID, ASD-ADHD, ASD-epilepsy) versus ASD as a single medical condition, or between parents of children with or without ID. This restriction was due to lack of such information reported in the included studies. This restriction is likely to limit the generalizability of the findings reported here across all those diagnosed with ASD. One factor relevant to this has been the changes in this population resulting from changes to diagnostic criteria. More specifically, researchers using data from parents whose children were diagnosed prior to 2017 and who were funded by the National Institute of Mental Health (NIMH) in the United States were required to ensure that their sample met DSM-IV or IV-Tr criteria. These criteria explicitly excluded children from diagnosis with ASD-ADHD comorbidity; this restriction was removed in 2017 with the release of DSM-5, which permitted comorbidity and this subgroup now constitutes a significant part of the ASD population. In addition, Asperger syndrome which was previously coded separately, became part of the general ASD category. Asperger syndrome constituted a significant group of children without significant developmental delay or intellectual difficulties, and a reduced level of medical comorbidity. These broadening of criteria mean that post- 2017 parents were likely to experience more diverse levels of distress and caregiver burden. For families with DD children, more healthcare, education, and social support services, are often required and it is essential to identify them, to allocate additional resource. We also need to design and evaluate syndrome- and disease-specific interventions. It is recommended, therefore that a proportion of future studies are focussed on these specific populations, clearly describe the eligibility criteria of their participants, and use this framework to develop interventions that best meet the specific needs of their parents’ in caring for themselves and their children.

## Conclusion

The positive effects of CBIs on parental stress levels, depressive symptoms, anxiety levels, parental distress, parental well-being, and parent–child relationships were highlighted in this review. Factors that influenced intervention effects included intervention approaches and durations, participant targets, and the types of DD among the children. The current evidence should be strengthened by additional well-designed RCTs that explore and examine the process, predictive factors, and mechanism of action of the best interventions for parents of children with DD.

### Supplementary Information

Below is the link to the electronic supplementary material.

Supplementary file1 (DOCX 6490 KB)

## Data Availability

All data generated or analysed during this study are included in this published article as Supplementary information files.
