# Supplementary material for: Cognitive-Based Interventions for Improving Psychological Health and Well-Being for Parents of Children with Developmental Disabilities: A Systematic Review and Meta-analysis
Source: J Autism Dev Disord. 2023 Sep 5;54(9):3316–35. doi: 10.1007/s10803-023-06063-x (PMC11362495; doi:10.1007/s10803-023-06063-x)
Supplement: Supplementary file 1 — Supplementary file1 (DOCX 6490 KB) [file 10803_2023_6063_MOESM1_ESM.docx]

**Supplementary Content**

**Supplementary Table 1.** PICO Framework

**Supplementary Table 2.** Research strategy – PubMed

**Supplementary Table 3.** Research strategy – EmBase (Ovid)

**Supplementary Table 4.** Research strategy – PsycINFO

**Supplementary Table 5.** Research strategy – CINAHL

**Supplementary Table 6.** Research strategy – ProQuest

**Supplementary Table 7.** Research strategy – Cochrane Central Register

**Supplementary Table 8.** Excluded references after full-text screening

**Supplementary Table 9.** Characteristics of the included studies

**Supplementary Figure 1.** Sensitivity analysis for the effects of cognitive-based interventions on parental stress

**Supplementary Figure 2.** Funnel plots for the effects of cognitive-based interventions on parental stress.

**Supplementary Figure 3.** Sensitivity analysis for the effects of cognitive-based interventions on depressive symptoms.

**Supplementary Figure 4.** Funnel plots for the effects of cognitive-based interventions on depressive symptoms.

**Supplementary Figure 5.** Sensitivity analysis for the effects of cognitive-based interventions on anxiety.

**Supplementary Figure 6.** Sensitivity analysis for the effects of cognitive-based interventions on parental distress.

**Supplementary Figure 7.** Funnel plots for the effects of cognitive-based interventions on parental distress.

**Supplementary Figure 8.** Sensitivity analysis for the effects of cognitive-based interventions on parental well-being of parents.

**Supplementary Figure 9.** Sensitivity analysis for the effects of cognitive-based interventions on parent-child relationship of children with ASD.

**Supplementary Figure 10.** Sensitivity analysis for the effects of cognitive-based interventions on mindful parenting of children with ASD.

**Supplementary Figure 11.** Sensitivity analysis for the effects of cognitive-based interventions on mindful awareness of children with ASD.

**Supplementary Figure 12.** Sensitivity analysis for the effects of cognitive-based interventions on psychological flexibility of children with ASD.

**Supplementary Figure 13.** Subgroup analysis for the effects of cognitive-based interventions on parental stress based on different targeted participants.

**Supplementary Figure 14.** Subgroup analysis for the effects of cognitive-based interventions on parental distress based on different targeted participants.

**Supplementary Figure 15.** Subgroup analysis for the effects of cognitive-based interventions on parent-child relationship based on different targeted participants.

**Supplementary Figure 16.** Subgroup analysis for the effects of cognitive-based interventions on parental well-being based on different targeted participants.

**Supplementary Figure 17.** Subgroup analysis for the effects of cognitive-based interventions on parental stress based on treatment duration.

**Supplementary Figure 18.** Subgroup analysis for the effects of cognitive-based interventions on depressive symptoms based on treatment duration.

**Supplementary Figure 19.** Subgroup analysis for the effects of cognitive-based interventions on anxiety based on treatment duration.

**Supplementary Figure 20.** Subgroup analysis for the effects of cognitive-based interventions on parental distress based on treatment duration.

**Supplementary Figure 21.** Subgroup analysis for the effects of cognitive-based interventions on parental well-being based on treatment duration.

**Supplementary Figure 22.** Subgroup analysis for the effects of cognitive-based interventions on parent-child relationship based on treatment duration.

**Supplementary Figure 23.** Subgroup analysis for the effects of cognitive-based interventions on mindfulness awareness based on treatment duration.

**Supplementary Figure 24.** Subgroup analysis for the effects of cognitive-based interventions on parental psychological flexibility based on treatment duration.

**Supplementary Figure 25.** Subgroup analysis for the effects of cognitive-based interventions on parental stress based on disease types of children.

**Supplementary Figure 26.** Subgroup analysis for the effects of cognitive-based interventions on depressive symptoms based on disease types of children.

**Supplementary Figure 27.** Subgroup analysis for the effects of cognitive-based interventions on anxiety based on disease types of children.

**Supplementary Figure 28.** Subgroup analysis for the effects of cognitive-based interventions on distress based on disease types of children.

**Supplementary Figure 29.** Subgroup analysis for the effects of cognitive-based interventions on parental well-being based on disease types of children.

**Supplementary Figure 30.** Subgroup analysis for the effects of cognitive-based interventions on parent-child relationship based on disease types of children.

**Supplementary Table 1. PICO Framework**

| Population | Caregivers of children aged below 18 with diagnosed developmental disabilities (e.g., autism spectrum disorder, attention‐deficit/hyperactivity disorder, fragile X syndrome, cerebral palsy, Down syndrome, intellectual disability, intellectual disability, visual disorder, and learning disorder) |
| --- | --- |
| Interventions | **Cognitive-based interventions**   - Cognitive behavioral therapy (CBT) - Dialectical behavioral therapy (DBT) - Acceptance and Commitment therapy (ACT) - Mindfulness-based interventions   - Mindfulness-based Stress Reduction [MBSR]   - Mindfulness-based cognitive Therapy [MBCT]   - Mindfulness-based psychoeducation   - Self-compassion therapy   - Spirituality therapy - Mindfulness (other than mindfulness-based interventions) - Counselling |
| Comparator | **Active comparator**:   - Any of the interventions listed above - Attention care   **Inactive comparator**:   - Treat as usual/standard care - Waiting list - No intervention |
| Outcomes | Parental psychological outcomes (including emotional distress, stress, depression, and anxiety) |
| Studies | Randomized controlled trials (RCTs) |

**Supplementary Table 2. Research strategy – PubMed (24 April, 2023)**

| #ID | Topic or intervention | Query | Records |
| --- | --- | --- | --- |
| #1 | Disease-Developmental disabilities | (Developmental Disabilities[Mesh]) OR (Autistic Disorder[Mesh]) OR (Autism Spectrum Disorder[Mesh]) OR (Attention Deficit Disorder with Hyperactivity[Mesh]) OR (Fragile X Syndrome[Mesh]) OR (Down Syndrome[Mesh]) OR (Intellectual Disability [Mesh]) OR (Developmental Disabilities) OR Autis* OR (Autism Spectrum Disorder) OR Asperger* OR (pervasive developmental disorder*) OR (pervasive child development disorder*) OR (pervasive childhood developmental disorder*) OR ADHD OR (attention‐deficit) OR (hyperactivity disorder) OR (Fragil?x) OR (cerebral palsy) OR (intellectual disability) OR (visual disorder) OR (learning disorder) | 421,116 |
| #2 | Population-children | ((Adolescent[Mesh]) OR (Child[Mesh]) OR (Pediatrics[Mesh])) OR (adolesc*[Title/Abstract] OR child*[Title/Abstract] OR kid[Title/Abstract] OR kids[Title/Abstract] OR boy[Title/Abstract] OR boys[Title/Abstract] OR boyhood[Title/Abstract] OR girl*[Title/Abstract] OR juvenil*[Title/Abstract] OR minors[Title/Abstract] OR paediatri*[Title/Abstract] OR pediatri*[Title/Abstract] OR peadiatric*[Title/Abstract] OR pubescen*[Title/Abstract] OR school*[Title/Abstract] OR student*[Title/Abstract] OR teen*[Title/Abstract] OR young[Title/Abstract] OR youth*[Title/Abstract]) | 4,725,006 |
| #3 | Population-caregiver | (caregivers[Mesh]) OR (parent*[Title/Abstract] OR mother*[Title/Abstract] OR father*[Title/Abstract] OR maternal*[Title/Abstract] OR caregiver*[Title/Abstract]) OR (parental[Title/Abstract]) | 1,013,233 |
| #4 | Intervention - Cognitive-based interventions | (Cognitive Behavioral Therapy[Mesh]) OR (Behavioral Therap*, Cognitive[Title/Abstract]) OR (Cognitive Behavio?ral Therap*[Title/Abstract]) OR (Therap*, Cognitive Behavio?ral[Title/Abstract]) OR (Cognitive Psychotherap*[Title/Abstract]) OR (Cognitive Therap*[Title/Abstract]) OR (CBT[Title/Abstract]) OR (cognitive-based[Title/Abstract]) OR (cognitive training[Title/Abstract]) OR (cognitive intervention*[Title/Abstract]) OR (Acceptance and Commitment Therapy[Mesh]) OR (Acceptance[Title/Abstract]) OR (ACT[Title/Abstract]) OR (Commitment[Title/Abstract]) OR (Self-Compassion[Mesh]) OR (self Compassion[Title/Abstract]) OR (compassion[Title/Abstract]) OR (Mindfulness[Mesh]) OR (mind*[Title/Abstract]) OR (mindfulness-based) OR (Dialectical behavioral therapy[Title/Abstract]) OR (spirituality[Title/Abstract]) OR (Counseling[Title/Abstract]) | 728,975 |
| #5 | Outcomes | (mental*) OR (psycho*) OR (stress*) OR (anxiety*) OR (depress*) OR (distress) | 4,433,836 |
| #6 | Study type | (Randomized Controlled Trial [Publication Type]) OR (Controlled Clinical Trial[Publication Type]) OR (Randomized Clinical Trials as Topic[Mesh]) OR (Clinical Trials as Topic[Mesh]) OR Randomi*[Title/Abstract] OR (Random Allocation[Title/Abstract]) OR (Clinical Trial[Title/Abstract]) OR (Double-Blind Method[Title/Abstract]) OR (Single-Blind Method[Title/Abstract]) NOT (protocol*[Title/Abstract]) | 1,307,197 |
| #5 | Results-RCT | #1 AND #2 AND #3 AND #4 AND #5 AND #6 | 405 |

**Supplementary Table 3. Research strategy – Embase (Ovid) (24 April, 2023)**

| #ID | Topic or intervention | Query | Records |
| --- | --- | --- | --- |
| #1 | Disease- Developmental disabilities | exp Developmental Disabilities /or exp Autistic Disorder / or exp Autism Spectrum Disorder/ or exp Attention Deficit Disorder with Hyperactivity/ or exp Fragile X Syndrome/ or exp Down Syndrome/ or exp intellectual disability/ | 727,102 |
| #2 |  | (Autis* or ASD* or Asperger* or (pervasive developmental disorder*) or PDD* or (pervasive child development disorder*) or (pervasive childhood developmental disorder*) or ADHD or (attention deficit) or (hyperactivity disorder) or (Fragil?x) or (cerebral palsy) or (intellectual disability) or (visual disorder) or (learning disorder)).mp | 330,043 |
| #3 |  | 1 or 2 | 921,384 |
| #4 | Population-children | exp child/ or exp adolescent/ or exp pediatrics/ | 3,970,610 |
| #5 |  | (adolesc* or child* or kid* or boy* or girl* or juvenil* or minors or paediatri* or pediatri* or peadiatric* or pubescen* or school* or student* or teen* or young or youth*).tw. | 4,524,497 |
| #6 |  | 4 or 5 | 6,229,467 |
| #7 | Population - caregivers | exp caregivers/ | 111,534 |
| #8 |  | (parent* or mother* or father* or maternal* or caregiver* or parental or family).tw. | 2,294,995 |
| #9 |  | 7 or 8 | 2,316,149 |
| #10 | Intervention – Cognitive-based intervention | exp Cognitive Behavioral Therapy / or exp mindfulness/ or exp self-compassion/ | 39,119 |
| #11 |  | ((Cognitive Behavioral Therapy) or (Behavioral Therap*, Cognitive) or (Cognitive Behavio?ral Therap*) or (Therap*, Cognitive Behavio?ral) or (Cognitive Psychotherap*) or (Cognitive Therap*) or (CBT) or (cognitive-based) OR (cognitive training) or (cognitive intervention*) or (Acceptance and Commitment Therapy) or (Acceptance) or (ACT) or (Commitment) or (Self-Compassion) or (self Compassion) or (compassion) or (Mindfulness) or (mind*) or (mindfulness-based) or (Dialectical behavioral therapy) or (spirituality) or (Counseling)).tw. | 853,007 |
| #12 |  | 10 or 11 | 866,384 |
| #13 | Outcomes | exp caregiver burden/ | 10,457 |
| #14 |  | (mental* or psycho* or stress* or anxiety* or depress* or distress).mp | 4,639,033 |
| #15 |  | 13 or 14 | 4,641,692 |
| #16 | Study type – Quantitative evidence | exp Randomized Controlled Trial/ or exp Controlled Clinical Trial/ or exp Clinical Trials as Topic/ | 1,405,683 |
| #17 |  | (Randomi* or Random Allocation or Clinical Trial or Double-Blind or Single-Blind or Placebo* or Cross-Over Stud*).tw. | 1,483,458 |
| #18 |  | 16 or 17 | 2,071,810 |
| #19 | Results-RCT | 3 and 6 and 9 and 12 and 15 and 18 | 442 |

**Supplementary Table 4. Research strategy – PsycINFO (24 April, 2023)**

| #ID | Topic or intervention | Query | Records |
| --- | --- | --- | --- |
| #1 | Disease- Developmental disabilities | exp Developmental Disabilities /or exp Autism Spectrum Disorder/ or exp Attention Deficit Disorder with Hyperactivity/ or exp Fragile X Syndrome/ or exp Down Syndrome/ | 102,895 |
| #2 |  | (Autis* or ASD* or Asperger* or (pervasive developmental disorder*) or PDD* or (pervasive child development disorder*) or (pervasive childhood developmental disorder*) or ADHD or (attention deficit) or (hyperactivity disorder) or (Fragil?x) or (cerebral palsy) or (intellectual disability) or (visual disorder) or (learning disorder)).mp | 143,771 |
| #3 |  | 1 or 2 | 160,791 |
| #4 | Population-children | exp pediatrics/ | 35,908 |
| #5 |  | (adolesc* or child* or kid* or boy* or girl* or juvenil* or minors or paediatri* or pediatri* or peadiatric* or pubescen* or school* or student* or teen* or young or youth*).tw. | 1,763,413 |
| #6 |  | 4 or 5 | 1,767,809 |
| #7 | Population - caregivers | exp caregivers/ | 35,874 |
| #8 |  | (parent* or mother* or father* or maternal* or caregiver* or parental or family).tw. | 699,202 |
| #9 |  | 7 or 8 | 701,969 |
| #10 | Intervention – Cognitive-based intervention | exp Cognitive Therapy / or exp mindfulness/ or exp self-compassion/ | 27,367 |
| #11 |  | ((Cognitive Behavioral Therapy) or (Behavioral Therap*, Cognitive) or (Cognitive Behavio?ral Therap*) or (Therap*, Cognitive Behavio?ral) or (Cognitive Psychotherap*) or (Cognitive Therap*) or (CBT) or (cognitive-based) OR (cognitive training) or (cognitive intervention*) or (Acceptance and Commitment Therapy) or (Acceptance) or (ACT) or (Commitment) or (Self-Compassion) or (self Compassion) or (compassion) or (Mindfulness) or (mind*) or (mindfulness-based) or (Dialectical behavioral therapy) or (spirituality) or (Counseling)).tw. | 450,153 |
| #12 |  | 10 or 11 | 455,284 |
| #13 | Outcomes | exp caregiver burden/ | 7,066 |
| #14 |  | (mental* or psycho* or stress* or anxiety* or depress* or distress).mp | 2,376,484 |
| #15 |  | 13 or 14 | 2,377,762 |
| #16 | Study type – Quantitative evidence | exp Randomized Controlled Trial/ | 1,426 |
| #17 |  | (Randomi* or Random Allocation or Clinical Trial or Double-Blind or Single-Blind or Placebo* or Cross-Over Stud*).tw. | 144,360 |
| #18 |  | (((random* or control* or clinical*) adj3 (trial* or stud*)) or (random* adj3 allocat*)).tw. | 198,691 |
| #19 |  | ((singl* or doubl* or trebl* or tripl*) adj (blind* or mask*)).tw. | 28,846 |
| #20 |  | (crossover* or (cross adj over*)).tw. | 11,821 |
| #21 |  | or/16-20 | 247,290 |
| #22 | Results-RCT | 3 and 6 and 9 and 12 and 15 and 18 | 227 |

**Supplementary Table 5. Research strategy – CINAHL (24 April, 2023)**

| #ID | Topic or intervention | Query | Records |
| --- | --- | --- | --- |
| #1 | Disease- Developmental disabilities | (MH “Developmental Disabilities”) or (MH “Autistic Disorder”) or (MH “Attention Deficit Disorder with Hyperactivity”) or (MH “Fragile X Syndrome”) or (MH “Down Syndrome”) or (MH “intellectual disability”) | 67,779 |
| #2 |  | TX (Autis* or ASD* or Asperger* or (pervasive developmental disorder*) or PDD* or (pervasive child development disorder*) or (pervasive childhood developmental disorder*) or ADHD or (attention deficit) or (hyperactivity disorder) or (Fragil?x) or (cerebral palsy) or (intellectual disability) or (visual disorder) or (learning disorder)) | 211,751 |
| #3 |  | S1 OR S2 | 223,426 |
| #4 | Population - children | (MH “child”) OR (MH “adolescent”) OR (MH “pediatrics”) | 534,347 |
| #5 |  | TI (adolesc* or child* or kid* or boy* or girl* or juvenil* or minors or paediatri* or pediatri* or peadiatric* or pubescen* or school* or student* or teen* or young or youth*) OR AB (adolesc* or child* or kid* or boy* or girl* or juvenil* or minors or paediatri* or pediatri* or peadiatric* or pubescen* or school* or student* or teen* or young or youth*) | 1,282,992 |
| #6 |  | S4 OR S5 | 1,419,115 |
| #7 | Population - caregiver | (MH “caregivers”) | 42,656 |
| #8 |  | TI (parent* or mother* or father* or maternal* or caregiver* or parental or family) OR AB (parent* or mother* or father* or maternal* or caregiver* or parental or family) | 588,150 |
| #9 |  | S7 OR S8 | 600,010 |
| #10 | Intervention - Cognitive-based intervention | (MH “cognitive therapy+”) or (MH “mindfulness +”) or (MH “self-compassion+”) | 28,940 |
| #11 |  | TI ((Cognitive Behavioral Therapy) or (Behavioral Therap*, Cognitive) or (Cognitive Behavio?ral Therap*) or (Therap*, Cognitive Behavio?ral) or (Cognitive Psychotherap*) or (Cognitive Therap*) or (CBT) or (cognitive-based) OR (cognitive training) or (cognitive intervention*) or (Acceptance and Commitment Therapy) or (Acceptance) or (ACT) or (Commitment) or (Self-Compassion) or (self Compassion) or (compassion) or (Mindfulness) or (mind*) or (mindfulness-based) or (Dialectical behavioral therapy) or (spirituality) or (Counseling)) OR AB ((Cognitive Behavioral Therapy) or (Behavioral Therap*, Cognitive) or (Cognitive Behavio?ral Therap*) or (Therap*, Cognitive Behavio?ral) or (Cognitive Psychotherap*) or (Cognitive Therap*) or (CBT) or (cognitive-based) OR (cognitive training) or (cognitive intervention*) or (Acceptance and Commitment Therapy) or (Acceptance) or (ACT) or (Commitment) or (Self-Compassion) or (self compassion) or (compassion) or (Mindfulness) or (mind*) or (mindfulness-based) or (Dialectical behavioral therapy) or (spirituality) or (Counseling)) | 249,328 |
| #12 |  | S10 or S11 | 260,053 |
| #13 | Outcomes | (MH “caregiver burden”) | 11,470 |
| #14 |  | TX ((mental) or (psychological) or (psychosocial) or (stress) or (anxiety) or (depressive) or (depression) or (distress)) | 2,005,549 |
| #15 |  | S13 OR S14 | 2,007,387 |
| #16 | Study type – Quantitative evidence | (MH “Clinical Trials+”) | 349,629 |
| #17 |  | TI (Randomi* or Random Allocation or Clinical Trial or Double-Blind or Single-Blind or Placebo* or Cross-Over Stud*) OR AB (Randomi* or Random Allocation or Clinical Trial or Double-Blind or Single-Blind or Placebo* or Cross-Over Stud*) | 414,923 |
| #18 |  | S16 OR S17 | 559,913 |
| #19 | Results-RCT | S3 AND S6 AND S9 AND S12 AND S15 AND S18 | 278 |

**Supplementary Table 6. Research strategy – ProQuest (24 April, 2023)**

| #ID | Topic or intervention | Query | Records |
| --- | --- | --- | --- |
| #1 | Disease - Developmental disabilities | (“Developmental Disabilities” or “Autism Spectrum Disorder” or “Autistic Disorder” or “Attention Deficit Disorder with Hyperactivity” or “Fragile X Syndrome” or “Down Syndrome” or Autis* or ASD* or Asperger* or “pervasive developmental disorder*” or PDD* or “pervasive child development disorder*” or “pervasive childhood developmental disorder*” or “ADHD” or “attention deficit” or “hyperactivity disorder” or “Fragil?x” or “cerebral palsy” or “intellectual disability” or “visual disorder” or “learning disorder”) | 2,346,629 |
| #2 | Population - children | ti(adolesc* or child* or boy* or girl* or juvenil* or minors or paediatri* or pediatri* or pubescen* or school* or student* or teen* or young or youth*) or ab(adolesc* or child* or boy* or girl* or juvenil* or minors or paediatri* or pediatri* or pubescen* or school* or student* or teen* or young or youth*) or su(Child or Adolescence) | 20,440,594 |
| #3 | Population - caregiver | ti(parent* or mother* or father* or maternal* or caregiver* or parental) | 2,118,833 |
| #4 | Intervention - Cognitive-based intervention | (MH “cognitive therapy+”) or (MH “mindfulness +”) or (MH “self-compassion+”) | 13,166 |
| #5 |  | ti (“Cognitive Behavioral Therapy” or “Behavioral Therap*, Cognitive” or “Cognitive Behavio?ral Therap*” or “Therap*, Cognitive Behavio?ral” or “Cognitive Psychotherap*” or “Cognitive Therap*” or “CBT” or “cognitive-based” OR “cognitive training” or “cognitive intervention*” or “Acceptance and Commitment Therapy” or “Acceptance” or “ACT” or “Commitment” or “Self-Compassion” or “self Compassion” or “compassion” or “Mindfulness” or “mind*” or “mindfulness-based” or “Dialectical behavioral therapy” or “spirituality” or “Counseling”) or ab (“Cognitive Behavioral Therapy” or “Behavioral Therap*, Cognitive” or “Cognitive Behavio?ral Therap*” or “Therap*, Cognitive Behavio?ral” or “Cognitive Psychotherap*” or “Cognitive Therap*” or “CBT” or “cognitive-based” OR “cognitive training” or “cognitive intervention*” or “Acceptance and Commitment Therapy” or “Acceptance” or “ACT” or “Commitment” or “Self-Compassion” or “self Compassion” or “compassion” or “Mindfulness” or “mind*” or “mindfulness-based” or “Dialectical behavioral therapy” or “spirituality” or “Counseling”) | 3,015,534 |
| #6 |  | S4 OR S5 | 2,915,984 |
| #7 | Outcomes | (mental* or psycho* or stress* or anxiety* or depress* or distress) | 33,059,773 |
| #8 | Study type – Quantitative evidence | ti(Randomized Controlled Trial* or Randomi* or Random Allocation or Clinical Trial or Double-Blind or Single-Blind or Placebo* or Cross-Over Stud*) OR ab(Randomized Controlled Trial* or Randomi* or Random Allocation or Clinical Trial or Double-Blind or Single-Blind or Placebo* or Cross-Over Stud*) OR su(Randomized Controlled Trial* or Randomi* or Random Allocation or Clinical Trial or Double-Blind or Single-Blind or Placebo* or Cross-Over Stud*) | 1,866,023 |
| #9 | Results-RCT | S1 AND S2 AND S3 AND S6 AND S7 AND S8 | 196 |

**Supplementary Table 7. Research strategy – Cochrane Central Register (24 April, 2023)**

| #ID | Topic or intervention | Query | Records |
| --- | --- | --- | --- |
| #1 | Disease-ASD | MeSH descriptor: [Developmental Disabilities] explode all trees | 759 |
| #2 |  | MeSH descriptor: [Autistic Disorder] explode all trees | 1,355 |
| #3 |  | MeSH descriptor: [Autism Spectrum Disorder] explode all trees | 2,219 |
| #4 |  | MeSH descriptor: [Attention Deficit Disorder with Hyperactivity] explode all trees | 3,437 |
| #5 |  | MeSH descriptor: [Fragile X Syndrome] explode all trees | 126 |
| #6 |  | MeSH descriptor: [intellectual disability] explode all trees | 1817 |
| #7 |  | (Autis* OR ASD* OR Asperger* OR (pervasive developmental disorder*) OR PDD* OR (pervasive child development disorder*) OR (pervasive childhood developmental disorder*) OR (ADHD) OR (attention deficit) OR (hyperactivity disorder) OR (Fragil?x) OR (cerebral palsy) OR (intellectual disability) OR (visual disorder) OR (learning disorder)) | 31,671 |
| #8 |  | #1 OR #2 OR #3 OR #4 OR #5 OR #6 OR #7 | 32,898 |
| #9 | Population - Children | MeSH descriptor: [Child] explode all trees | 77,476 |
| #10 |  | MeSH descriptor: [Adolescent] explode all trees | 125,165 |
| #11 |  | MeSH descriptor: [Pediatrics] explode all trees | 1,178 |
| #12 |  | (adolesc*):ti,ab,kw OR (child*):ti,ab,kw OR (kid*):ti,ab,kw OR (boy*):ti,ab,kw OR (girl*):ti,ab,kw OR (juvenil*):ti,ab,kw OR (minors):ti,ab,kw OR (paediatri*):ti,ab,kw OR (pediatri*):ti,ab,kw OR (peadiatric*):ti,ab,kw OR (pubescen*) OR (school*):ti,ab,kw OR (student*):ti,ab,kw OR (teen*):ti,ab,kw OR (young):ti,ab,kw OR (youth*):ti,ab,kw | 460,606 |
| #13 |  | #9 OR #10 OR #11 OR #12 | 460,662 |
| #14 | Population - Caregivers | MeSH descriptor: [caregivers] explode all trees | 3,182 |
| #15 |  | (parent*):ti,ab,kw OR (mother*):ti,ab,kw OR (father*):ti,ab,kw OR (maternal*):ti,ab,kw OR (caregiver*):ti,ab,kw OR (parental):ti,ab,kw OR (family):ti,ab,kw | 128,346 |
| #16 |  | #14 OR #15 | 128,346 |
| #17 | Intervention - Cognitive-based intervention | MeSH descriptor: [Cognitive Behavioral Therapy] explode all trees | 12,785 |
| #18 |  | MeSH descriptor: [Mindfulness] explode all trees | 2,053 |
| #19 |  | MeSH descriptor: [Acceptance and Commitment Therapy] explode all trees | 438 |
| #20 |  | MeSH descriptor: [Self-Compassion] explode all trees | 23 |
| #21 |  | (Cognitive Behavioral Therapy):ti,ab,kw or (Behavioral Therap*, Cognitive):ti,ab,kw or (Cognitive Behavio?ral Therap*):ti,ab,kw or (Therap*, Cognitive Behavio?ral):ti,ab,kw or (Cognitive Psychotherap*):ti,ab,kw or (Cognitive Therap*):ti,ab,kw or (CBT):ti,ab,kw or (cognitive-based):ti,ab,kw or (cognitive training):ti,ab,kw or (cognitive intervention*):ti,ab,kw or (Acceptance and Commitment Therapy):ti,ab,kw or (Acceptance):ti,ab,kw or (ACT):ti,ab,kw or (Commitment):ti,ab,kw or (Self-Compassion):ti,ab,kw or (self Compassion):ti,ab,kw or (compassion):ti,ab,kw or (Mindfulness):ti,ab,kw or (mind*):ti,ab,kw or (mindfulness-based):ti,ab,kw or (Dialectical behavioral therapy):ti,ab,kw or (spirituality):ti,ab,kw or (Counseling):ti,ab,kw | 132,326 |
| #22 |  | #17 OR #18 OR #19 OR #20 OR #21 | 132,326 |
| #23 | Outcomes | MeSH descriptor: [caregiver burden] explode all trees | 154 |
| #24 |  | (mental* or psycho* or stress* or anxiety* or depress* or distress) | 362,684 |
| #25 |  | #23 OR #24 | 362,719 |
| #26 | Study type – Quantitative evidence | MeSH descriptor: [Randomized Controlled Trial] explode all trees | 25,743 |
| #27 |  | MeSH descriptor: [Controlled Clinical Trial] explode all trees | 38,488 |
| #28 |  | MeSH descriptor: [Clinical Trials as Topic] explode all trees | 84,408 |
| #29 |  | (Randomi*):ti,ab,kw OR (Random Allocation):ti,ab,kw OR (Clinical Trial):ti,ab,kw OR (Double-Blind):ti,ab,kw OR (Single-Blind):ti,ab,kw OR (Placebo*):ti,ab,kw OR (Cross-Over Stud*):ti,ab,kw | 1,323,511 |
| #30 |  | #26 OR #27 OR #28 OR #29 | 1,328,127 |
| #31 | Results-RCT | #8 AND #13 AND #16 AND #22 and #25 and #30 [limited in Trials] | 963 |

**Supplementary Table 8. Excluded references after full-text screening**

| References | Reason |
| --- | --- |
| (Ahmed & Raj, 2022) | Not RCT |
| (Bazzano et al., 2015) | Not RCT |
| (Behbahani & Zargar, 2017) | Not published in English |
| (Chu et al., 2021) | Not cognitive-based intervention |
| (Corti et al., 2018) | Not RCT |
| (de Bruin et al., 2015) | Not RCT |
| (E. M. J. C. o. i. p. Dykens, 2015) | Abstract |
| (Ede et al., 2020) | Multicomponent intervention |
| (Factor et al., 2019) | Children-targeted |
| (Flynn et al., 2020) | Multicomponent intervention |
| (Fung et al., 2018) | Not RCT |
| (Geissler et al., 2020) | Multicomponent intervention |
| (Kulbaş & Özabacı, 2022) | No relevant outcomes |
| (Häge et al., 2018) | Multicomponent intervention |
| (Hahn-Markowitz et al., 2018) | Children-targeted |
| (Haydicky et al., 2015) | Not RCT |
| (Herbert et al., 2013) | Not cognitive-based intervention |
| (Heubeck et al., 2016) | Data not available |
| (Hitchcock et al., 2022) | Children-targeted |
| (Holmberg Bergman et al., 2022) | Not RCT |
| (Jans et al., 2015) | Multicomponent intervention |
| (Jiang et al., 2018) | No relevant outcomes |
| (Kulbaş & Özabacı, 2022) | Not cognitive-based intervention |
| (Makita et al., 2023) | Not cognitive-based intervention |
| (Lunsky et al., 2021) | Not RCT |
| (Mah et al., 2021) | Multicomponent intervention |
| (Mak et al., 2018) | Multicomponent intervention |
| (Maric et al., 2018) | Children-targeted |
| (Maughan et al., 2017) | Children-targeted |
| (Miller & Brooker, 2017) | Not RCT |
| (Mueller et al., 2020) | Not RCT |
| (Namasaba et al., 2022) | No relevant outcomes |
| (Nemati et al., 2022) | Not RCT |
| (Niinomi et al., 2016) | Not RCT |
| (Nixon et al., 2002) | Data not available |
| (Novick et al., 2022) | Multicomponent intervention |
| (Padgett, 2020) | Not published in peer-review journal |
| (Petcharat & Liehr, 2021) | Not RCT |
| (A. Rickards et al., 2009) | Children-targeted |
| (A. L. Rickards et al., 2007) | Children-targeted |
| (Sayal et al., 2016) | Not cognitive-based intervention |
| (N. M. Siebelink et al., 2022) | Data not available (postintervention) |
| (N. N. Singh et al., 2020) | Mixed sample of both adolescents and adults with ASD and ID |
| (Nirbhay N Singh et al., 2019) | Not RCT |
| (N. N. Singh et al., 2021) | Multicomponent intervention |
| (Tonge et al., 2006) | Multicomponent intervention |
| (Wallander et al., 2010) | Children-targeted |
| (Weitlauf et al., 2020) | Multicomponent intervention |
| (Whittingham, Sheffield, et al., 2016) | Protocol |
| (Whittingham et al., 2019) | Multicomponent intervention |
| (Whittingham, Sanders, et al., 2016) | Multicomponent intervention |

**Supplementary Table 9. Characteristics of the included studies**

| Study ID, location | Design | Disease type | Categories (Number of participants) | Mean age, yr (range or SD) | % Female | Description of intervention (recipient, target, content, format, intensity, setting, and time point of data collection) | Outcomes (instruments) |
| --- | --- | --- | --- | --- | --- | --- | --- |
| (Behbahani et al., 2018), Iran | 2-arm RCT | ADHD | **Tx:** Mindfulness-based intervention (30)  **Ct:** No treatment (30) | **Caregiver**: NA  **Child**: NA (7-12) | **Caregiver**: NA  **Child**: 33.9 | - **Recipient (target):** Caregiver-child dyad (mix-targeted) - **Content:**    - **Tx:** Mindful parenting training: (1) Automatic pilot parenting (self-introduction, mindful eating/drinking/speaking, body scan, stress meditation); (2) Beginner’s mind (Body scan, observation of one’s child); (3) Connecting with the body in parenting (meditation, mindful movement [yoga]); (4) Being in or reacting to parental stress (mindfulness practice, mindful movement [dance]); (5) Patterns and schemas in parenting (mindfulness of emotion); (6) Conflict and parenting (Choiceless awareness + mindful movement [walking]); (7) Love and limits (loving kindness; self-compassion); (8) A mindful path (Body scan; loving kindness); (9) Beginning Anew (experience sharing/group discussion); (10) home practice (CD).   - **Ct:** No treatments - **Format**: Face-to-face, group-based - **Intensity**: 8-week, 8-seesion, 1.5-hour each session - **Setting:** Clinic of mental health care - **Time point of data collection**: Baseline, post-intervention, and 2-month follow-up after intervention | - Parental stress/distress/parent-child relationship (Parenting Stress Index-Short Form) |
| (Chronis et al., 2006), USA | 2-arm RCT | ADHD | **Tx:** CBT (25)  **Ct:** Waitlist (26) | **Caregiver**: 41.9 (6.40)  **Child**: 9.48 (5-13) | **Caregiver**: 100  **Child**: 94.1 | - **Recipient (target):** Caregivers (mix-targeted) - **Content:**   - **Tx:** Cognitive-behavioral depression treatment: relaxation training, increasing pleasurable activities, cognitive restructuring, and social skills/assertiveness training, and homework (textbook reading and homework exercises to facilitate skill building).   - **Ct:** treatment as usual and received intervention after the follow-up period - **Format**: Face-to-face, group-based - **Intensity**: 12-week, 12-seesion, NA - **Setting:** NA - **Time point of data collection**: Baseline, end of the intervention, and 5-month follow-up after intervention | - Depressive symptoms (Beck depression inventory) - Anxiety (Beck anxiety inventory) - Parental stress (Perceived stress scale) - Parent-child relationship (The Parent-Child Relationship Questionnaire) |
| (Çiçek Gümüş & Öncel, 2022), Turkey | 2-arm RCT; | ASD and mental disorders | **Tx:** ACT- based intervention (30)  **Ct:** TAU (30) | **Caregiver**: NA  **Child**: 9.48 NA | **Caregiver**: 100  **Child**: NA | - **Recipient (target):** parent only (parent-targeted) - **Content:**   - **Tx:** contacting the present moment, defusion, acceptance, self-as-context, values, and committed action.   - **Ct:** Routine training at school, including the fight against addiction, protection from abuse, communication skills, peer bullying, developmental characteristics and prevention of absenteeism. - **Format**: face-to-face, group-based - **Intensity**: 6-week, 6-session, 1-hour of each session - **Setting:** Special Education Practice Schools - **Time point of data collection**: baseline, end of the intervention, and 3-month follow-up after intervention | - Psychological symptoms (Depression Anxiety Stress Scale-21) - Parental psychological flexibility (Acceptance and Action Questionnaire-II) |
| (E. M. Dykens et al., 2014), USA | 2-arm RCT | ASD/DD | **Tx:** Mindfulness-based intervention (116)  **Ct:** Positive psychology (127) | **Caregiver**: 40.87 (23-76)  **Child**: 10.85 (2-54) | **Caregiver**: 100  **Child**: 27.7 | - **Recipient (target):** Caregivers (parent-targeted) - **Content:**   - **Tx:** Mindfulness-based stress reduction: mindfulness exercise (breathing, [setting/loving-kindness] meditation with awareness of breath, deep belly breathing, body scan), relaxation response, self-observation without self-evaluation, ang Qigong (gentle movements).   - **Ct:** Positive psychology: emphasized ways to temper such emotions as guilt, conflict, worry, and pessimism by identifying and recruiting character strengths and virtues, by using strengths in new ways, and by exercises involving gratitude, forgiveness, grace, and optimism. - **Format**: Face-to-face, group-based - **Intensity**: 6-week, 6-session, 1.5-hour each session - **Setting:** Community with optional, on-site child care for offspring with disabilities or siblings - **Time point of data collection**: Baseline, mid-intervention, end of intervention, and 4-, 12-, and 24-week follow-up after intervention | - Parenting distress (PSI) - Depression symptoms (Beck Depression Inventory) - Anxiety (Beck Anxiety Inventory) - Parental well-being (Ryff Scales of Psychological Well-Being - Short form) |
| (Feinberg et al., 2014), USA | 2-arm RCT | ASD | **Tx:** Cognitive-based intervention (53)  **Ct:** TAU (58) | **Caregiver**: 33.5 (7.0)  **Child**: 2.83 (11.0) | **Caregiver**: 100  **Child**: 16 | - **Recipient (target):** Caregivers (mix-targeted) - **Content:**   - **Tx:** Brief **c**ognitive behavioral intervention: received a manualized cognitive behavioral intervention adapted from problem-solving treatment (assessment, problem-solving, goal-setting, action-planning).   - **Ct:** Usual care group mothers received the services specified in the child’s Individualized Family Service Plan or Individualized Educational Plan. Typically, such plans include a package of services, including speech and language therapy, occupational therapy, and social skills training. - **Format**: Face-to-face, individual-based - **Intensity**: 6-session, 0.5 to 0.75-hour each session - **Setting:** Autism clinic - **Time point of data collection**: Baseline, and post-intervention (treatment group: 12-week after the first session; control group: 12-week after the baseline assessment) | - Parental Stress (Parenting Stress Index-Short Form) - Depressive Symptoms (Quick Inventory of Depressive Symptomatology) |
| (Ferraioli & Harris, 2013), USA | 2-arm RCT | ASD | **Tx:** Mindfulness-based intervention (6)  **Ct:** Skills-based intervention (9) | **Caregiver**: NA  **Child**: NA | **Caregiver**: 66.6  **Child**: NA | - **Recipient (target):** Caregivers (parent-targeted) - **Content:**   - **Tx:** Mindfulness-based cognitive therapy: observing, describing events and personal responses; nonjudgmental acceptance; distancing from thoughts; staying present; and being effective.   - **Ct:** implemented behavioral strategies at home to help effect behavior change in their child with ASD. Content included psychoeducation, behavioral strategies for increasing functional behavior (e.g., reinforcement, direct instruction, naturalistic teaching) and for decreasing challenging behavior - **Format**: Face-to-face, group-based - **Intensity**: 8-week, 8-session, 2-hour each session - **Setting:** NA - **Time point of data collection**: Baseline, end of the intervention, and 24-week follow-up after intervention | - Parental stress (Parenting Stress Index) - Parental wellbeing (General Health Questionnaire) |
| (Hahs et al., 2019), USA | 2-arm RCT | ASD | **Tx:** ACT (9)  **Ct:** No treatment (9) | **Caregiver**: 45.5 (34-57)  **Child**: 8.44 (5-13) | **Caregiver:** 72.2  **Child**: NA | - **Recipient (target):** Caregivers (parent-targeted) - **Content:**    - **Tx:** Theoretical knowledge (values ad self-as-context), creative hopelessness, cognitive defusion, acceptance, self-as-context, values and committed action.   - **Ct:** No treatments - **Format**: Face-to-face, group-based - **Intensity**: 2-day, 2-session, 2-hour each session - **Setting:** School district central office - **Time point of data collection**: Baseline, and post-intervention (1-week after the intervention) | - Depression (Beck Depression Inventory-II) - Mindful awareness (The Mindful Attention Awareness Scale) - Psychological flexibility (The Acceptance and Action Questionnaire -II) |
| (Ho et al., 2021), China (Hong Kong) | 2-arm pilot RCT | ASD | **Tx:** Mindfulness-based intervention (19)  **Ct:** Waitlist (18) | **Caregiver**: 46.5 (6.0)  **Child**: 13 (2.3) | **Caregiver**: 76  **Child**: 24 | - **Recipient (target):** Caregiver-child dyad (mix-targeted) - **Content:**   - **Tx:** Theoretical knowledge, mindfulness exercises (breathing/body/sound/thought/walking meditation and yoga practice; based on the mindfulness-based cognitive therapy and the mindfulness-based stress reduction program), experience sharing, and homework (handouts reading, instruction audio listening, and daily registration to practice mindfulness).   - **Ct:** treatment as usual and received intervention after the follow-up period - **Format**: Face-to-face, group-based - **Intensity**: 9-week, 9-session, 1.5-hour each session - **Setting:** NA - **Time point of data collection**: Baseline, and end of the intervention | - Parental stress/distress/parent-child relationship (Parenting Stress Index) - Parental well-being (WHO‑5 Well‑being Index) |
| (Khoshvaght et al., 2021), Iran | 2-arm RCT | Cerebral Palsy | **Tx:** Compassion-Focused Therapy (20)  **Ct:** No treatment (20) | **Caregiver**: 34.41 (NA)  **Child**: NA | **Caregiver**: 100  **Child**: NA | - **Recipient (target):** Caregivers (caregivers-targeted) - **Content:**   - **Tx:** Theoretical knowledge; teaching compassion and empathy; forgiveness training; valuable and sublime emotions developing; training in responsibility as the main component of self-compassion; anger and compassion practicing; summarization and reviewing.   - **Ct:** No treatment - **Format**: Face-to-face, group-based - **Intensity**: 8-week, 8-session, 1-hour each session - **Setting:** Rehabilitation Center - **Time point of data collection**: Baseline, post-intervention, and 45 days follow-up after intervention | - Anxiety (Beck Anxiety Inventory) - Depressive symptoms (Beck depression Inventory) |
| (Kuhlthau et al., 2020), USA | 2-arm Pilot RCT | ASD | **Tx:** Cognitive-based intervention (25)  **Ct:** Waitlist (26) | **Parent**: 45 (7.6)  **Child**: NA | **Parent**: 96.1  **Child**: NA | - **Recipient (target):** parents (parents-targeted) - **Content:**    - **Tx:** cognitive-behavioral therapy, which incorporated three core skills-training components (mind–body practices, cognitive-behavioral therapy skills, and positive psychology approaches).   - **Ct:** treatment as usual and received intervention after the follow-up period - **Format**: online, group-based - **Intensity**: 8-week, 8-session, 1.5-hour each session - **Setting:** no limitation - **Time point of data collection**: baseline, and post-intervention (12-week after the baseline assessment) | - Parental distress (VAS–Distress) - Depression/Anxiety (PHQ‑4) |
| (Liu et al., 2021), China (Hong Kong) | 2-arm RCT | ADHD | **Tx:** Mindfulness-based intervention (58)  **Ct:** TAU (55) | **Caregiver**: 39.76 (4.58)  **Child**: 10.03 (2.36) | **Caregiver**: 90.27  **Child**: 23.01 | - **Recipient (target):** Caregivers (mix-targeted) - **Content:**   - **Tx:** (1) Automatic pilot parenting (self-introduction, mindful eating/drinking/speaking, body scan, stress meditation); (2) Beginner’s mind (Body scan, observation of one’s child); (3) Connecting with the body in parenting (meditation, mindful movement [yoga]); (4) Being in or reacting to parental stress (mindfulness practice, mindful movement [dance]); (5) Patterns and schemas in parenting (mindfulness of emotion); (6) Conflict and parenting (Choiceless awareness + mindful movement [walking]); (7) Love and limits (loving kindness; self-compassion); (8) A mindful path (Body scan; loving kindness); (9) homework to practice mindfulness.   - **Ct:** Usual mental health care services as before. - **Format**: Face-to-face, group-based - **Intensity**: 8-week, 8-session, 3-hour each session - **Setting:** Hospital - **Time point of data collection**: Baseline, and post-intervention | - Parenting stress/distress/parent-child relationship (parenting stress index) - Depression (The Hamilton Depression Scale) - Anxiety (Hamilton Anxiety Scale) - Mindful awareness (The Five-Facet Mindfulness Questionnaire) |
| (Lobato et al., 2023), Spain | 2-arm pilot RCT | ASD and ID | **Tx:** ACT-based intervention (8)  **Ct:** Waitlist (6) | **Caregiver**: 41.2 (7.5)  **Child**: 11.9 (2.1) | **Caregiver**: 80.0  **Child**: NA | - **Recipient (target):** Caregivers (parents-targeted) - **Content:**   - **Tx:** contacting the present moment, defusion, acceptance, self-as-context, values, and committed action, and homework assignment after session 1 and 2.   - **Ct:** treatment as usual and received intervention after the follow-up period - **Format**: Face-to-face, group-based - **Intensity**: 3-week, 3-session, 3-hour each session - **Setting:** NA - **Time point of data collection**: baseline, post-intervention (one-week after the intervention), and 3-month follow-up after intervention | - Parental stress (Perceived stress scale) - Psychological wellbeing (General Health Questionnaire-12) - Psychological flexibility (Parental Acceptance Questionnaire) |
| (H. H. Lo et al., 2017), China (Hong Kong) | 2-arm pilot RCT | ASD/ADHD/DD | **Tx:** Mindfulness-based intervention (91)  **Ct:** Mindfulness workshop (89) | **Caregiver**: 38.87 (5.92)  **Child**: 5.18 (NA) | **Caregiver**: 93.9  **Child**: 22.78 | - **Recipient (target):** Caregivers (mix-targeted) - **Content:**   - **Tx:** (1) Automatic pilot parenting (self-introduction, mindful eating/drinking/speaking, body scan, stress meditation); (2) Connecting with the body in parenting (body scan); (3) Mindful perception; (4) Being in or reacting to parental stress (mindfulness practice); (5) Love and limits (loving kindness; self-compassion); (6) Review and consolidation; (7) home practice (audio tracks).   - **Ct:** 1-day mindfulness workshop. - **Format**: Face-to-face, group-based - **Intensity**: 6-week, 6-session, 1.5-hour each session - **Setting:** Family service centers - **Time point of data collection**: Baseline, and end of the intervention | - Parental stress/distress/parent-child relationship (Parenting Stress Index-SF) - Depression (The Center for Epidemiologic Studies Depression Scale) - Mindful parenting (The Interpersonal Mindfulness in Parenting) |
| (H. H. M. Lo et al., 2017), China (Hong Kong) | 2-arm RCT | ADHD | **Tx:** Mindfulness-based intervention (50)  **Ct:** Waitlist (50) | **Caregiver**: 6.25 (5-7)  **Child**: 39.21 (4.66) | **Caregiver**: 88  **Child**: 17 | - **Recipient (target):** Caregiver-child dyad (mix-targeted) - **Content:**   - **Tx:** (1) Introduction (body scan); (2) Automatic reactions (Choiceless/nonjudgmental awareness); (3) respond to children mindfully (mindfulness breath practice); (4) joint session (practice with children, progress review); (5) Love and limits (loving kindness; self-compassion); (6) joint session (practice with children, self-care of parents, review and consolidation, make care plan).   - **Ct:** treatment as usual and received intervention after the follow-up period - **Format**: Face-to-face, group-based - **Intensity**: 6-week, 6-session, 1.5-hour each session - **Setting:** Family service centers - **Time point of data collection**: Baseline, and end of the intervention | - Parental stress/distress/parent-child relationship (Parenting Stress Index-SF) - Mindful parenting (The Interpersonal Mindfulness in Parenting) - Parental well-being (World Health Organization Well-Being Index) |
| (Marino et al., 2021), Italy | 2-arm RCT | ASD | **Tx:** ACT (10)  **Ct:** Parent training (10) | **Caregiver**: 40.6 (5.34)  **Child**: 6.9 (1.66) | **Caregiver**: NA  **Child**: NA | - **Recipient (target):** Caregivers (mix-targeted) - **Content:**   - **Tx:** introduction, values identification, defusion, mindfulness, acceptance, experiential avoidance, committed action, recognition of barriers, definition of goals, verbal Aikido, self-compassion, assessment, homework to practice ACT.   - **Ct:** Training parents to teach behavioral management skills for children, plus homework. - **Format**: face-to-face, group-based - **Intensity**: 24-week, 24-meeting, 1.5-hour of each meeting - **Setting:** the Messina unit of CNR-IRIB in Messina - **Time point of data collection**: Baseline, and end of the intervention | - Parental stress/distress/parent-child relationship (Parenting Stress Index: Short Form) - Psychological flexibility (AAQ-II) - Mindful awareness (MAAS) |
| (Neece, 2014), USA | 2-arm pilot RCT | ASD/DD | **Tx:** Mindfulness-based intervention (39)  **Ct:** Waitlist (41) | **Caregiver**: 37.21 (7.22)  **Child**: 4.18 (1.01) | **Caregiver**: 96.3  **Child**: 28.75 | - **Recipient (target):** Caregiver-child dyad (mix-targeted) - **Content:**   - **Tx:** Mindfulness-Based Stress Reduction: theoretical knowledge, mindfulness exercise (body scan, sitting meditation with awareness of breath and mindful movement), experience sharing/group discussion, and home practice (CD).   - **Ct:** treatment as usual and received intervention after the follow-up period - **Format**: Face-to-face, group-based - **Intensity**: 8-week, 8-session, 2-hour each session, a daylong 6-hour meditation retreat after session 6 - **Setting:** Medical Center - **Time point of data collection**: Baseline, end of the intervention, and 6-month follow-up after intervention | - Parental stress (Parenting Stress Index-Short Form) - Depressive symptoms (Center for Epidemiological Studies Depression Scale) |
| (Onyishi et al., 2023), Nigeria | 2-arm RCT | ASD | **Tx:** Cognitive behavioral therapy (48)  **Ct:** Waitlist (47) | **Caregiver**: 40.95 (NA)  **Child**: NA | **Caregiver**: 68.04  **Child**: NA | - **Recipient (target):** Caregivers (parents-targeted) - **Content:**   - **Tx:** Specific strategies used were assessments, goal-setting, progressive relaxation techniques, discussion, disputation, cognitive restructuring, problem-solving skills, desensitization, unconditional self-acceptance, Guided imagery, rationalizing, reframing, homework assignment.   - **Ct:** treatment as usual and received intervention after the follow-up period - **Format**: Face-to-face, group-based - **Intensity**: 12-week, 12-seesion, 2-hour each session - **Setting:** town hall - **Time point of data collection**: baseline, post-intervention (two-week after the intervention), and 3-month follow-up after intervention | - Parental stress/depressive symptoms/anxiety (Depression Anxiety Stress Scale-21) |
| (Pandya, 2021), India | 2-arm RCT | ASD | **Tx:** Mindfulness-based intervention (79)  **Ct:** Waitlist (58) | **Caregiver**: 34.82 (NA)  **Child**: 5.73 (NA) | **Caregiver**: 100  **Child**: NA | - **Recipient (target):** Caregivers (mix-targeted) - **Content:**    - **Tx:** spiritual posts intervention (values identification, acceptance, meditation, relational consciousness, mindful dealing with difficult feelings), plus homework (jotting down one’s thoughts and feelings).   - **Ct:** treatment as usual and received intervention after the follow-up period - **Format**: Online, individual-based - **Intensity**: Once-a-week post for 50-weeks - **Setting:** No limitation - **Time point of data collection**: Baseline, and post-intervention (within 2-week after intervention) | - Parenting stress/distress/ parent-child relationship (Parenting Stress Index: Short Form) |
| (Schwartzman et al., 2022), USA | 2-arm pilot RCT | ASD | **Tx:** Mindfulness-base intervention: (17)  **Ct:** Waitlist (17) | **Caregiver**: 41 (NA)  **Child**: 6.8 (4-10) | **Caregiver**: 76.5  **Child**: 17.6 | - **Recipient (target):** Caregivers (mix-targeted) - **Content:**    - **Tx:** incorporated acceptance, mindfulness, optimism, resilience method, in improving parental resilience, stress management, and other domains of well-being. the majority of sessions utilized principles of CBT, such as psychoeducation, behavioral activation, identifying cognitive distortions, cognitive restructuring, and weekly homework assignments and review.   - **Ct:** treatment as usual and received intervention after the follow-up period - **Format**: Face-to-face, group-based - **Intensity**: 8-week, 8-session, 1.5-hour of each session - **Setting:** Outpatient clinic - **Time point of data collection**: Baseline, end of the intervention, and 8-week follow-up after intervention | - Parental stress/depressive symptoms/anxiety (Depression Anxiety Stress Scale) - Parenting stress/distress/ parent-child relationship (Parenting Stress Index: Short Form) - Mindful awareness (MAAS) - Psychological flexibility (AAQ-II) |
| (Shareh & Yazdanian, 2023), Iran | 2-arm RCT | ID | **Tx:** Dialectical behavior therapy (67)  **Ct:** Waitlist (66) | **Caregiver**: 36.55 (6.56)  **Child**: NA | **Caregiver**: 100  **Child**: NA | - **Recipient (target):** Caregivers (parents-targeted) - **Content:**   - **Tx:** Specific strategies used were assessments, mindfulness, relationship care and maintaining, self-respect, emotion identify and regulation, acceptance, self-awareness, and homework.   - **Ct:** treatment as usual and received intervention after the follow-up period - **Format**: Face-to-face, group-based - **Intensity**: 10-week, 10-session, 1.5-hour each session - **Setting:** NA - **Time point of data collection**: baseline, and end of the intervention | - Parental depression (Beck Depression Inventory) - Parental distress (Depression Anxiety Stress Scale) |
| (Sharif et al., 2015), Iran | 3-arm RCT | ADHD | **Tx:** Cognitive-behavioral stress management (28)  **Ct_1_:** No treatment (28)  **Ct_2_:** Placebo (30) | **Caregiver**: 34.07 (6.04)  **Child**: 8.92 (6-12) | **Caregiver**: 100  **Child**: 23.5 | - **Recipient (target):** Caregivers (caregiver-targeted) - **Content:**   - **Tx:** Assessment, training the mothers about ADHD, understanding stress and its effects, training Benson's relaxation technique, negative thoughts and cognitive distortions, replacing rational thoughts, efficient coping and implementing effective coping responses, anger management, and summary of content.   - **Ct_1_:** No treatment.   - **Ct_2_:** Only participated in meetings. - **Format**: Face-to-face, group-based - **Intensity**: 8-week, 8-session, 1.5-hour each session - **Setting:** Psychiatric clinics - **Time point of data collection**: Baseline, end of intervention, and 1-month follow-up after intervention | - Parental stress/depressive symptoms/anxiety (Depression Anxiety Stress Scale) - Parental well-being (General Heath Questionnaire) |
| (Nienke M. Siebelink et al., 2018), Netherland | 2-arm RCT | ADHD | **Tx:** Mindfulness-based intervention + TAU (55)  **Ct:** TAU (48) | **Caregiver**: 43.4 (7.52)  **Child**: 11.2 (8-16) | **Caregiver**: 67.96  **Child**: 30.10 | - **Recipient (target):** Caregiver-child dyad (mix-targeted) - **Content:**   - **Tx:** Mymind program: mindfulness and yoga   - **Ct:** Care as usual - **Format**: Face-to-face, individual-based - **Intensity**: 8-week, 8-session, 1.5-hour each session, followed by a booster session - **Setting:** Psychiatric clinics - **Time point of data collection**: baseline, end of the intervention, and 2- and 6-month follow-up after intervention | - Distress (Depression Anxiety Stress Scale) - Parental well-being (WHO-5) - Mindful parenting (The Interpersonal Mindfulness in Parenting) |
| (Valero et al., 2022), Spain | 2-arm pilot RCT | ADHD | **Tx:** Mindfulness-based intervention (15)  **Ct:** Waitlist (15) | **Caregiver**: 46 (4.50)  **Child**: 10.6 (1.58) | **Caregiver**: 96.67  **Child**: 23.33 | - **Recipient (target):** Caregiver-child dyad (mix-targeted) - **Content:**   - **Tx:** Mymind program   - **Ct:** treatment as usual and received intervention after follow-up period - **Format**: Face-to-face, group-based - **Intensity**: 8-week, 8-session, 1.5-hour each session (children separately conducted 1-h each session) - **Setting:** NA - **Time point of data collection**: baseline, end of intervention, and 6-month follow-up after intervention | - Parenting distress/ parent-child relationship (Parenting Stress Index: Short Form) |
| (Whittingham et al., 2022), Australia | 2-arm RCT | CP | **Tx:** ACT (37)  **Ct:** Waitlist (30) | **Caregiver**: NA  **Child**: 5.58 (2-10) | **Caregiver**: 94.03  **Child**: 28.36 | - **Recipient (target):** Caregiver-child dyad (mix-targeted) - **Content:**   - **Tx:** Teaching the core ACT process, including values, cognitive defusion, acceptance, mindfulness, flexible perspective taking, and committed action.   - **Ct:** treatment as usual and received intervention after follow-up period - **Format**: Online, individual-based - **Intensity**: 10-week, 3-session, 2-hour each session, a break for practice and personal study of the content, and then 1 review session (1h) - **Setting:** No limitation - **Time point of data collection**: baseline, end of intervention, and 6-month follow-up after intervention | - Mindful parenting (Interpersonal Mindfulness in Parenting Scale) - Depression/anxiety/stress (Depression Anxiety Stress Scale) - Psychological flexibility (Acceptance and Action Questionnaire) - Parental well-being (personal wellbeing index) |
| (Wong et al., 2010), Australia | 2-arm RCT | ADHD | **Tx:** CBT (29)  **Ct:** Waitlist (29) | **Caregiver**: 47 (37-60)  **Child**: NA | **Caregiver**: 95  **Child**: NA | - **Recipient (target):** Caregivers (caregiver-targeted) - **Content:**   - **Tx:** Session 1: the participants were helped to understand the patterns of their physiological, cognitive, behavioral, and emotional responses to external stressful life events. Sessions 2-3: aimed to help the participants to understand their own types of negative automatic thought patterns (i.e. cognitive distortions) and dysfunctional coping behavior in relation to their depressed mood. Sessions 4-5: the participants learned various cognitive and behavioral strategies to manage their depressed mood. Session 6: focused on helping the participants to understand their dysfunctional attitudes, rules, and values. Sessions 7-8: the participants were introduced to various strategies for challenging and modifying their dysfunctional rules and values. Session 9: the participants had the opportunity to examine their priorities in life.   - **Ct:** treatment as usual and received intervention after the follow-up period - **Format**: Face-to-face, group-based - **Intensity**: 10-week, 10-session, 3-hour each session - **Setting:** Community - **Time point of data collection**: baseline, post-intervention (specific time is not avaliable), and 6-month follow-up after intervention | - Parental wellbeing (GHQ-12) - Parental stress (PSI) |

References:

Ahmed, A. N., & Raj, S. P. J. A. i. N. D. (2022). Self-Compassion Intervention for Parents of Children with Developmental Disabilities: A Feasibility Study. 1-13.

Bazzano, A., Wolfe, C., Zylowska, L., Wang, S., Schuster, E., Barrett, C., . . . Studies, F. (2015). Mindfulness based stress reduction (MBSR) for parents and caregivers of individuals with developmental disabilities: A community-based approach. *24*(2), 298-308.

Behbahani, M., Zargar, F., Assarian, F., & Akbari, H. (2018). Effects of Mindful Parenting Training on Clinical Symptoms in Children with Attention Deficit Hyperactivity Disorder and Parenting Stress: Randomized Controlled Trial. *Iranian journal of medical sciences, 43*(6), 596-604.

Behbahani, M., & Zargar, F. J. J. o. I. M. S. (2017). Effectiveness of mindful parenting training on clinical symptoms and self-efficacy in children with attention deficit hyperactivity disorder. *35*(429), 511-517.

Chronis, A. M., Gamble, S. A., Roberts, J. E., & Pelham Jr, W. E. J. B. t. (2006). Cognitive-behavioral depression treatment for mothers of children with attention-deficit/hyperactivity disorder. *37*(2), 143-158.

Chu, L., Zhu, P., Ma, C., Pan, L., Shen, L., Wu, D., . . . Yu, G. J. F. i. P. (2021). Effects of Combing Group Executive Functioning and Online Parent Training on School-Aged Children With ADHD: A Randomized Controlled Trial. 1655.

Çiçek Gümüş, E., & Öncel, S. (2022). Effects of Acceptance and Commitment Therapy-based interventions on the mental states of parents with special needs children: Randomized controlled trial. *Current Psychology*. doi:10.1007/s12144-022-03760-1

Corti, C., Pergolizzi, F., Vanzin, L., Cargasacchi, G., Villa, L., Pozzi, M., . . . Studies, F. (2018). Acceptance and commitment therapy-oriented parent-training for parents of children with autism. *27*(9), 2887-2900.

de Bruin, E. I., Blom, R., Smit, F. M., van Steensel, F. J., & Bögels, S. M. J. A. (2015). MYmind: Mindfulness training for youngsters with autism spectrum disorders and their parents. *19*(8), 906-914.

Dykens, E. M., Fisher, M. H., Taylor, J. L., Lambert, W., & Miodrag, N. (2014). Reducing distress in mothers of children with autism and other disabilities: a randomized trial. *Pediatrics, 134*(2), e454-463. doi:10.1542/peds.2013-3164

Dykens, E. M. J. C. o. i. p. (2015). Family adjustment and interventions in neurodevelopmental disorders. *28*(2), 121.

Ede, M. O., Anyanwu, J. I., Onuigbo, L. N., Ifelunni, C. O., Alabi-Oparaocha, F. C., Okenyi, E. C., . . . Therapy, C.-B. (2020). Rational emotive family health therapy for reducing parenting stress in families of children with autism spectrum disorders: a group randomized control study. *38*(2), 243-271.

Factor, R. S., Swain, D. M., Antezana, L., Muskett, A., Gatto, A. J., Radtke, S. R., & Scarpa, A. J. B. o. t. M. C. (2019). Teaching emotion regulation to children with autism spectrum disorder: Outcomes of the Stress and Anger Management Program (STAMP). *83*(3), 235-258.

Feinberg, E., Augustyn, M., Fitzgerald, E., Sandler, J., Suarez, Z. F. C., Chen, N., . . . Silverstein, M. (2014). Improving maternal mental health after a child's diagnosis of autism spectrum disorder: Results from a randomized clinical trial. *JAMA Pediatr, 168(1)*, 40-46.

Ferraioli, S. J., & Harris, S. L. (2013). Comparative effects of mindfulness and skills-based parent training programs for parents of children with autism: Feasibility and preliminary outcome data. *Special Issue: Mindfulness and acceptance in developmental disabilities, 4*(2), 89-101. doi:https://dx.doi.org/10.1007/s12671-012-0099-0

Flynn, S., Hastings, R. P., Burke, C., Howes, S., Lunsky, Y., Weiss, J. A., & Bailey, T. J. M. (2020). Online mindfulness stress intervention for family carers of children and adults with intellectual disabilities: Feasibility randomized controlled trial. *11*(9), 2161-2175.

Fung, K., Lake, J., Steel, L., Bryce, K., Lunsky, Y. J. J. o. a., & disorders, d. (2018). ACT processes in group intervention for mothers of children with autism spectrum disorder. *48*(8), 2740-2747.

Geissler, J. M., Vloet, T. D., Strom, N., Jaite, C., Graf, E., Kappel, V., . . . Psychiatry, A. (2020). Does helping mothers in multigenerational ADHD also help children in the long run? 2-year follow-up from baseline of the AIMAC randomized controlled multicentre trial. *29*(10), 1425-1439.

Häge, A., Alm, B., Banaschewski, T., Becker, K., Colla, M., Freitag, C., . . . psychiatry, a. (2018). Does the efficacy of parent–child training depend on maternal symptom improvement? Results from a randomized controlled trial on children and mothers both affected by attention-deficit/hyperactivity disorder (ADHD). *27*(8), 1011-1021.

Hahn-Markowitz, J., Berger, I., Manor, I., Maeir, A. J. P., & Pediatrics, O. T. i. (2018). Cognitive-functional (cog-fun) dyadic intervention for children with ADHD and their parents: Impact on parenting self-efficacy. *38*(4), 444-456.

Hahs, A. D., Dixon, M. R., & Paliliunas, D. (2019). Randomized controlled trial of a brief acceptance and commitment training for parents of individuals diagnosed with autism spectrum disorders. *Journal of Contextual Behavioral Science, 12*, 154-159. doi:https://dx.doi.org/10.1016/j.jcbs.2018.03.002

Haydicky, J., Shecter, C., Wiener, J., Ducharme, J. M. J. J. o. c., & studies, f. (2015). Evaluation of MBCT for adolescents with ADHD and their parents: Impact on individual and family functioning. *24*(1), 76-94.

Herbert, S. D., Harvey, E. A., Roberts, J. L., Wichowski, K., & Lugo-Candelas, C. I. J. B. T. (2013). A randomized controlled trial of a parent training and emotion socialization program for families of hyperactive preschool-aged children. *44*(2), 302-316.

Heubeck, B. G., Otte, T. A., & Lauth, G. W. J. B. J. o. C. P. (2016). Consumer evaluation and satisfaction with individual versus group parent training for children with hyperkinetic disorder (HKD). *55*(3), 305-319.

Hitchcock, C., Goodall, B., Wright, I. M., Boyle, A., Johnston, D., Dunning, D., . . . Psychiatry. (2022). The early course and treatment of posttraumatic stress disorder in very young children: diagnostic prevalence and predictors in hospital‐attending children and a randomized controlled proof‐of‐concept trial of trauma‐focused cognitive therapy, for 3‐to 8‐year‐olds. *63*(1), 58-67.

Ho, R. Y. F., Zhang, D., Chan, S. K. C., Gao, T. T., Lee, E. K. P., Lo, H. H. M., . . . et al. (2021). Brief Report: mindfulness Training for Chinese Adolescents with Autism Spectrum Disorder and Their Parents in Hong Kong. *J Autism Dev Disord, 51*(11), 4147‐4159. doi:10.1007/s10803-020-04729-4

Holmberg Bergman, T., Renhorn, E., Berg, B., Lappalainen, P., Ghaderi, A., Hirvikoski, T. J. J. o. A., & Disorders, D. (2022). Acceptance and Commitment Therapy Group Intervention for Parents of Children with Disabilities (Navigator ACT): An Open Feasibility Trial. 1-16.

Jans, T., Jacob, C., Warnke, A., Zwanzger, U., Groß‐Lesch, S., Matthies, S., . . . Psychiatry. (2015). Does intensive multimodal treatment for maternal ADHD improve the efficacy of parent training for children with ADHD? A randomized controlled multicenter trial. *56*(12), 1298-1313.

Jiang, Y., Haack, L. M., Delucchi, K., Rooney, M., Hinshaw, S. P., McBurnett, K., & Pfiffner, L. J. J. B. t. (2018). Improved parent cognitions relate to immediate and follow-up treatment outcomes for children with ADHD-Predominantly Inattentive Presentation. *49*(4), 567-579.

Khoshvaght, N., Naderi, F., Safarzadeh, S., & Alizadeh, M. J. A. o. H. S. (2021). The Effects of Compassion-focused Therapy on Anxiety and Depression in the Mothers of Children With Cerebral Palsy. *10*(3), 225-234.

Kulbaş, E., & Özabacı, N. (2022). The Effects of the Positive Psychology-Based Online Group Counselling Program on Mothers Having Children with Intellectual Disabilities. *Journal of Happiness Studies, 23*(5), 1817-1845. doi:10.1007/s10902-021-00472-4

Liu, P., Qiu, S., Lo, H. H. M., Song, X., & Qian, Q. J. M. (2021). Applying the mindful parenting program among Chinese parents of children with ADHD: A randomized control trial. *12*(6), 1473-1489.

Lo, H. H., Wong, S. W., Wong, J. Y., Yeung, J. W., Snel, E., & Wong, S. Y. J. J. o. a. d. (2017). The effects of family-based mindfulness intervention on ADHD symptomology in young children and their parents: A randomized control trial. *24*(5), 667-680.

Lo, H. H. M., Chan, S. K. C., Szeto, M. P., Chan, C. Y. H., & Choi, C. W. J. M. (2017). A feasibility study of a brief mindfulness-based program for parents of preschool children with developmental disabilities. *8*(6), 1665-1673.

Lobato, D., Montesinos, F., Polín, E., & Cáliz, S. (2023). Third-Generation Behavioural Therapies in the Context of Neurodevelopmental Problems and Intellectual Disabilities: A Randomised Clinical Trial with Parents. *International Journal of Environmental Research and Public Health, 20*(5), 4406.

Lunsky, Y., Albaum, C., Baskin, A., Hastings, R., Hutton, S., Steel, L., . . . Disorders, D. (2021). Group virtual mindfulness-based intervention for parents of autistic adolescents and adults. *51*(11), 3959-3969.

Mah, J. W., Murray, C., Locke, J., & Carbert, N. J. J. o. A. D. (2021). Mindfulness-enhanced behavioral parent training for clinic-referred families of children with ADHD: A randomized controlled trial. *25*(12), 1765-1777.

Mak, C., Whittingham, K., Cunnington, R., Boyd, R. N. J. D. M., & Neurology, C. (2018). Effect of mindfulness yoga programme MiYoga on attention, behaviour, and physical outcomes in cerebral palsy: a randomized controlled trial. *60*(9), 922-932.

Makita, K., Yao, A., Shimada, K., Kasaba, R., Fujisawa, T. X., Mizuno, Y., & Tomoda, A. (2023). Neural and behavioral effects of parent training on emotion recognition in mothers rearing children with attention-deficit/hyperactivity disorder. *Brain Imaging Behav*. doi:10.1007/s11682-023-00771-9

Maric, M., van Steensel, F. J., & Bögels, S. M. J. J. o. A. D. (2018). Parental involvement in CBT for anxiety-disordered youth revisited: family CBT outperforms child CBT in the long term for children with comorbid ADHD symptoms. *22*(5), 506-514.

Marino, F., Failla, C., Chila, P., Minutoli, R., Puglisi, A., Arnao, A. A., . . . Pioggia, G. (2021). The effect of acceptance and commitment therapy for improving psychological well-being in parents of individuals with autism spectrum disorders: A randomized controlled trial. *Brain Sci, 11(7) (no pagination)*(880).

Maughan, A. L., Weiss, J. A. J. J. o. A., & Disorders, D. (2017). Parental outcomes following participation in cognitive behavior therapy for children with autism spectrum disorder. *47*(10), 3166-3179.

Miller, C. J., & Brooker, B. J. C. t. i. c. p. (2017). Mindfulness programming for parents and teachers of children with ADHD. *28*, 108-115.

Mueller, R., Moskowitz, L. J. J. J. o. C., & Studies, F. (2020). Positive family intervention for children with ASD: impact on parents’ cognitions and stress. *29*(12), 3536-3551.

Namasaba, M., Nabunje, S., & Baguwemu, A. A. (2022). Effectiveness of multi-modal cognitive behavioural therapy in improving mental well-being among caregivers of children with disabilities in urban Uganda: A cluster-randomized controlled trial. *J Glob Health, 12*, 04102. doi:10.7189/jogh.12.04102

Neece, C. L. (2014). Mindfulness-based stress reduction for parents of young children with developmental delays: implications for parental mental health and child behavior problems. *Journal of Applied Research in Intellectual Disabilities, 27*(2), 174‐186. doi:10.1111/jar.12064

Nemati, S., Shojaeian, N., Martínez-González, A. E., Hosseinkhanzadeh, A. A., Katurani, A., & Khiabani, I. J. I. J. o. D. D. (2022). Maternal acceptance–rejection, self-compassion and empathy in mothers of children with intellectual and developmental disabilities. *68*(2), 102-106.

Niinomi, K., Asano, M., Kadoma, A., Yoshida, K., Ohashi, Y., Furuzawa, A., . . . Sciences, H. (2016). Developing the “Skippu‐Mama” program for mothers of children with autism spectrum disorder. *18*(3), 283-291.

Nixon, C. D., Singer, G. J. T. B. o. A. F., & Century, M. R. a. C. o. N. A. J. A. A. t. t. (2002). Group cognitive-behavioral treatment for excessive parental self-blame and guilt. 331.

Novick, D. R., Lorenzo, N. E., Danko, C. M., & Tuscano, A.-C. (2022). Evaluation of an Integrated Parenting Intervention Targeting Maternal Depression: Effects on Parent Attributions of Child Behaviors. *Journal of Child and Family Studies, 31*(8), 2077-2090. doi:10.1007/s10826-022-02267-4

Onyishi, C. N., Sefotho, M. M., & Victor-Aibodion, V. (2023). Psychological distress among parents of children with autism spectrum disorders: A randomized control trial of cognitive behavioural therapy. *Research in Autism Spectrum Disorders, 100*, 102070. doi:https://doi.org/10.1016/j.rasd.2022.102070

Padgett, E. E. (2020). *An Online Randomized Controlled Trial of Mindful Parenting among Parents of Children with Autism Spectrum Disorder.* Northern Illinois University,

Pandya, S. P. (2021). Examining the Effectiveness of WhatsApp-Based Spiritual Posts on Mitigating Stress and Building Resilience, Maternal Confidence and Self-efficacy Among Mothers of Children with ASD. *Journal of Autism & Developmental Disorders, 51*(5), 1479-1495. doi:10.1007/s10803-020-04633-x

Petcharat, M., & Liehr, P. J. A. o. P. N. (2021). Feasibility of a brief mindfulness intervention: Examining stress, anxiety and mindfulness for Thai parents of children with developmental disabilities. *35*(5), 418-426.

Rickards, A., Walstab, J., Wright‐Rossi, R., Simpson, J., Reddihough, D. J. C. c., health, & development. (2009). One‐year follow‐up of the outcome of a randomized controlled trial of a home‐based intervention programme for children with autism and developmental delay and their families. *35*(5), 593-602.

Rickards, A. L., Walstab, J. E., Wright-Rossi, R. A., Simpson, J., Reddihough, D. S. J. J. o. D., & Pediatrics, B. (2007). A randomized, controlled trial of a home-based intervention program for children with autism and developmental delay. *28*(4), 308-316.

Sayal, K., Taylor, J. A., Valentine, A., Guo, B., Sampson, C. J., Sellman, E., . . . development. (2016). Effectiveness and cost‐effectiveness of a brief school‐based group programme for parents of children at risk of ADHD: a cluster randomised controlled trial. *42*(4), 521-533.

Schwartzman, J. M., Millan, M. E., Uljarevic, M., & Gengoux, G. W. (2022). Resilience Intervention for Parents of Children with Autism: Findings from a Randomized Controlled Trial of the AMOR Method. *J Autism Dev Disord, 52*(2), 738-757. doi:10.1007/s10803-021-04977-y

Shareh, H., & Yazdanian, M. (2023). The effectiveness of dialectical behavior group therapy on stress, depression, and cognitive emotion regulation in mothers of intellectually disabled students: A randomized clinical trial. *Clin Child Psychol Psychiatry*, 13591045231163068. doi:10.1177/13591045231163068

Sharif, F., Zarei, S., Shooshtari, A. A., & Vossoughi, M. J. I. J. o. P. (2015). The effect of stress management program using cognitive behavior approach on mental health of the mothers of the children with attention deficit hyperactivity disorder. *25*(3).

Siebelink, N. M., Bogels, S. M., Boerboom, L. M., de Waal, N., Buitelaar, J. K., Speckens, A. E., & Greven, C. U. (2018). Mindfulness for children with ADHD and Mindful Parenting (MindChamp): Protocol of a randomised controlled trial comparing a family Mindfulness-based intervention as an add-on to care-as-usual with care-as-usual only. *BMC Psychiatry, 18*. doi:https://dx.doi.org/10.1186/s12888-018-1811-y

Siebelink, N. M., Bögels, S. M., Speckens, A. E. M., Dammers, J. T., Wolfers, T., Buitelaar, J. K., & Greven, C. U. (2022). A randomised controlled trial (MindChamp) of a mindfulness-based intervention for children with ADHD and their parents. *J Child Psychol Psychiatry, 63*(2), 165-177. doi:10.1111/jcpp.13430

Singh, N. N., Lancioni, G. E., Karazsia, B. T., Myers, R. E., Hwang, Y.-S., & Anālayo, B. J. F. i. p. (2019). Effects of mindfulness-based positive behavior support (MBPBS) training are equally beneficial for mothers and their children with autism spectrum disorder or with intellectual disabilities. *10*, 385.

Singh, N. N., Lancioni, G. E., Medvedev, O. N., Hwang, Y. S., & Myers, R. E. (2021). A Component Analysis of the Mindfulness-Based Positive Behavior Support (MBPBS) Program for Mindful Parenting by Mothers of Children with Autism Spectrum Disorder. *Mindfulness (N Y), 12*(2), 463-475. doi:10.1007/s12671-020-01376-9

Singh, N. N., Lancioni, G. E., Medvedev, O. N., Hwang, Y. S., Myers, R. E., & Townshend, K. (2020). Using mindfulness to improve quality of life in caregivers of individuals with intellectual disabilities and autism spectrum disorder. *Int J Dev Disabil, 66*(5), 370-380. doi:10.1080/20473869.2020.1827211

Tonge, B., Brereton, A., Kiomall, M., Mackinnon, A., King, N., Rinehart, N. J. J. o. t. A. A. o. C., & Psychiatry, A. (2006). Effects on parental mental health of an education and skills training program for parents of young children with autism: A randomized controlled trial. *45*(5), 561-569.

Valero, M., Cebolla, A., & Colomer, C. J. J. o. a. d. (2022). Mindfulness training for children with ADHD and their parents: a randomized control trial. *26*(5), 755-766.

Wallander, J. L., McClure, E., Biasini, F., Goudar, S. S., Pasha, O., Chomba, E., . . . Chakraborty, H. J. B. p. (2010). Brain research to ameliorate impaired neurodevelopment-home-based intervention trial (BRAIN-HIT). *10*(1), 1-9.

Weitlauf, A. S., Broderick, N., Stainbrook, J. A., Lounds Taylor, J., Herrington, C. G., Nicholson, A. G., . . . Warren, Z. E. (2020). Mindfulness-Based Stress Reduction for Parents Implementing Early Intervention for Autism: An RCT. *Pediatrics, 145*, 81-92. doi:10.1542/peds.2019-1895K

Whittingham, K., Sanders, M. R., McKinlay, L., Boyd, R. N. J. J. o. C., & Studies, F. (2019). Parenting intervention combined with acceptance and commitment therapy: Processes of change. *28*(6), 1673-1680.

Whittingham, K., Sanders, M. R., McKinlay, L., & Boyd, R. N. J. J. o. P. P. (2016). Parenting intervention combined with acceptance and commitment therapy: A trial with families of children with cerebral palsy. *41*(5), 531-542.

Whittingham, K., Sheffield, J., & Boyd, R. N. J. B. o. (2016). Parenting acceptance and commitment therapy: a randomised controlled trial of an innovative online course for families of children with cerebral palsy. *6*(10), e012807.

Whittingham, K., Sheffield, J., Mak, C., Wright, A., Boyd, R. N. J. B. R., & Therapy. (2022). Parenting Acceptance and Commitment Therapy: An RCT of an online course with families of children with CP. 104129.

Wong, F. K. D., Poon, A. J. A., & Psychiatry, N. Z. J. o. (2010). Cognitive behavioural group treatment for Chinese parents with children with developmental disabilities in Melbourne, Australia: An efficacy study. *44*(8), 742-749.


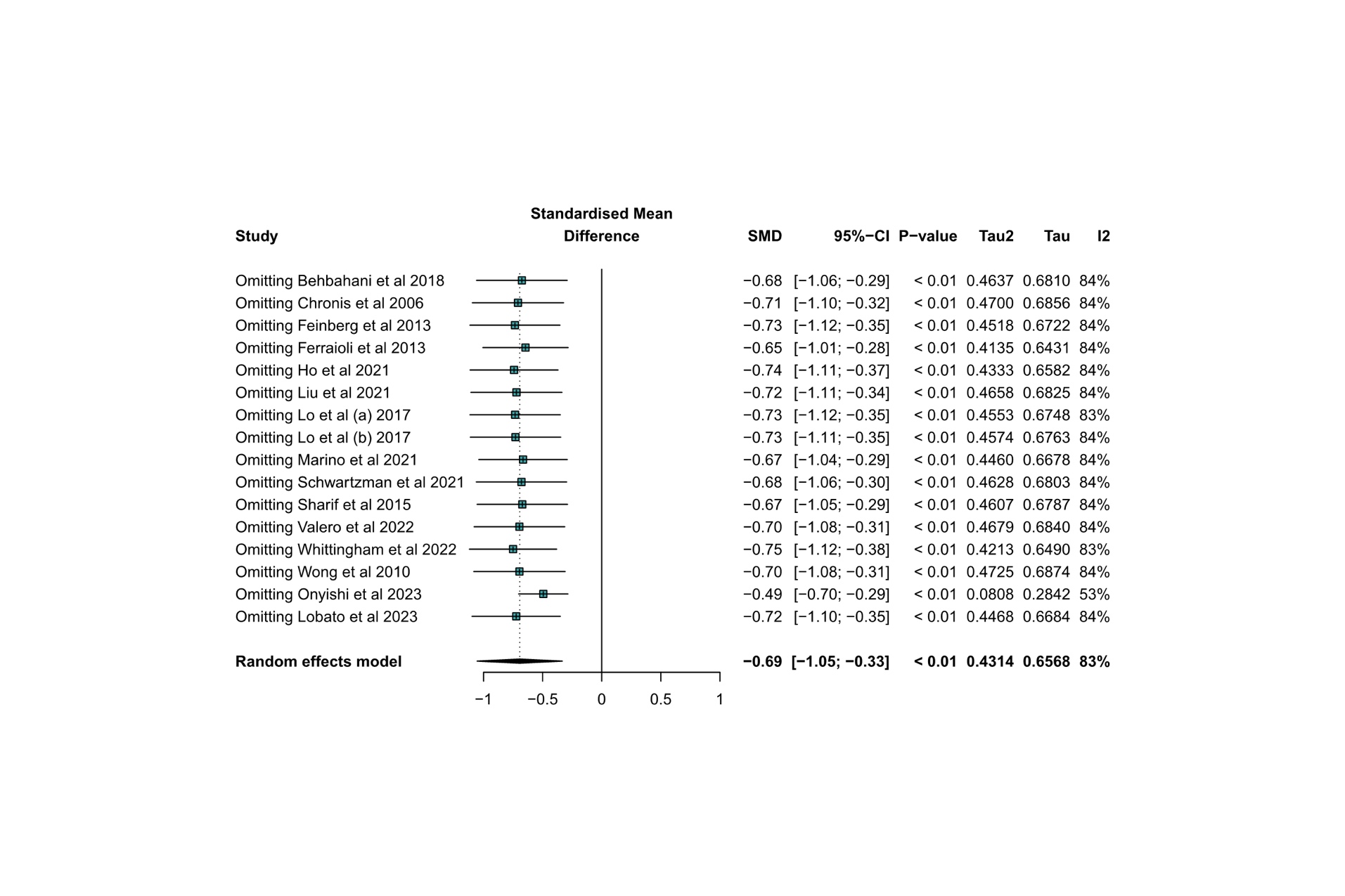


**Supplementary Figure 1.** Sensitivity analysis for the effects of cognitive-based interventions on parental stress

*Egger test -Test for Funnel Plot Asymmetry: t=-1.63, p=0.1255

**Supplementary Figure 2.** Funnel plots for the effects of cognitive-based interventions on parental stress.


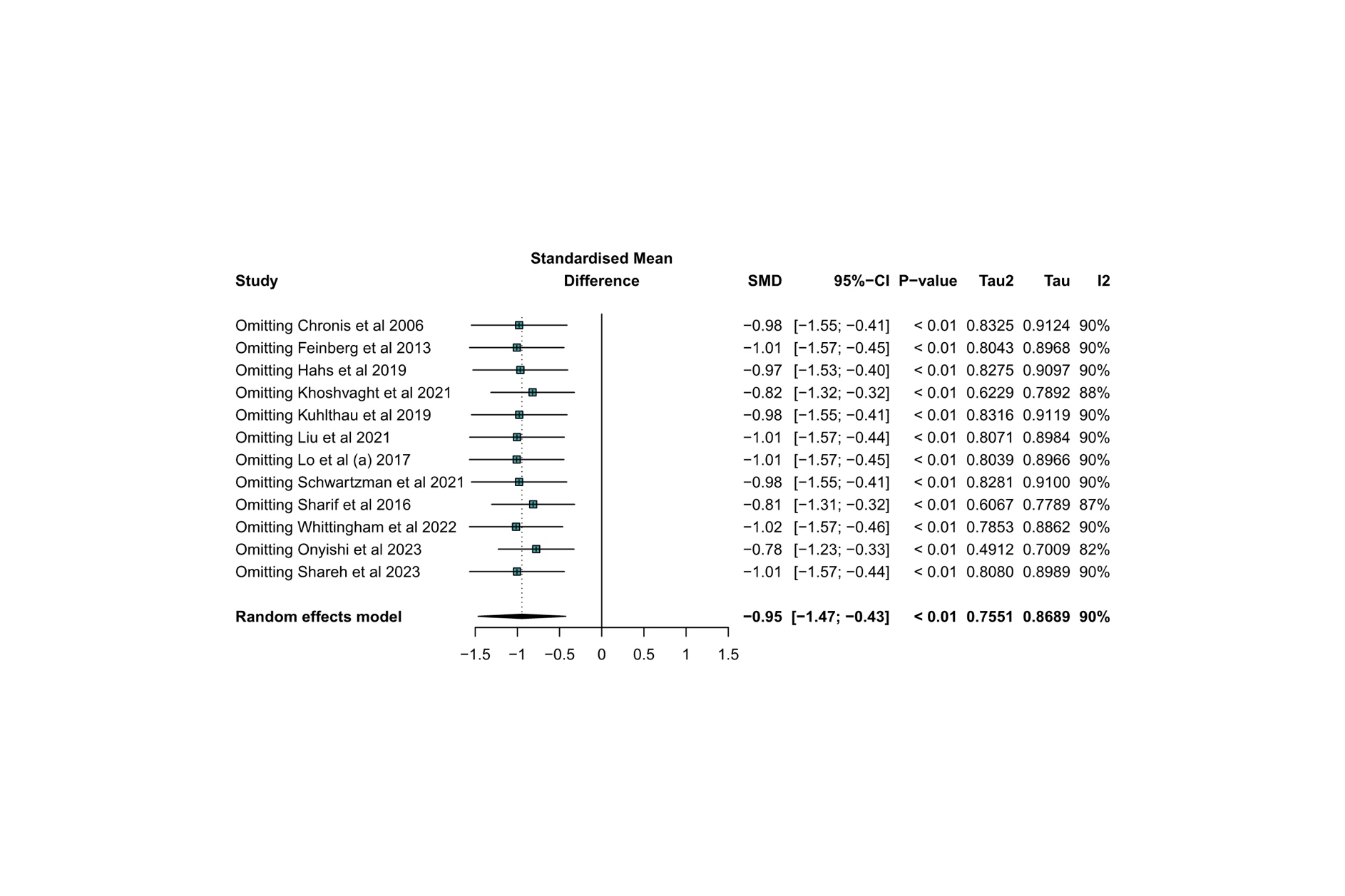


**Supplementary Figure 3.** Sensitivity analysis for the effects of cognitive-based interventions on depressive symptoms.

*Egger test -Test for Funnel Plot Asymmetry: t=-2.27, p=0.0467

**Supplementary Figure 4.** Funnel plots for the effects of cognitive-based interventions on depressive symptoms.


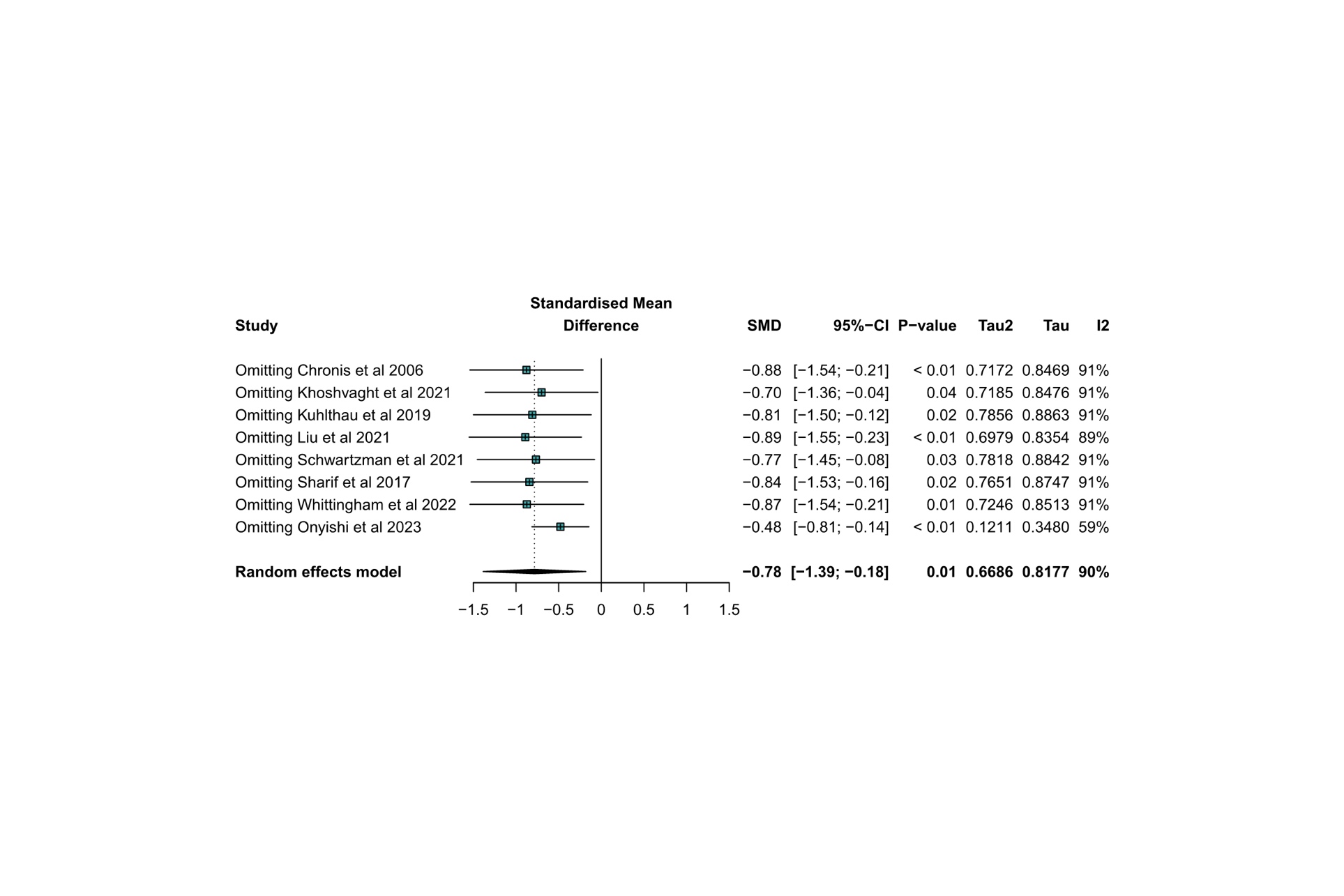


**Supplementary Figure 5.** Sensitivity analysis for the effects of cognitive-based interventions on anxiety.


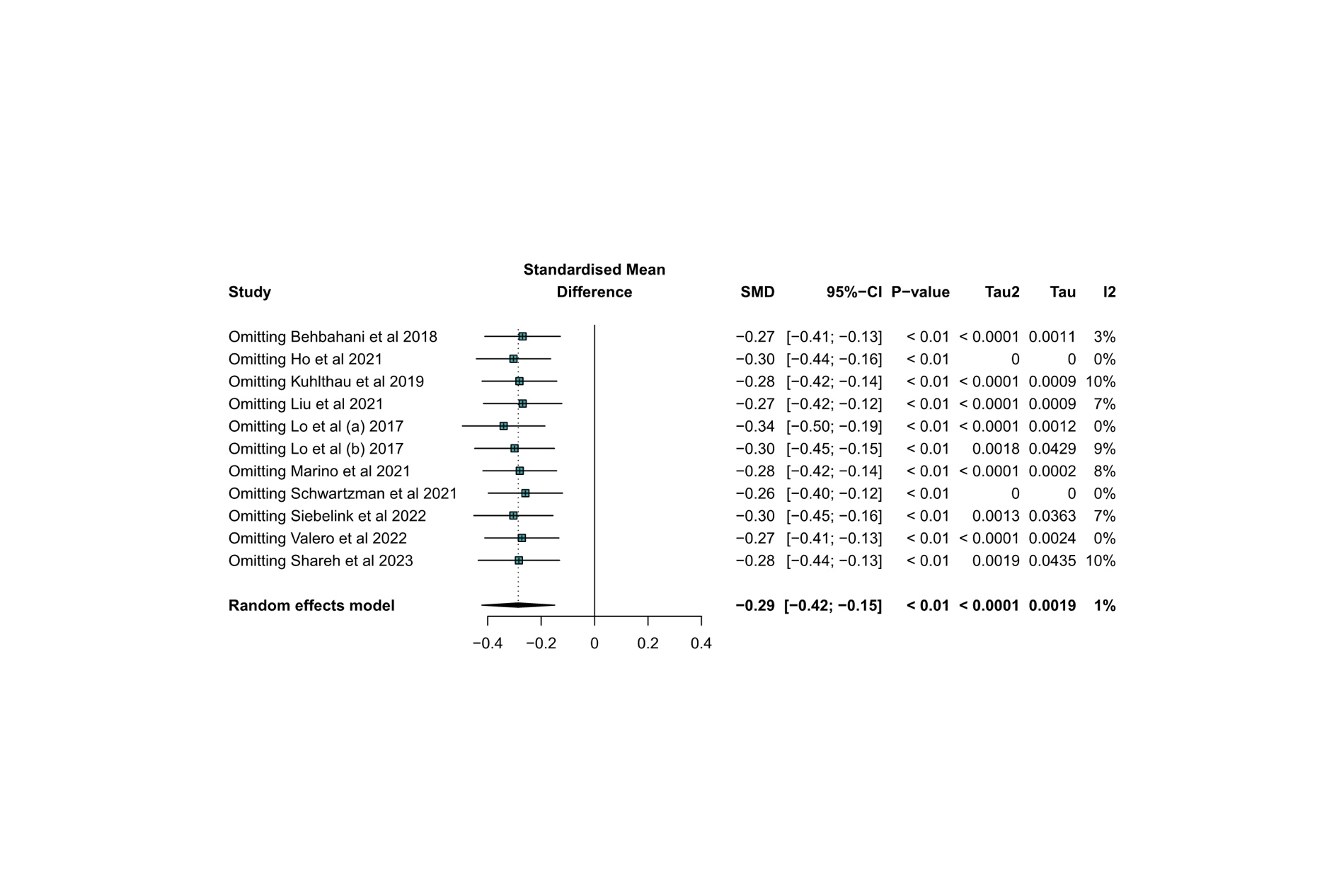


**Supplementary Figure 6.** Sensitivity analysis for the effects of cognitive-based interventions on parental distress.

*Egger test -Test for Funnel Plot Asymmetry: t=-1.81, p=0.1042

**Supplementary Figure 7.** Funnel plots for the effects of cognitive-based interventions on parental distress.


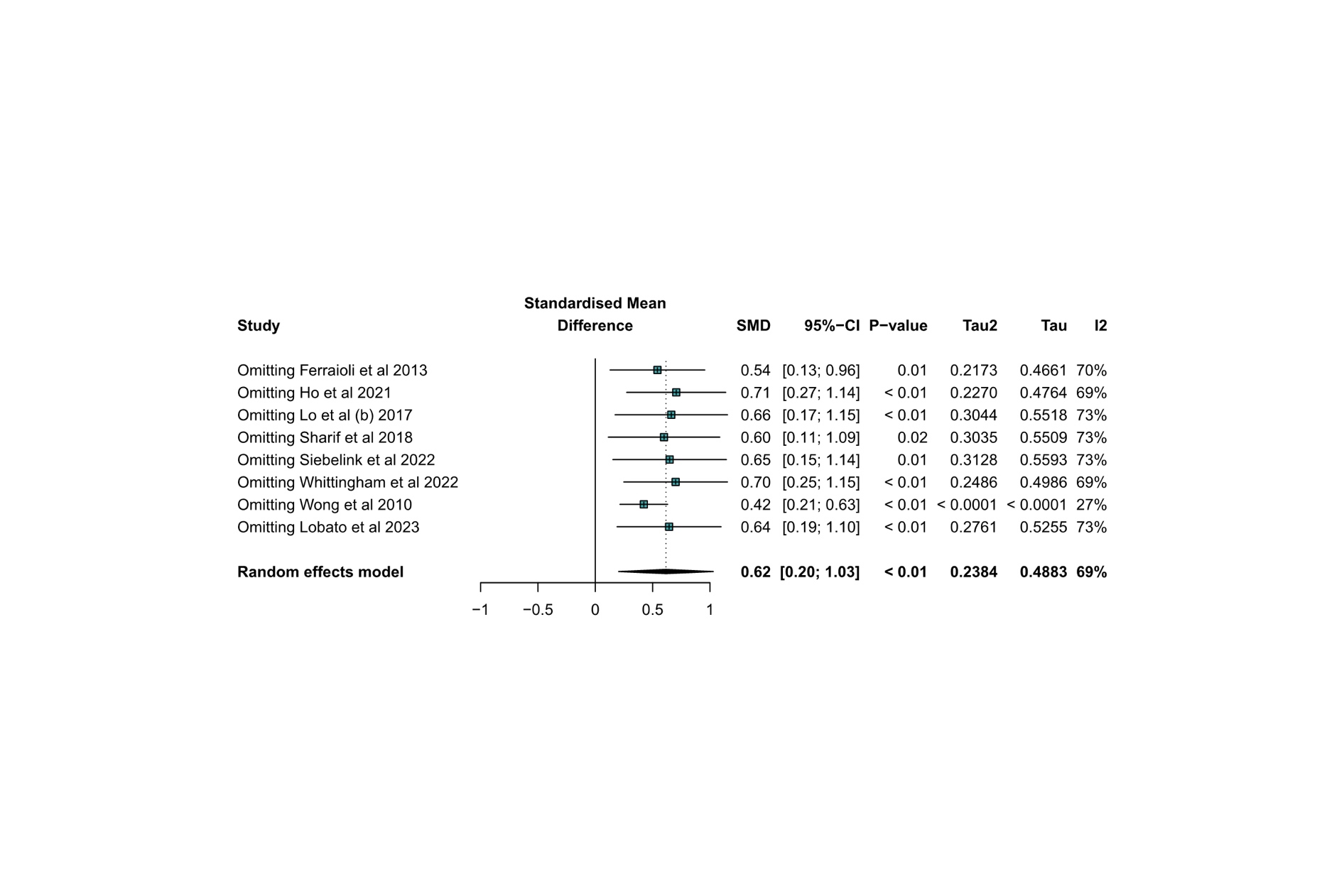


**Supplementary Figure 8.** Sensitivity analysis for the effects of cognitive-based interventions on parental well-being of parents.


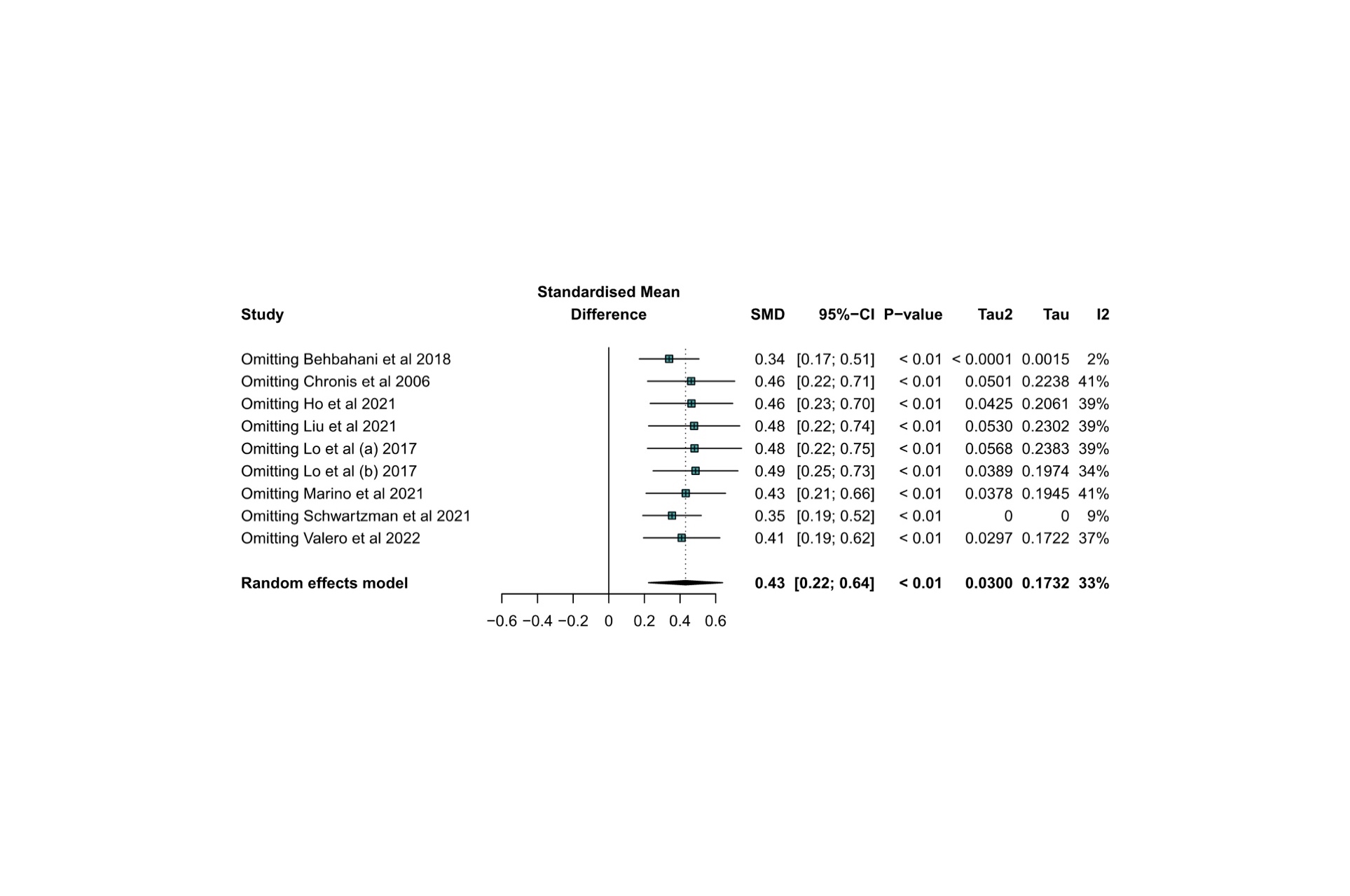


**Supplementary Figure 9.** Sensitivity analysis for the effects of cognitive-based interventions on parent-child relationship of children with ASD.

**
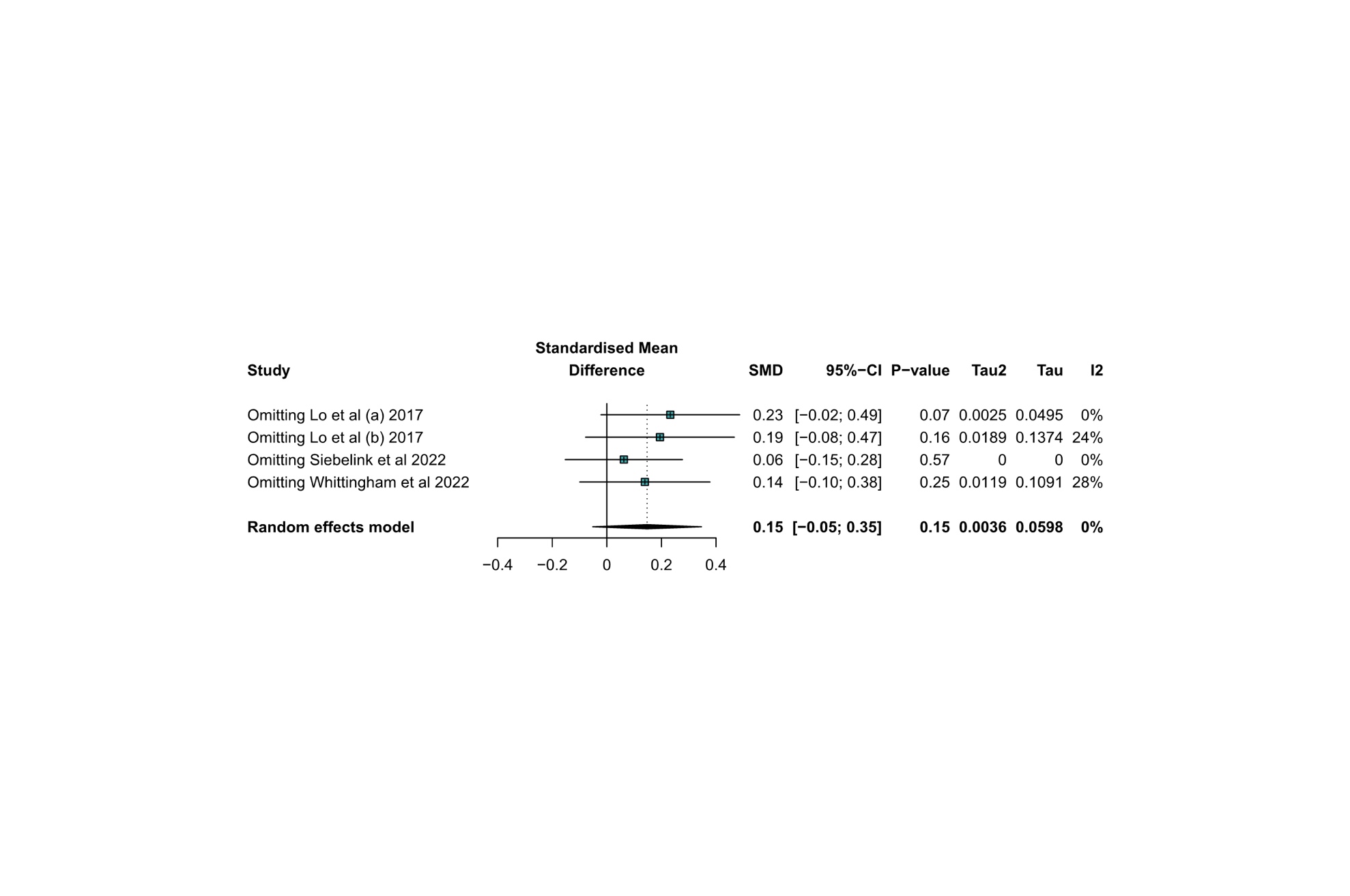
**

**Supplementary Figure 10.** Sensitivity analysis for the effects of cognitive-based interventions on mindful parenting of children with ASD.

**
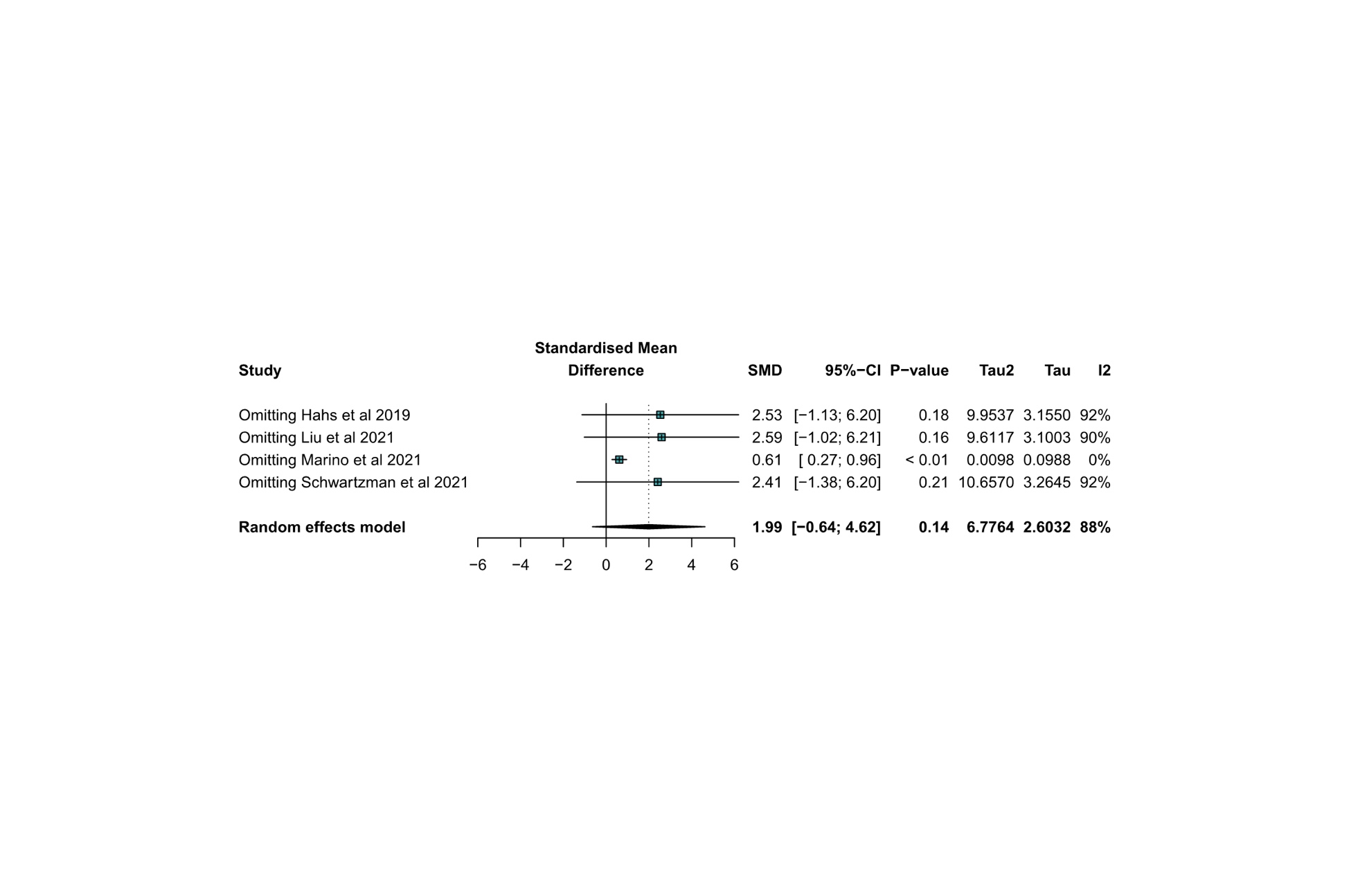
**

**Supplementary Figure 11.** Sensitivity analysis for the effects of cognitive-based interventions on mindful awareness of children with ASD.

**
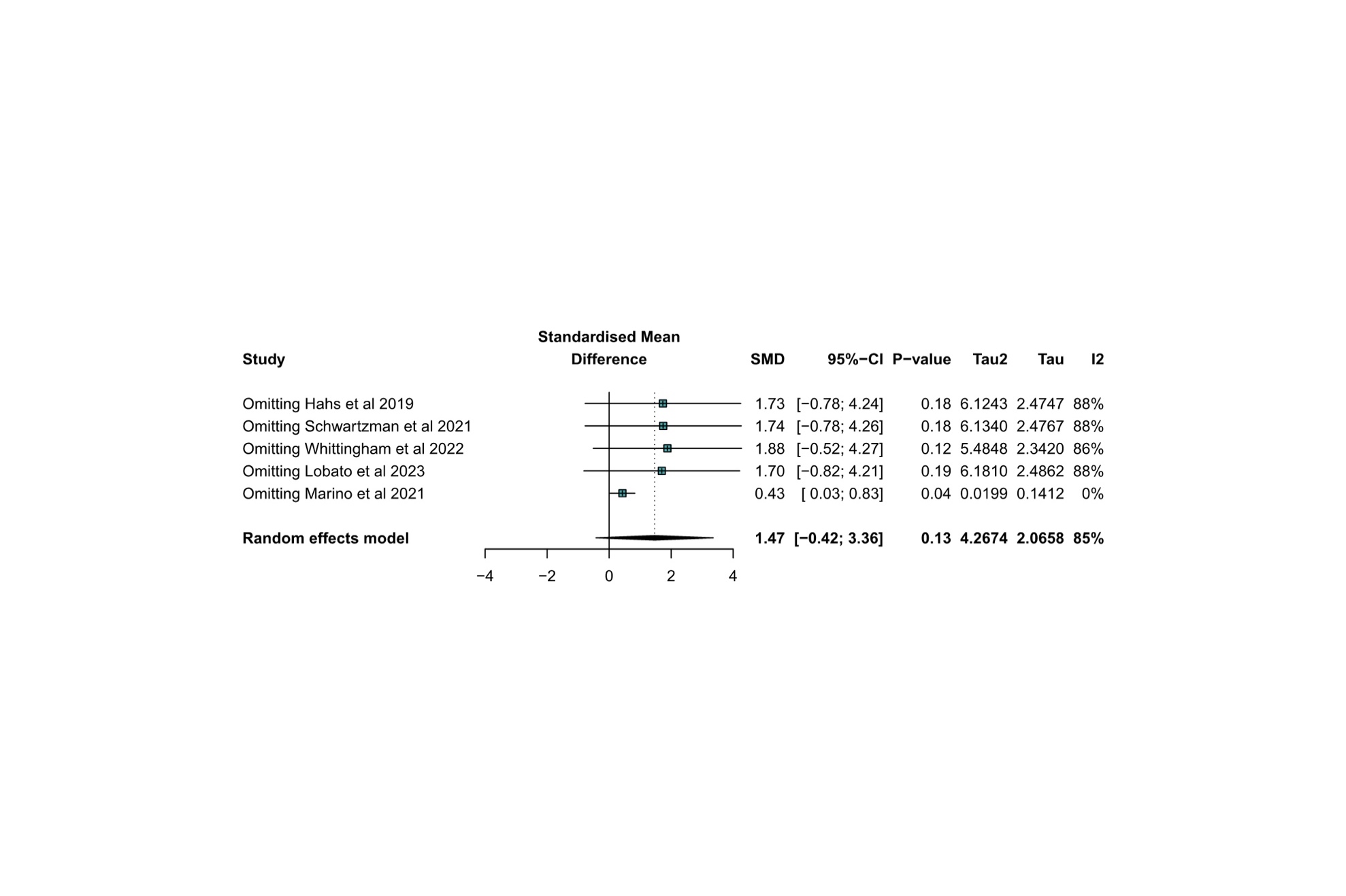
**

**Supplementary Figure 12.** Sensitivity analysis for the effects of cognitive-based interventions on psychological flexibility of children with ASD.


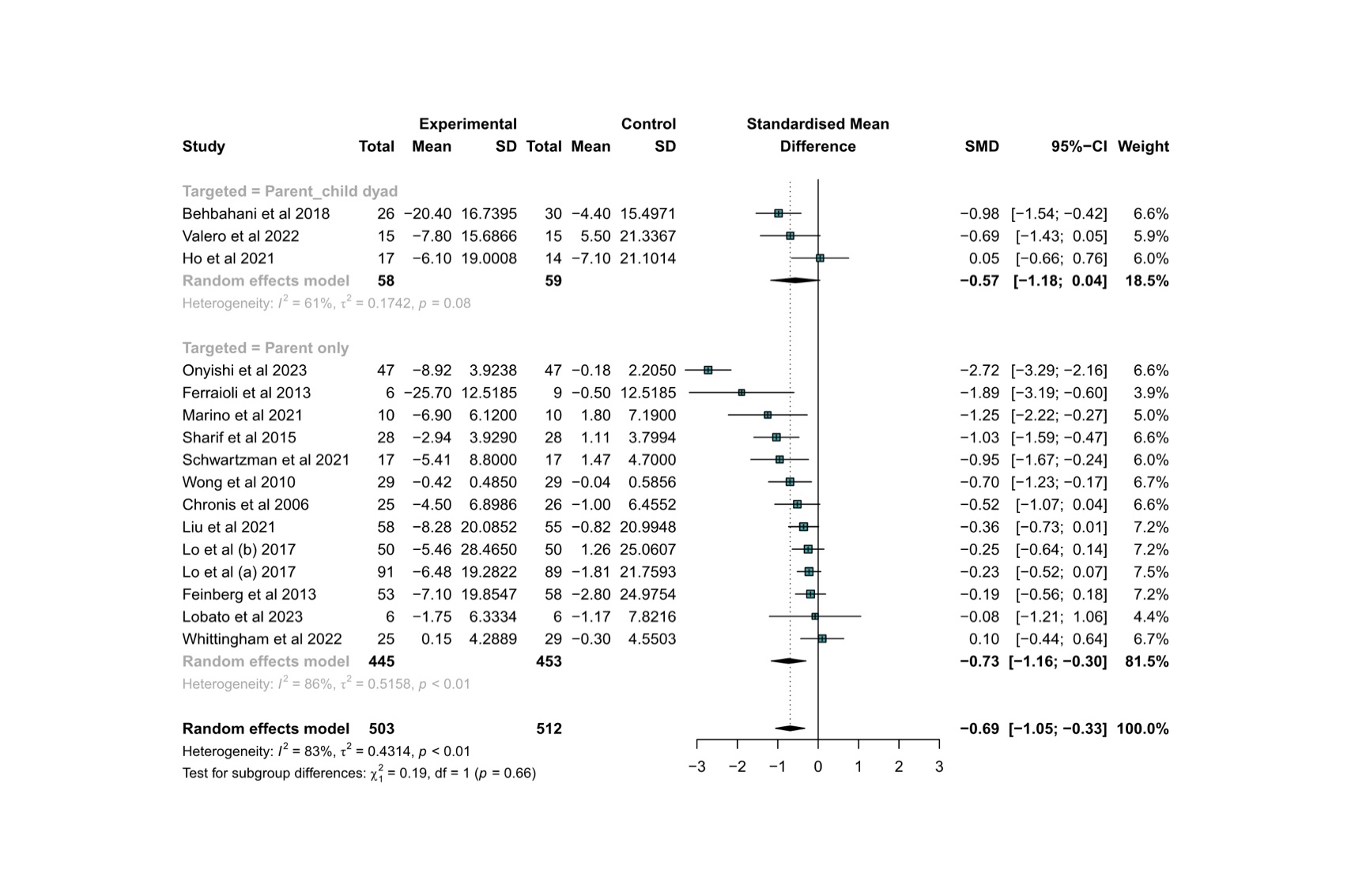


**Supplementary Figure 13.** Subgroup analysis for the effects of cognitive-based interventions on parental stress based on different targeted participants.

**
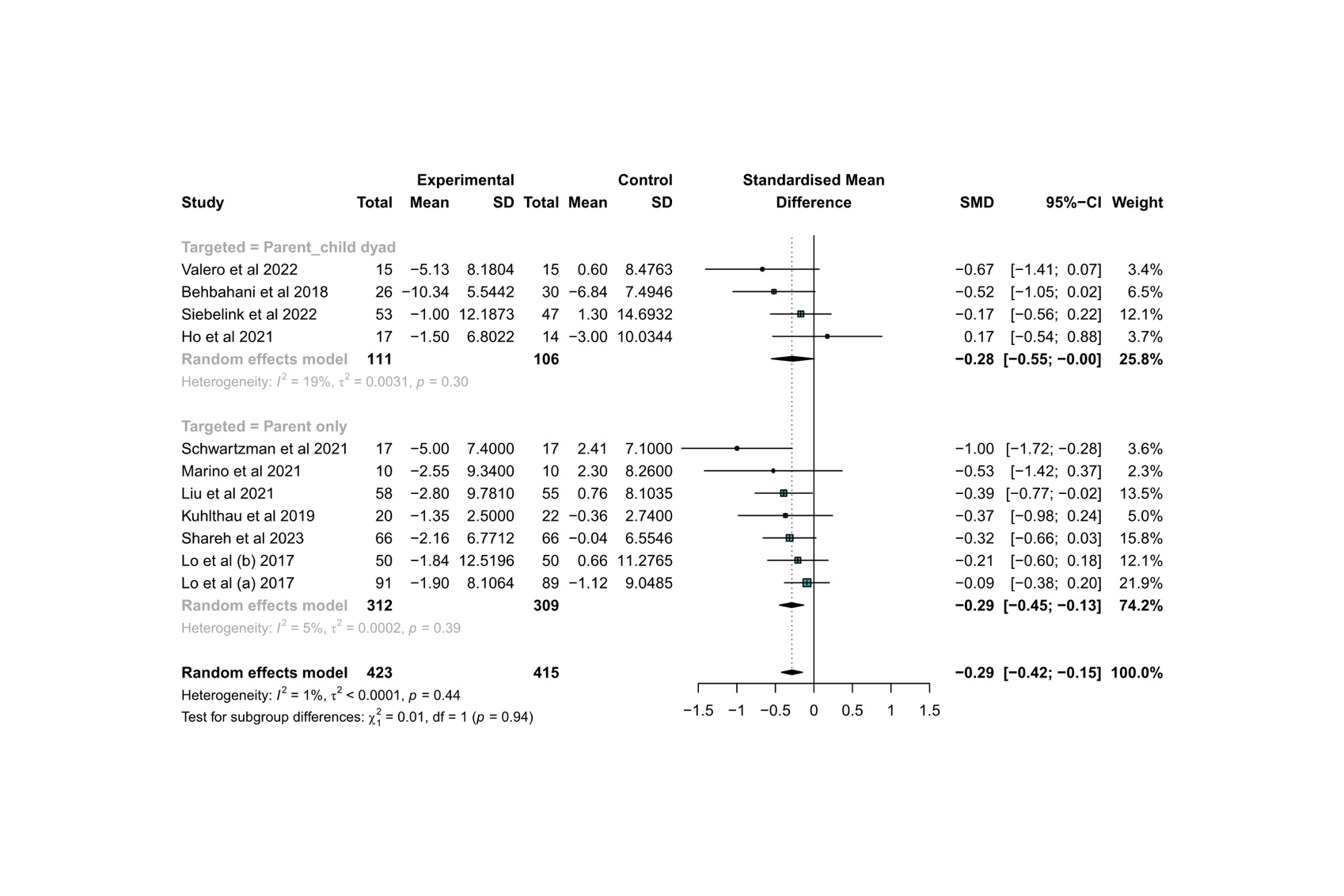
**

**Supplementary Figure 14.** Subgroup analysis for the effects of cognitive-based interventions on parental distress based on different targeted participants.


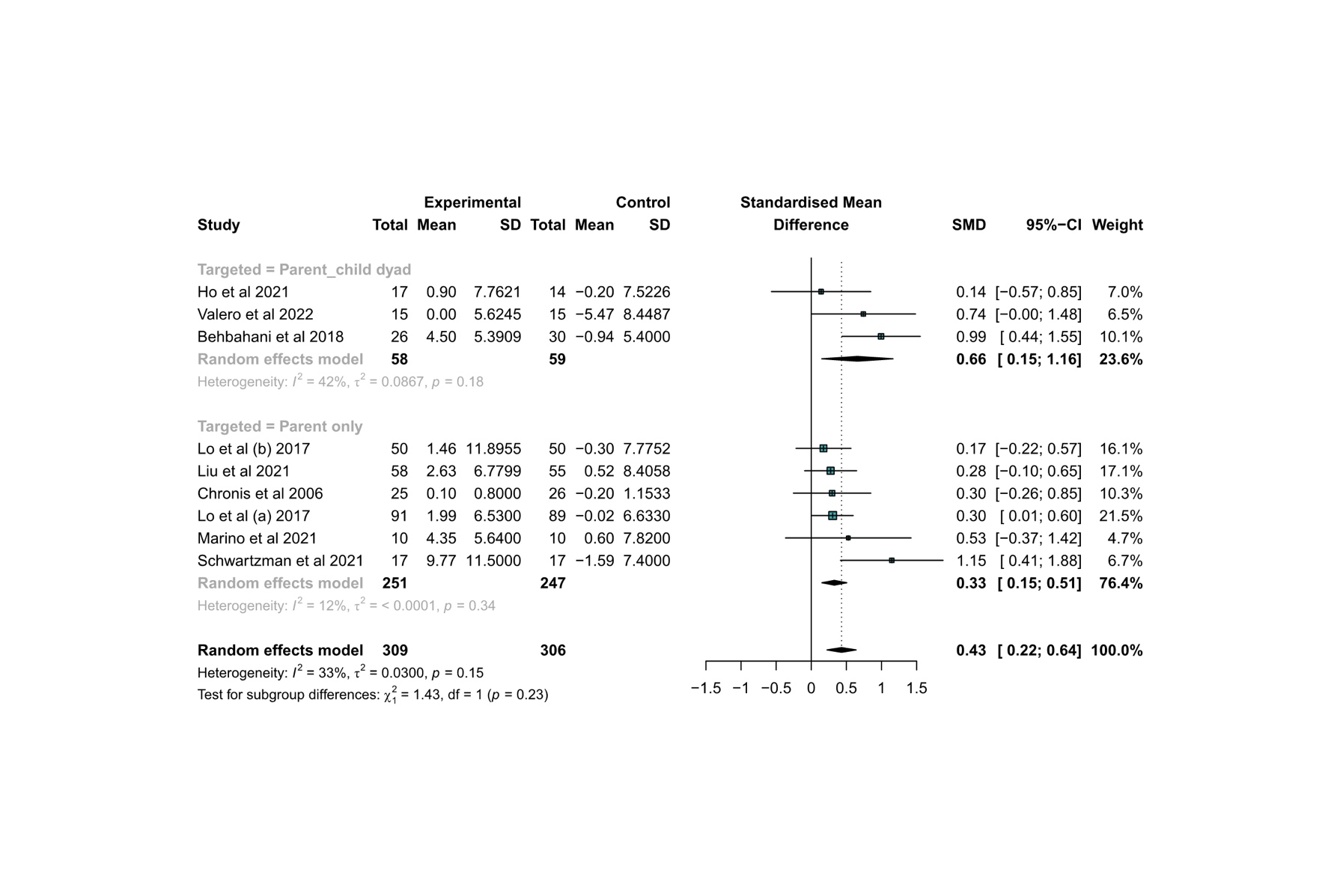


**Supplementary Figure 15.** Subgroup analysis for the effects of cognitive-based interventions on parent-child relationship based on different targeted participants.


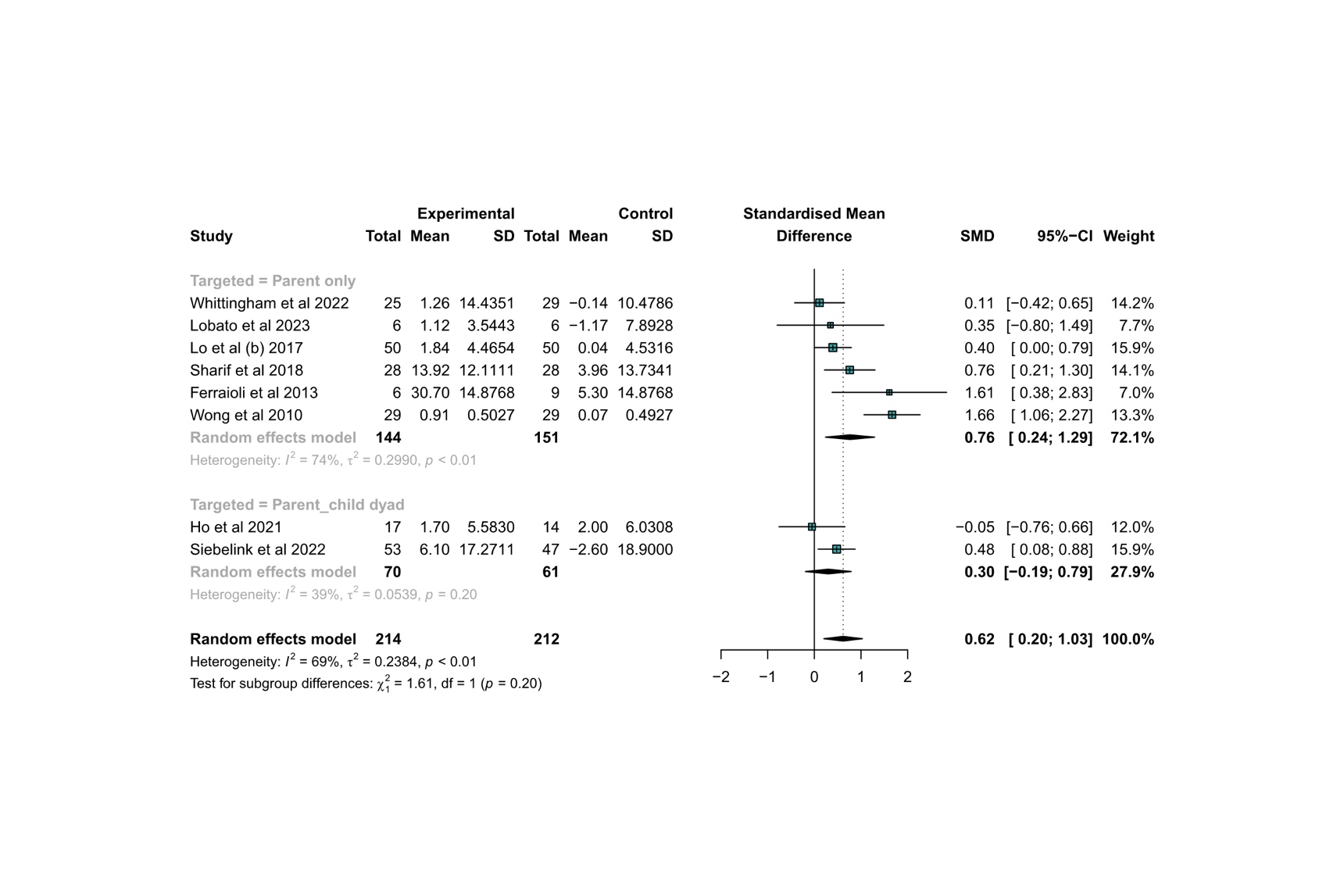


**Supplementary Figure 16.** Subgroup analysis for the effects of cognitive-based interventions on parental well-being based on different targeted participants.


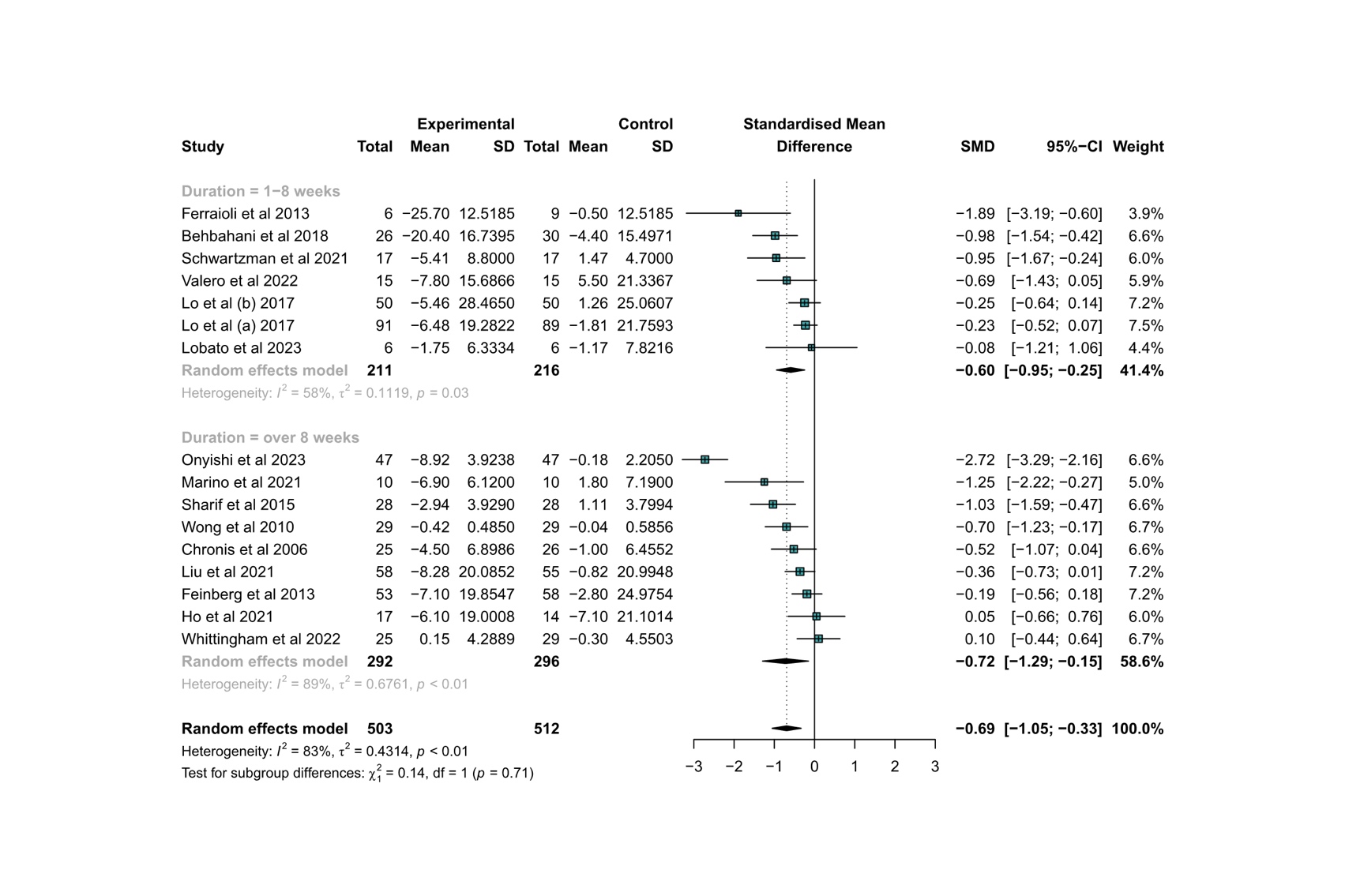


**Supplementary Figure 17.** Subgroup analysis for the effects of cognitive-based interventions on parental stress based on treatment duration.


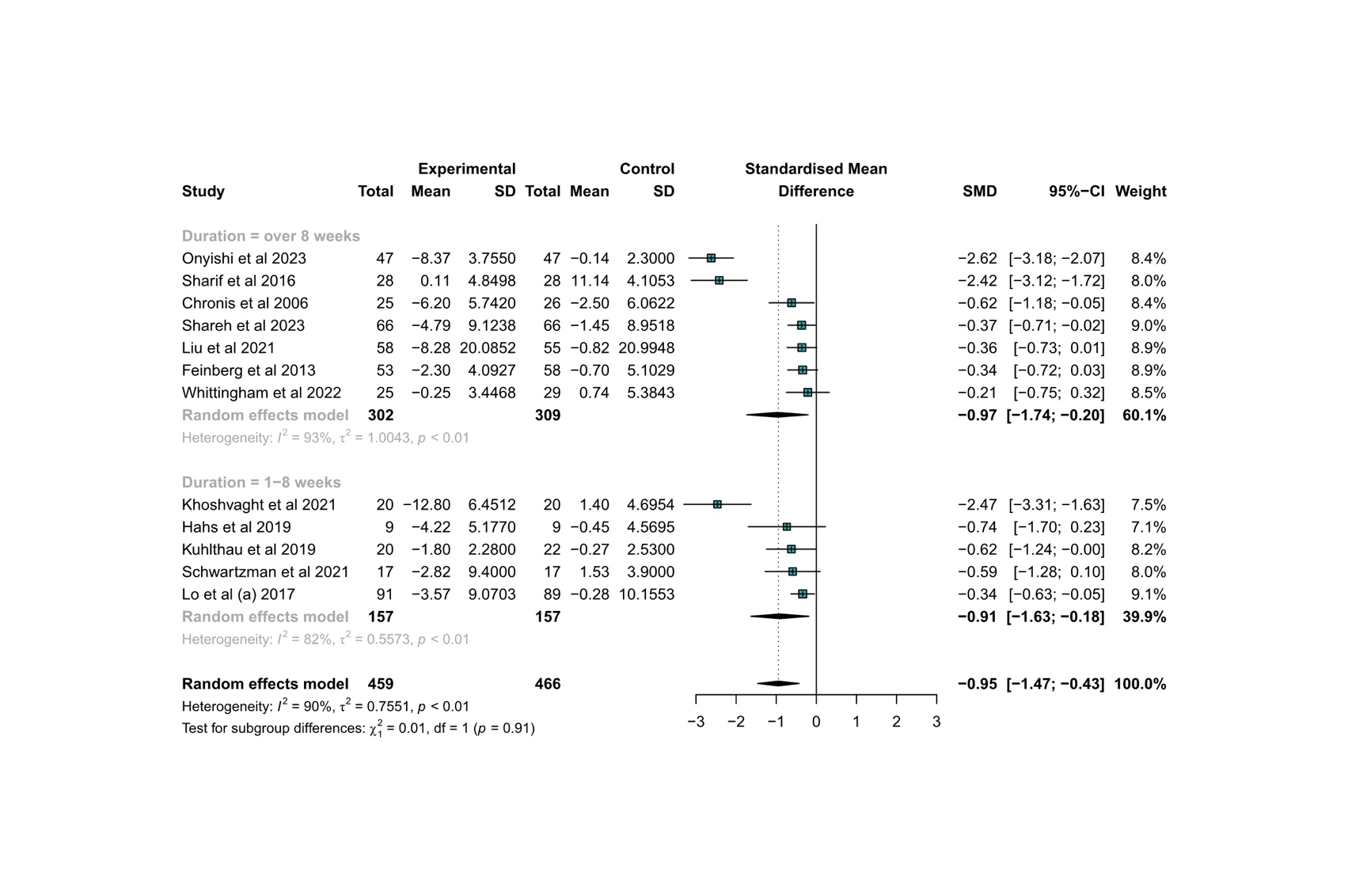


**Supplementary Figure 18.** Subgroup analysis for the effects of cognitive-based interventions on depressive symptoms based on treatment duration.


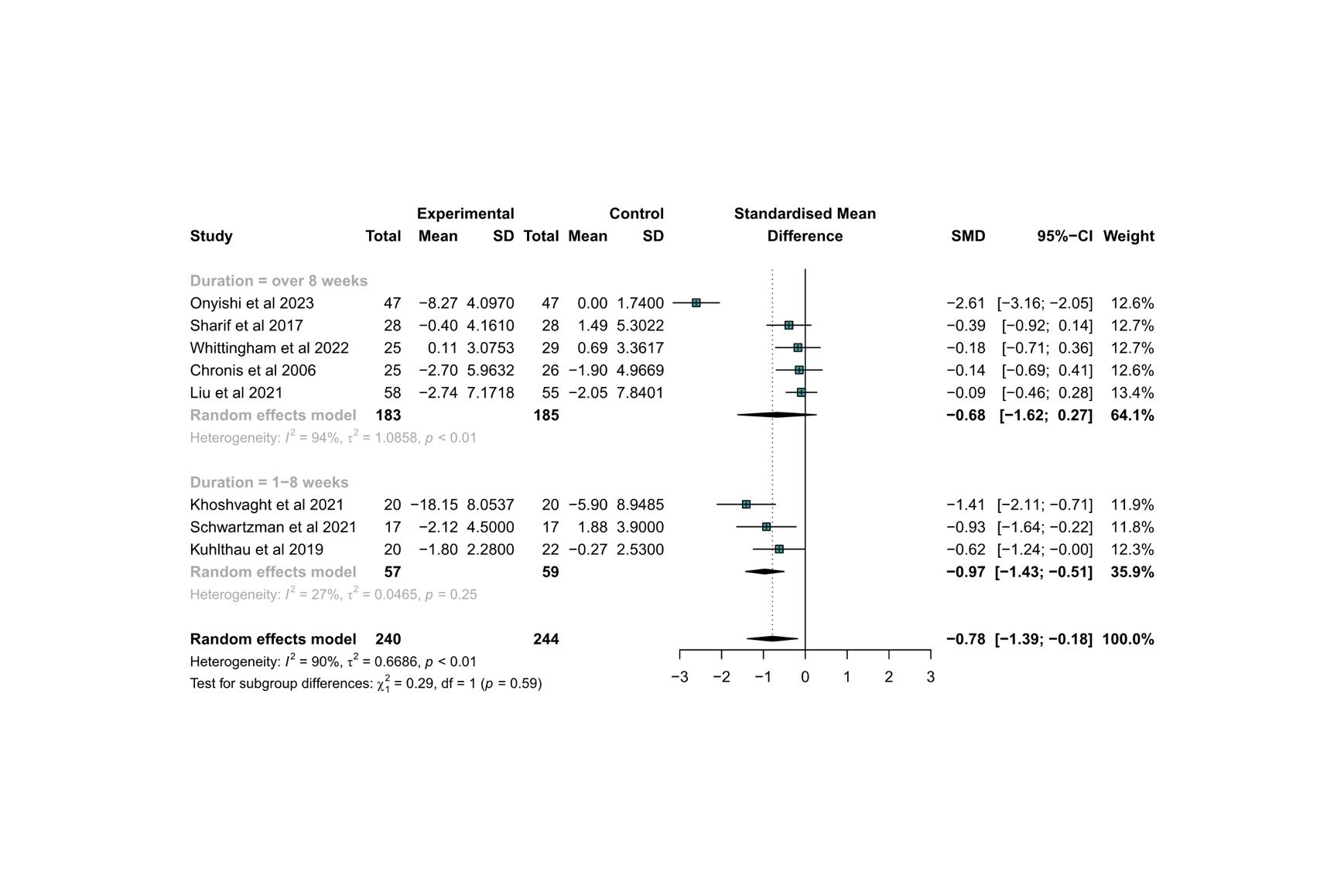


**Supplementary Figure 19.** Subgroup analysis for the effects of cognitive-based interventions on anxiety based on treatment duration.


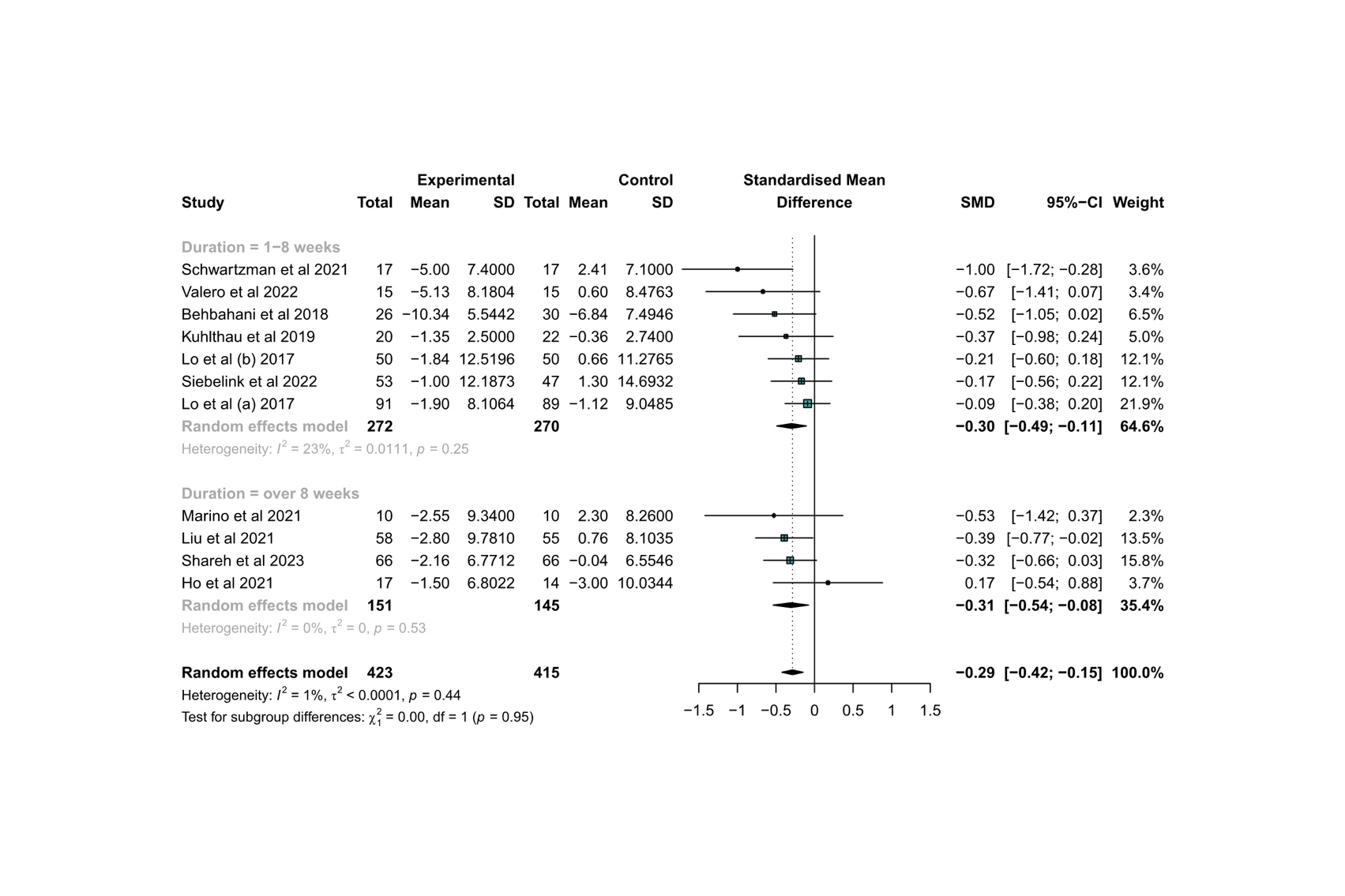


**Supplementary Figure 20.** Subgroup analysis for the effects of cognitive-based interventions on parental distress based on treatment duration.


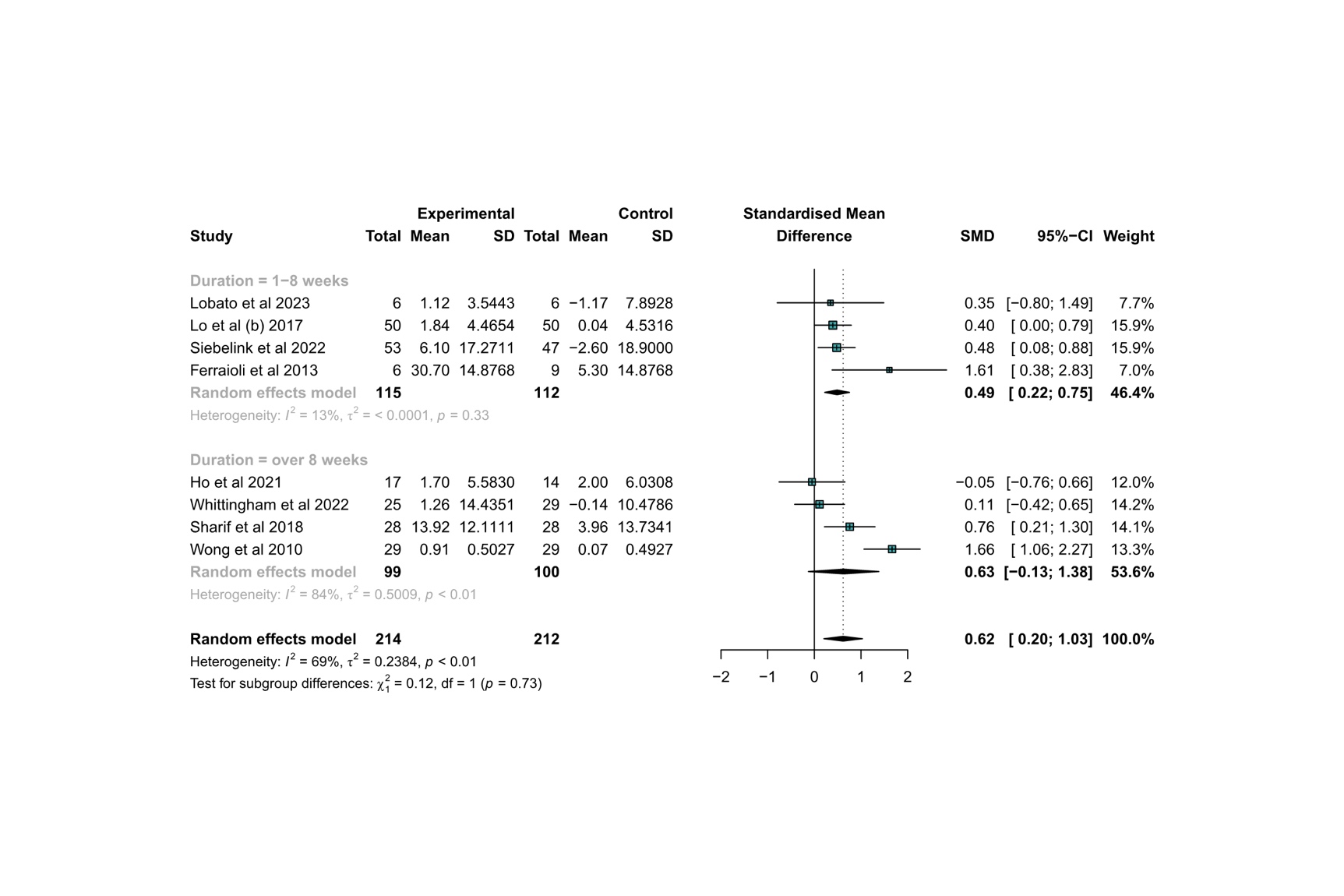


**Supplementary Figure 21.** Subgroup analysis for the effects of cognitive-based interventions on parental well-being based on treatment duration.


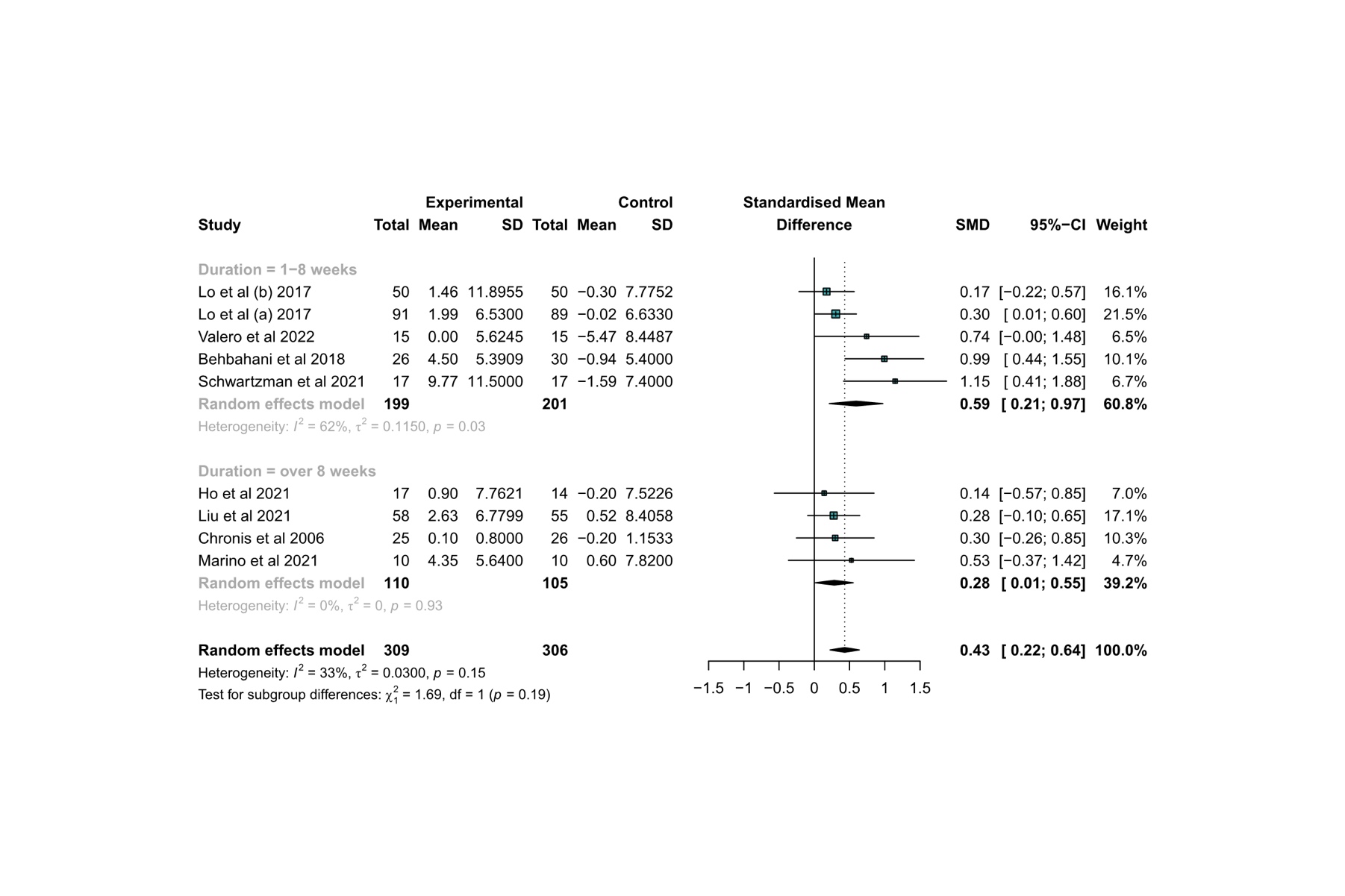


**Supplementary Figure 22.** Subgroup analysis for the effects of cognitive-based interventions on parent-child relationship based on treatment duration.


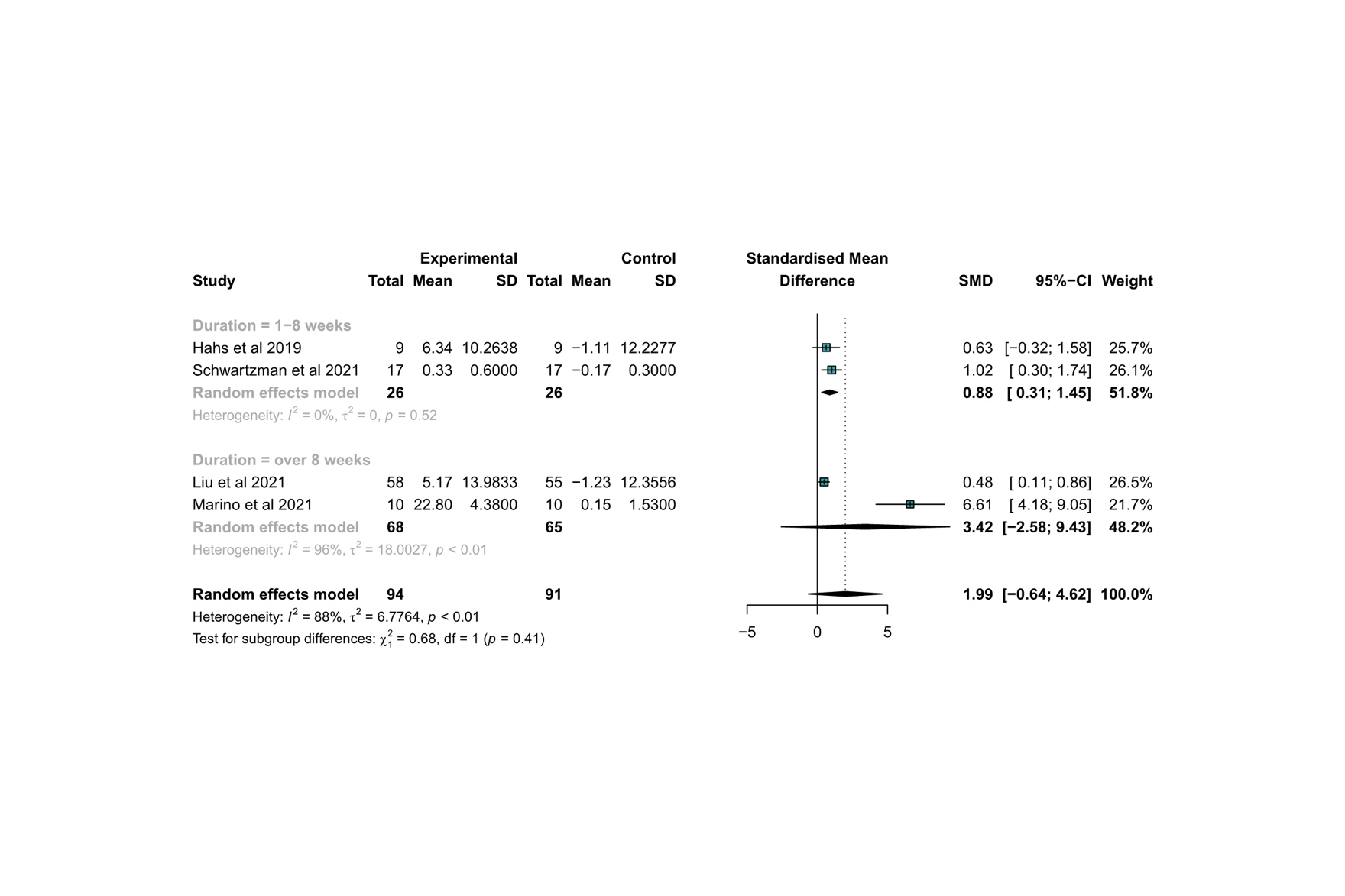


**Supplementary Figure 23.** Subgroup analysis for the effects of cognitive-based interventions on mindfulness awareness based on treatment duration.


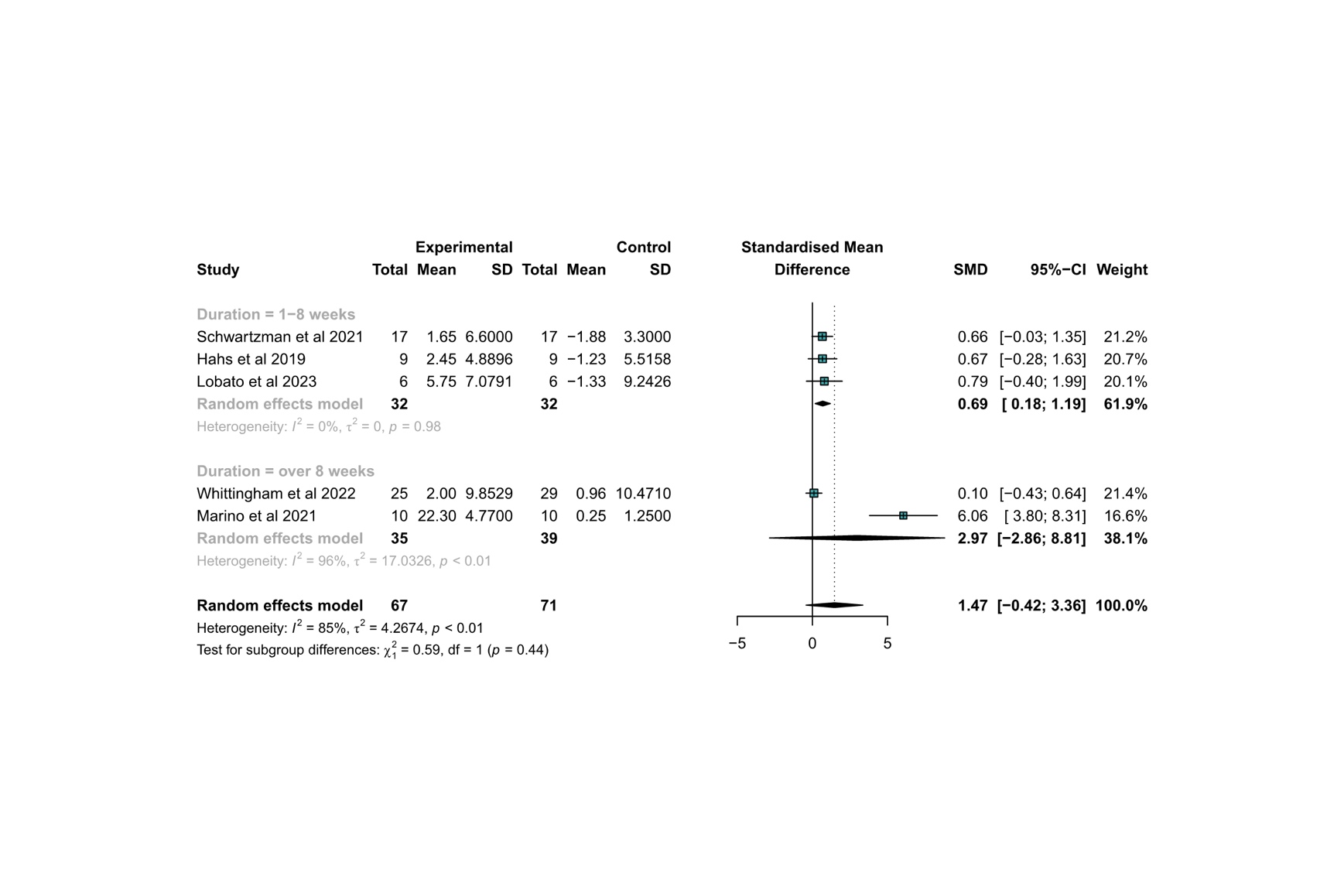


**Supplementary Figure 24.** Subgroup analysis for the effects of cognitive-based interventions on parental psychological flexibility based on treatment duration.


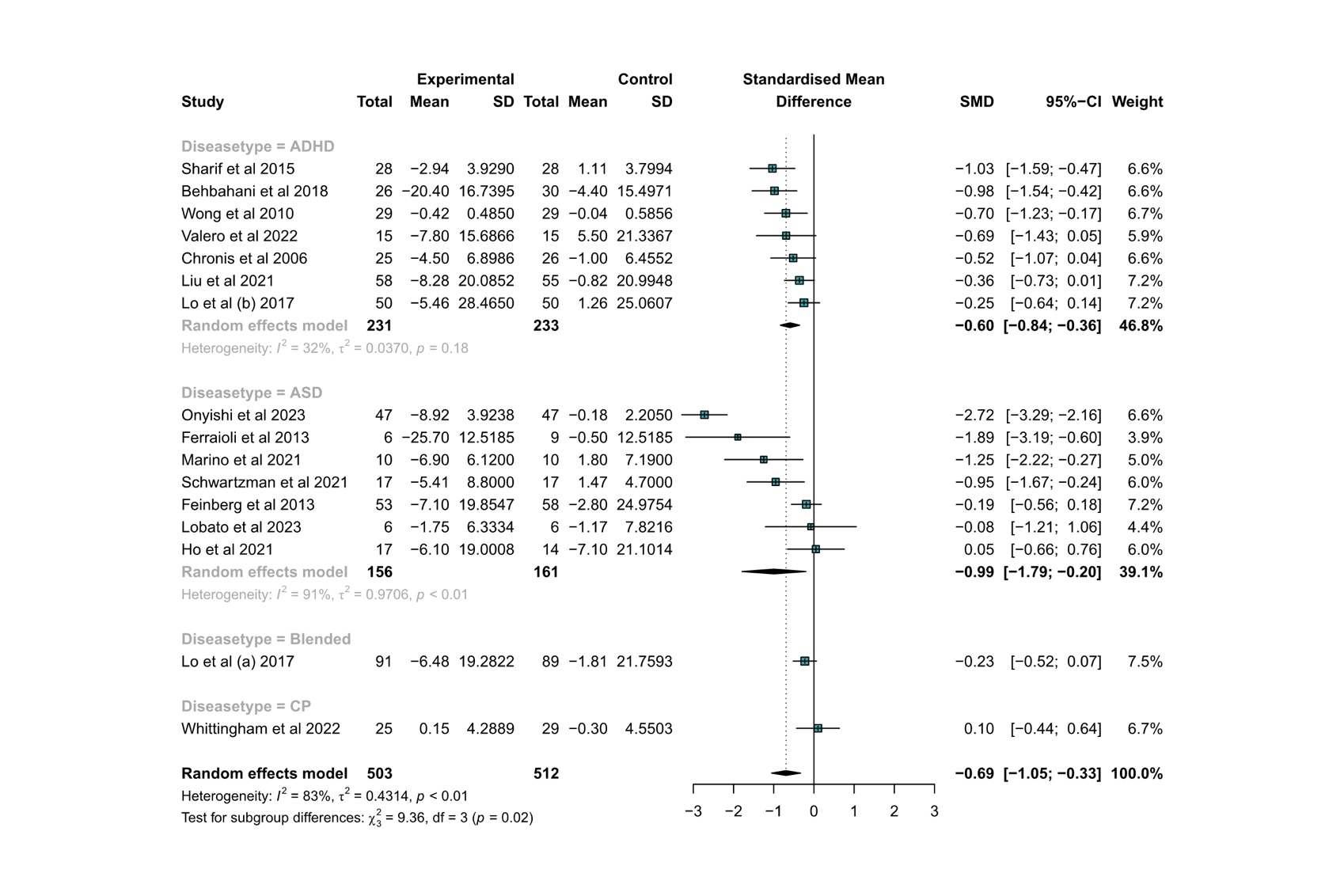


**Supplementary Figure 25.** Subgroup analysis for the effects of cognitive-based interventions on parental stress based on disease types of children.


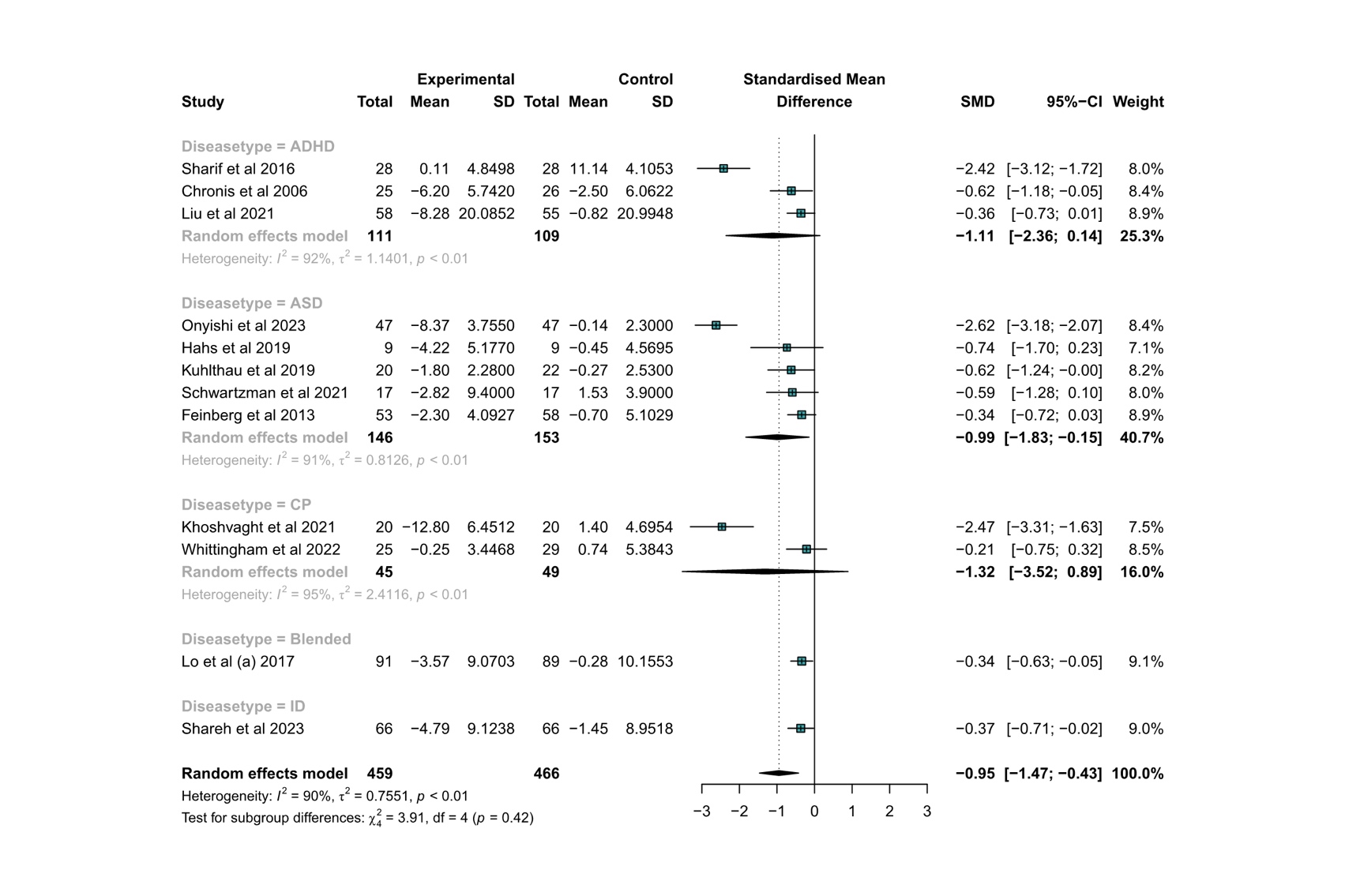


**Supplementary Figure 26.** Subgroup analysis for the effects of cognitive-based interventions on depressive symptoms based on disease types of children.


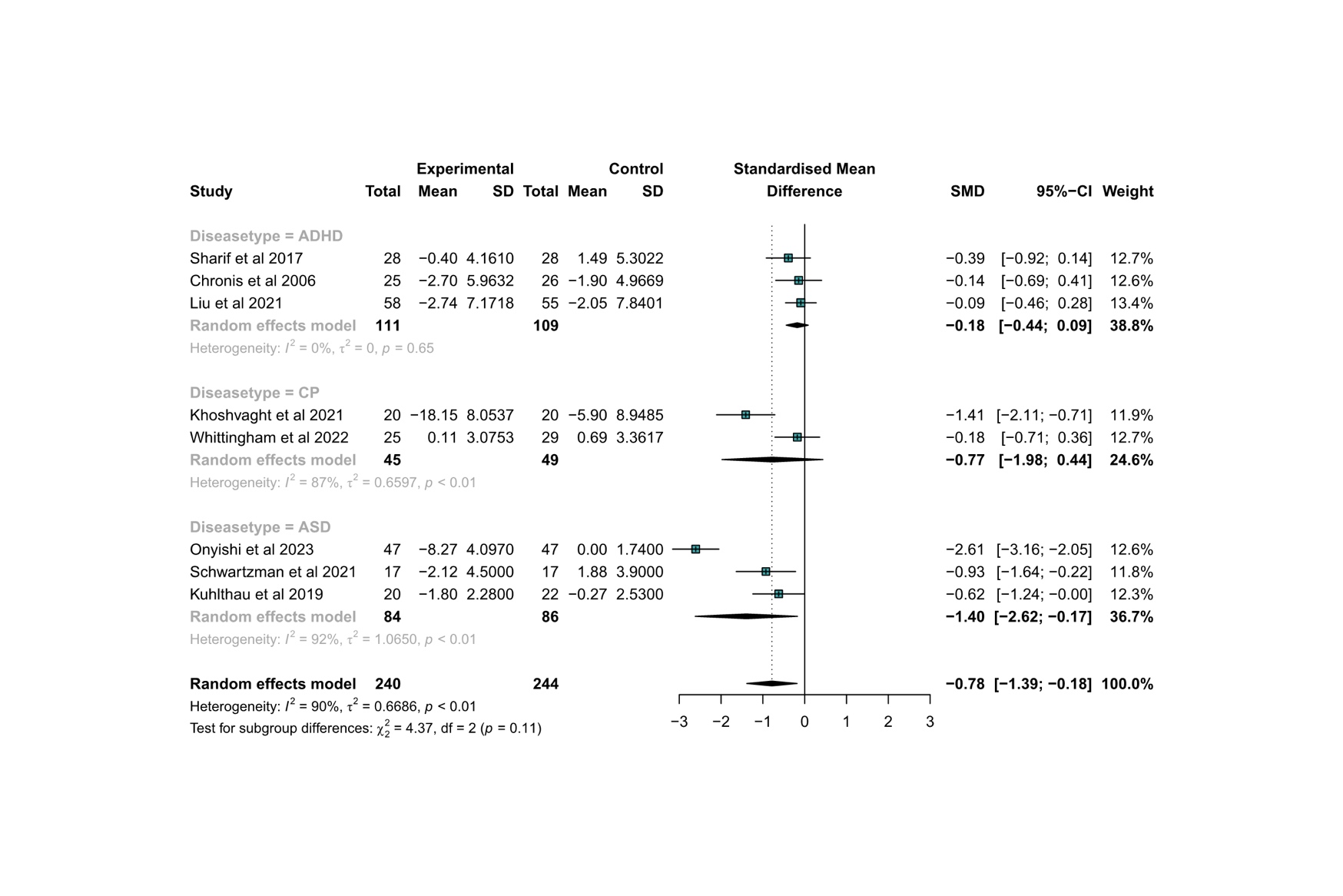


**Supplementary Figure 27.** Subgroup analysis for the effects of cognitive-based interventions on anxiety based on disease types of children.


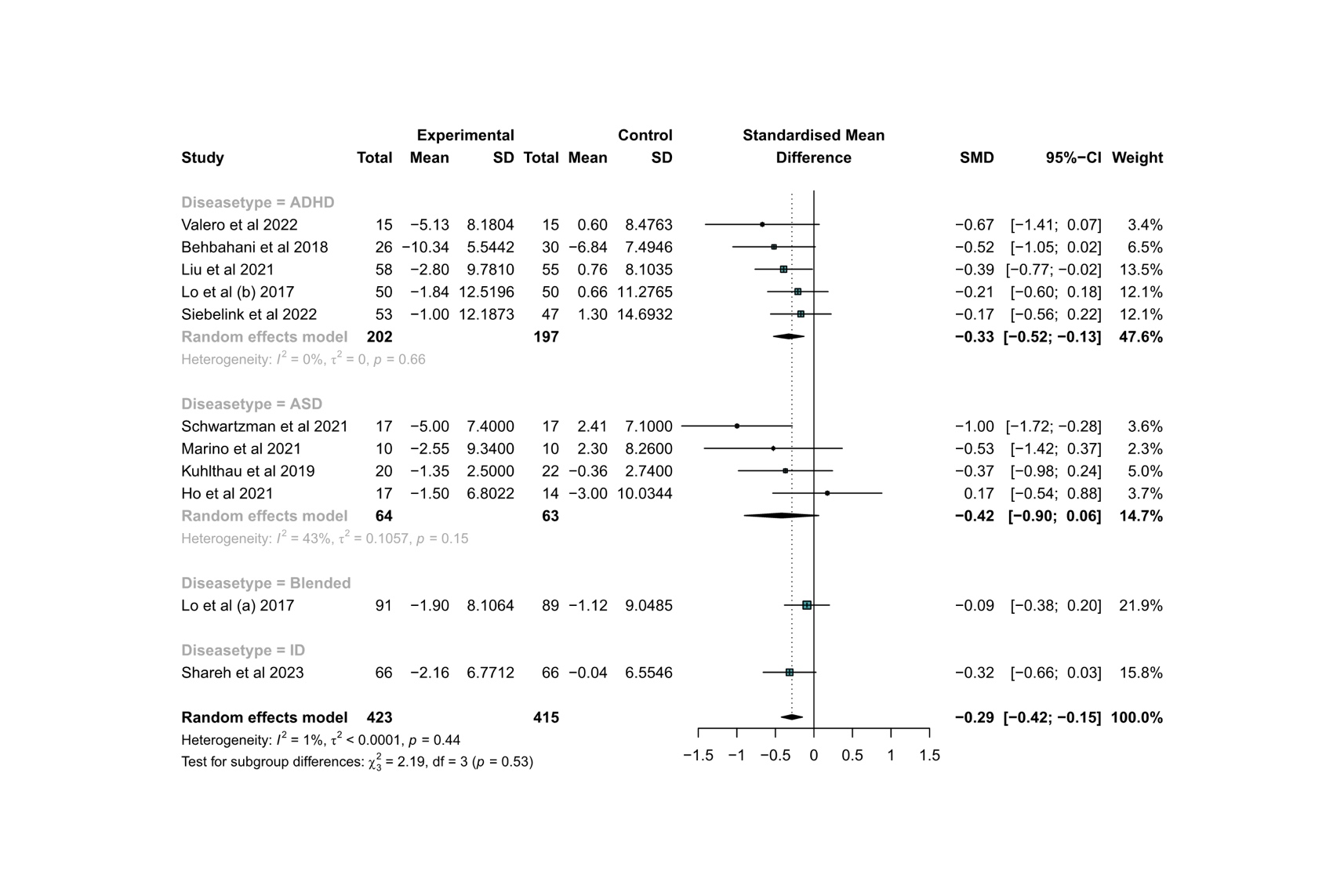


**Supplementary Figure 28.** Subgroup analysis for the effects of cognitive-based interventions on distress based on disease types of children.


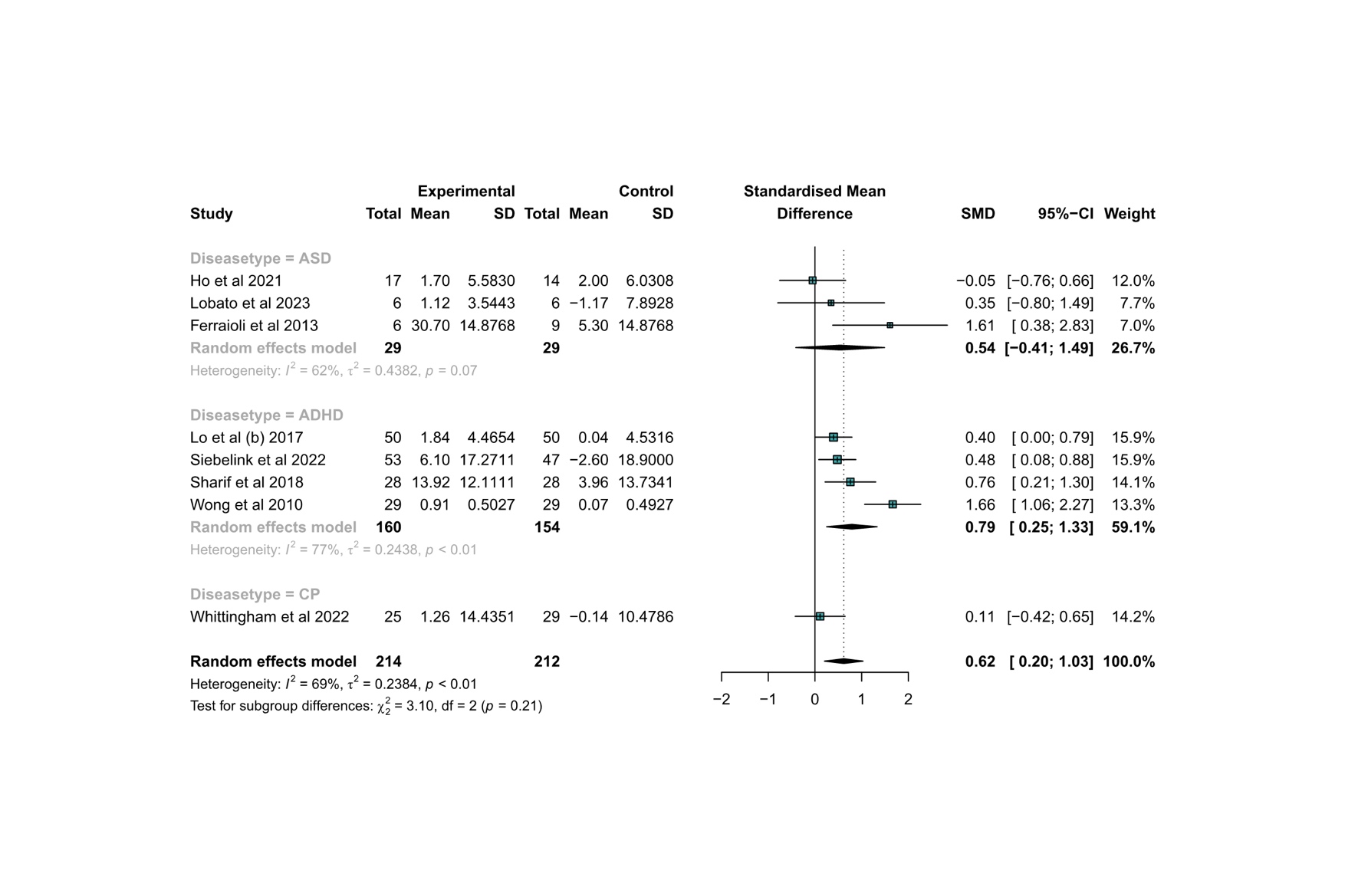


**Supplementary Figure 29.** Subgroup analysis for the effects of cognitive-based interventions on parental well-being based on disease types of children.


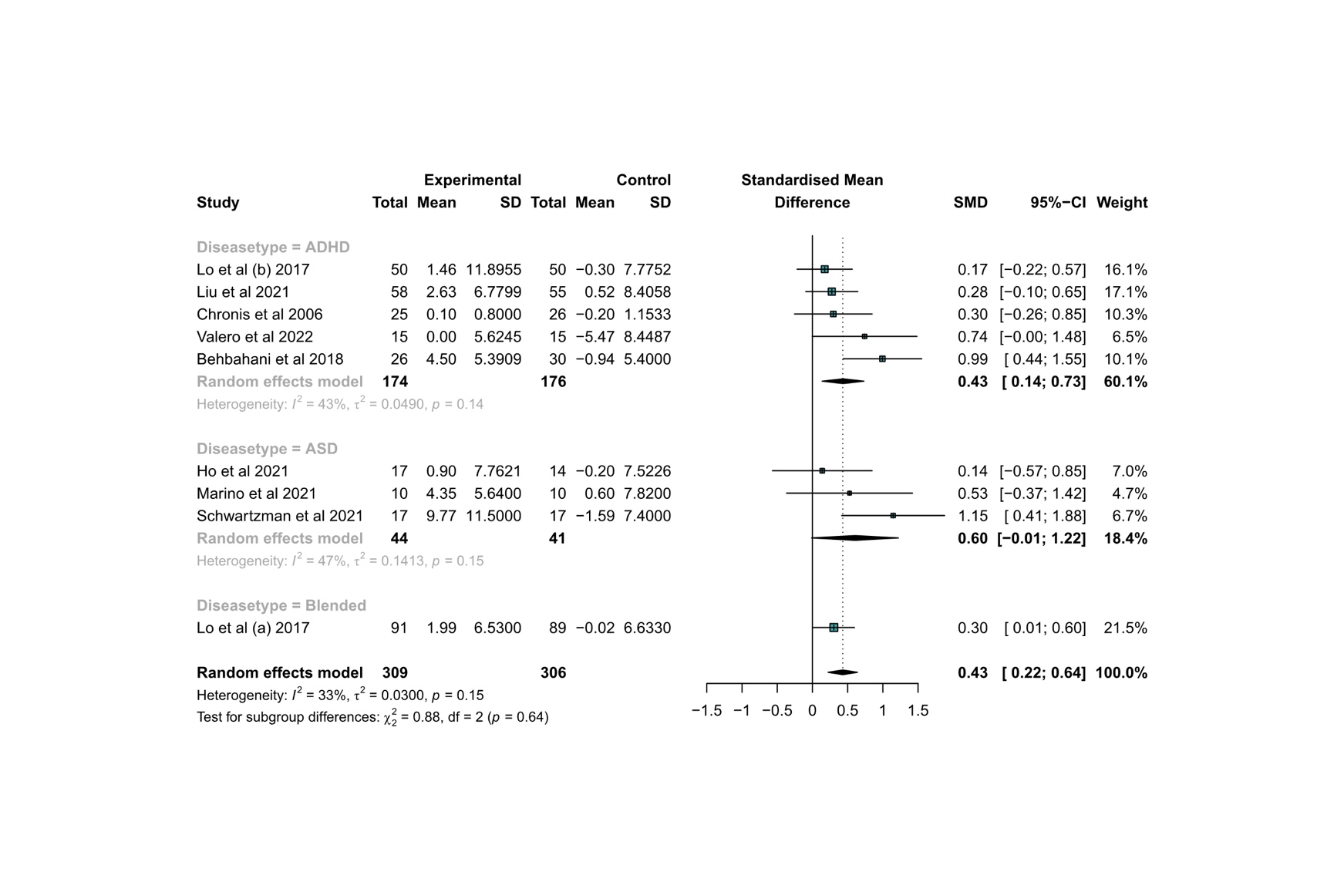


**Supplementary Figure 30.** Subgroup analysis for the effects of cognitive-based interventions on parent-child relationship based on disease types of children.
